# MicroICS: predictive phenotyping of *Listeria monocytogenes* biofilms from three-dimensional structural features

**DOI:** 10.1038/s41522-026-01083-8

**Published:** 2026-07-08

**Authors:** Nika Janež, Blaž Škrlj, Aljaž Osojnik, Márta Ladányi, Martin Breskvar, Matej Petković, Boštjan Kokot, Petra Čotar, Bojan Papić, Majda Golob, Tjaša Peternel, Jerica Sabotič

**Affiliations:** 1 Department of Biotechnology, Jožef Stefan Institute, Ljubljana, Slovenia; 2 Department of Knowledge Technologies, Jožef Stefan Institute, Ljubljana, Slovenia; 3 Department of Applied Statistics, Institute of Mathematics and Basic Science, Hungarian University of Agriculture and Life Sciences (MATE), Budapest, Hungary; 4 Department of Condensed Matter Physics, Jožef Stefan Institute, Ljubljana, Slovenia; 5 Centre for Advanced Optical Microscopies, Jožef Stefan Institute, Ljubljana, Slovenia; 6 University of Ljubljana, Veterinary Faculty, Institute of Microbiology and Parasitology, Ljubljana, Slovenia

**Keywords:** Computational biology and bioinformatics, Microbiology

## Abstract

Biofilms underpin microbial survival, yet their three-dimensional structure remains difficult to quantify systematically. We present the Microbial Image Classification Suite (MicroICS), an open-source framework for predictive phenotyping of microbial communities from 3D biofilm images. MicroICS consists of three independent but interoperable modules: feature extraction from 3D biofilm images, machine learning-based classification, and an inference module for applying trained models to new, unseen images. Each module can be run independently, and the classification module accepts externally generated features, enabling integration with existing quantitative image analysis tools. As a pilot study, we demonstrate the framework using eight epidemiologically diverse *Listeria monocytogenes* strains, with strain differentiation and trait-based grouping as proof-of-principle tasks. MicroICS extracted over 2,700 quantitative structural features from Syto 9-labelled biofilm images. An optimised random forest model achieved human baseline-level accuracy in strain classification on previously unseen images, including biofilms experimentally perturbed by food extracts. BiofilmQ-derived features incorporated into the classification module yielded higher accuracy with fewer features than either tool alone, confirming the framework’s extensibility. Extending the framework to clonal complex-based clinical grouping demonstrates utility beyond strain identity. MicroICS is applicable to any organism or condition for which suitable 3D biofilm images can be obtained.

## Introduction

Microbial biofilms are ubiquitous and represent the predominant life form of microbes in virtually all environments^1^. The importance of biofilms in various areas of human and animal health is increasingly recognised. Their presence causes significant detrimental effects, particularly in relation to biocorrosion, human health and agriculture, resulting in an estimated global cost of nearly $4 trillion per year^2,3^.

Biofilms are highly structured microbial communities that form three-dimensional (3D) structures in which microbes adapt according to the microenvironments they occupy within the biofilm^4^. The best-known changes associated with the biofilm lifestyle are increased tolerance to antimicrobial agents and the ability of microbes to survive in extreme environmental conditions^5^. The nature of a biofilm depends on the bacterial species and strains involved, as well as environmental conditions^6,7^. Differences in biological and chemical composition further drive adaptive spatial structures optimised for survival under specific conditions^8^.

As 80% of bacterial infections involve biofilms, which are often recalcitrant to treatment, quantifying strain-level differences in biofilm formation is critical for understanding survival, pathogenicity, resistance, and clinical outcomes^9^. Thicker biofilms and increased extracellular matrix production are well-established drivers of antibiotic tolerance and immune evasion^10,11^. While strain-dependent variations in biofilm structure contribute to differences in persistence and treatment failure, often leading to chronic and recurrent infections^12^, a clearer linkage between biofilm properties and functional outcomes is needed to elucidate the multifactorial nature of biofilm resilience, which is driven by genetic, metabolic, and structural factors^13,14^. Identifying such vulnerabilities can support the development of targeted therapies, including biofilm-disrupting agents and optimised antimicrobial regimens^15^.

Microscopy is the only technique that allows detailed analysis of the 3D structure of biofilms in situ at the single-cell level. Most knowledge about biofilm structure is derived from scanning electron microscopy and confocal fluorescence microscopy^16^. They offer high image quality, high resolution and can preserve the structure, enabling quantitative 3D analysis of biofilms. However, the electron scattering-based methods require additional laborious and time-consuming sample preparation and the use of expensive equipment. In addition, their inherent inability to track the biofilm formation in real time is very limiting to the facilitation of new discoveries and understanding their causal connections, for which fluorescent microscopies are more suitable. Confocal fluorescence microscopy enables the acquisition of large 3D image datasets under native conditions, generating rich structural information across multiple spatial scales. However, the complexity and high dimensionality of these 3D biofilm images present significant challenges for quantitative analysis and biological interpretation. Tools such as COMSTAT, DAIME, BIAM, and BiofilmQ have made important contributions to automated feature extraction and structural quantification of 3D confocal biofilm datasets, and remain widely used in the field^17–20^. However, these tools were designed primarily for structural quantification and visualisation, and do not offer integrated frameworks for predictive image classification or hypothesis-driven downstream feature analysis, which are increasingly necessary as imaging datasets expand in scale and biological complexity.

Machine learning (ML) has emerged as a powerful approach to uncover patterns and make predictions in complex biological systems, including biofilm research, where it has been used to model biofilm formation, identify genes involved in this process, and classify structural phenotypes^21,22^. By leveraging supervised learning methods, ML can analyse the large numbers of quantitative features extractable from 3D biofilm images and provide a robust means to classify bacterial strains and predict phenotypic behaviours^23^. Ensemble methods such as random forests are particularly well suited to high-dimensional biological datasets, offering strong predictive performance, resistance to overfitting, and interpretable feature importance scores that can be related to biological meaning. Automated machine learning (AutoML) frameworks further streamline this process by automating model selection, hyperparameter optimisation, and validation, reducing the need for specialist expertise and minimising human bias in model development^24,25^.

A critical aspect of ML in biofilm research is the interpretability of models, which is essential for biological validation and hypothesis generation. Feature ranking methods, such as SHapley Additive exPlanations (SHAP) and Local Interpretable Model-agnostic Explanations (LIME), provide insights into the contributions of individual features to the predictions made by ML models^23^. These methods can be used, for example, to determine which structural features of biofilms are most strongly associated with the survival and response to environmental stimuli of certain bacterial strains. This knowledge can then guide experimental designs and interventions^23^.

In the context of biofilm image classification, ML approaches have been successfully applied to biofilm image analysis in various settings, such as classifying bacterial species from optical coherence tomography images and classifying two-dimensional images of acidophilic biofilms on minerals^26,27^. These studies demonstrate the potential of ML for biofilm phenotyping, but each is specific to a particular imaging modality, organism, or predefined classification scheme, and none has been adapted to 3D confocal fluorescence microscopy.

*Listeria monocytogenes*, a foodborne pathogen with a high mortality rate, serves as the model system in this study^28^. Its biofilms present a persisting challenge in the food industry, often causing contamination, food recalls, and outbreaks^29^. This pathogen has a clonal population structure, meaning that the genetic properties of strains are highly similar and show limited diversity. Based on their epidemiological association, *L. monocytogenes* clonal complexes (CCs) are differentiated into: (i) hypervirulent clones significantly associated with clinical origin, (ii) hypovirulent clones significantly associated with food processing environments and persistence, and (iii) intermediate clones^30^. Unlike hypervirulent CCs, the hypovirulent strains are thought to maintain contamination reservoirs despite lower clinical association. However, *L. monocytogenes* CCs do not coincide with biofilm-forming capacity, nor are there genes uniquely associated with this trait. Consequently, despite underlying genetic similarity, biofilm formation appears to be a complex phenotype that must be measured independently^31,32^. Based on biochemical and microscopic examinations, *L. monocytogenes* strains differ considerably in their biofilm structure; however, the biological significance of these differences remains unknown^31,32^.

**Fig. 1:**
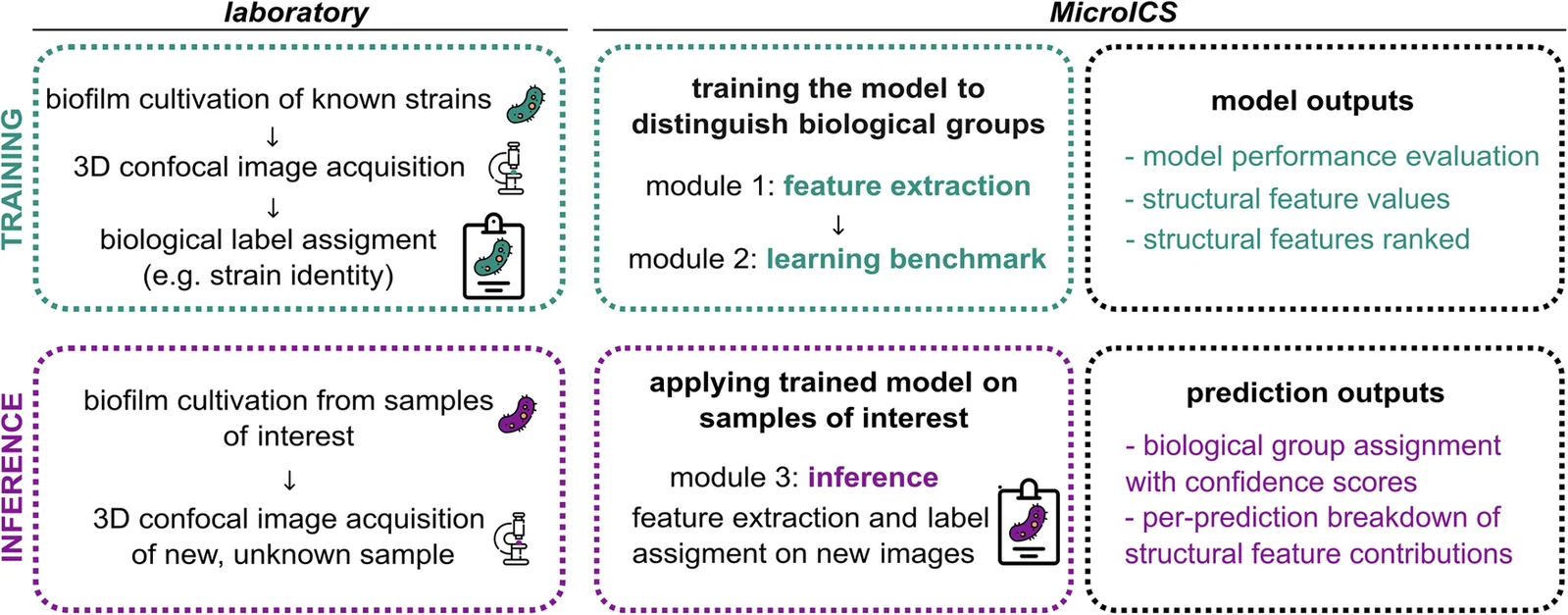
Overview of the MicroICS framework. The workflow begins with the acquisition of 3D confocal images of biofilms grown under conditions relevant to the biological question of interest. During the training phase, structural features are automatically extracted from these images and used to compare and select an ML classification model trained to distinguish the biological groups of interest. Once trained, the model can be applied in the inference phase to new, unseen images acquired under the same microscopy conditions, predicting the biological property of interest for each image. Each module can be run independently, enabling integration with existing image analysis tools.

To address the absence of an integrated framework for predictive classification and biological interpretation of 3D biofilm data from confocal microscopy images, we developed the Microbial Image Classification Suite (MicroICS) (Fig. 1), an open-source ML framework designed to extract meaningful biological information from 3D biofilm images. MicroICS consists of three independent yet interoperable modules: feature extraction from 3D confocal images, ML-based classification for predictive phenotyping, and an inference module for applying trained models to new, unseen images (Fig. 1). Each module can be run independently, and the classification module accepts externally generated features, enabling integration with existing quantitative image analysis tools. The underlying principle is that if a biological property of interest—such as strain identity, phenotypic state, or response to environmental conditions—is reflected in the spatial organisation of a biofilm, then ML can detect that signal from images without requiring manual definition.

As a pilot study, we developed and benchmarked MicroICS using *L. monocytogenes* as a model system. For benchmarking, we selected strain identity prediction—a deliberately demanding test, as biofilm structure varies substantially across biological replicates and is highly sensitive to environmental perturbation. This task was chosen not because image-based strain identification is the primary application, but because it probes the finest level of structural discrimination the framework can resolve; genomic methods remain far more robust for definitive strain identification. To benchmark MicroICS feature extraction against an established tool, BiofilmQ-derived features were incorporated into the classification module, confirming that externally generated features integrate seamlessly and improve classification efficiency. We then applied MicroICS to other biologically motivated questions, including the structural effects of food extracts on biofilm formation, where the framework revealed novel insights and also exposed interpretive limitations worth considering.

Importantly, the *L. monocytogenes* results presented here are not intended as a deployable classifier for other laboratories—microscopy setups, imaging conditions, and biological contexts will differ. Instead, they serve as a documented example of how the framework can be built, verified, and interpreted for a specific biological question. The framework applies to any organism or condition for which reliably labelled 3D biofilm images with sufficient biological replication are available, and can address any biological question that can be represented as a classification task.

## Results

To demonstrate its capabilities, we developed and benchmarked the framework using a pilot dataset of Syto-9-stained biofilms from eight epidemiologically and genetically diverse *L. monocytogenes* strains as a model system (Table S1 and Figs.S1 and S2).

Strain identity prediction was selected as the primary benchmark task because it represents the finest level of structural discrimination the framework can resolve—distinguishing strains whose biofilm structures differ subtly, vary within a single strain depending on growth conditions, and cannot be reliably classified by visual inspection alone. Visual inspection of the dataset confirmed its suitability as a pilot system for algorithm development and benchmarking: strain ATCC 19115 formed dense structures concentrated in the bottom layers with numerous surface microcolonies, while strain L1764 produced biofilms distributed uniformly throughout the volume (Fig. S2). The remaining six strains exhibited intermediate characteristics that were often difficult to distinguish due to variability between repetitions (Fig. S2), adding a realistic degree of complexity to the benchmark and ensuring that the results could be confirmed by human experts if required. Beyond strain identity, the framework was also applied to a biologically motivated secondary task – grouping strains by CC as a proxy for clinical association—demonstrating that the same pipeline can be directed towards different biological questions without modification.

Results are presented in three sections, each corresponding to an interoperable module of the MicroICS framework (Fig. 1). Feature extraction, the first step after image acquisition, is not presented as a standalone result; instead, features are introduced within the context of the analyses for which they were informative. MicroICS offers a data-driven feature extraction module that operates without user-defined parameters or segmentation thresholds. The classification module, however, accepts any externally generated feature set, as demonstrated here by the incorporation of BiofilmQ-derived features alongside MicroICS features. Throughout the results, it is clearly stated whether findings refer to MicroICS features alone or to the combined MicroICS and BiofilmQ feature space. It should be noted that specific feature sets may change across software versions; the ML framework is designed to operate on whichever features the user provides.

### Classification performance and comparison of ML models for strain identification from biofilm features

**Fig. 2:**
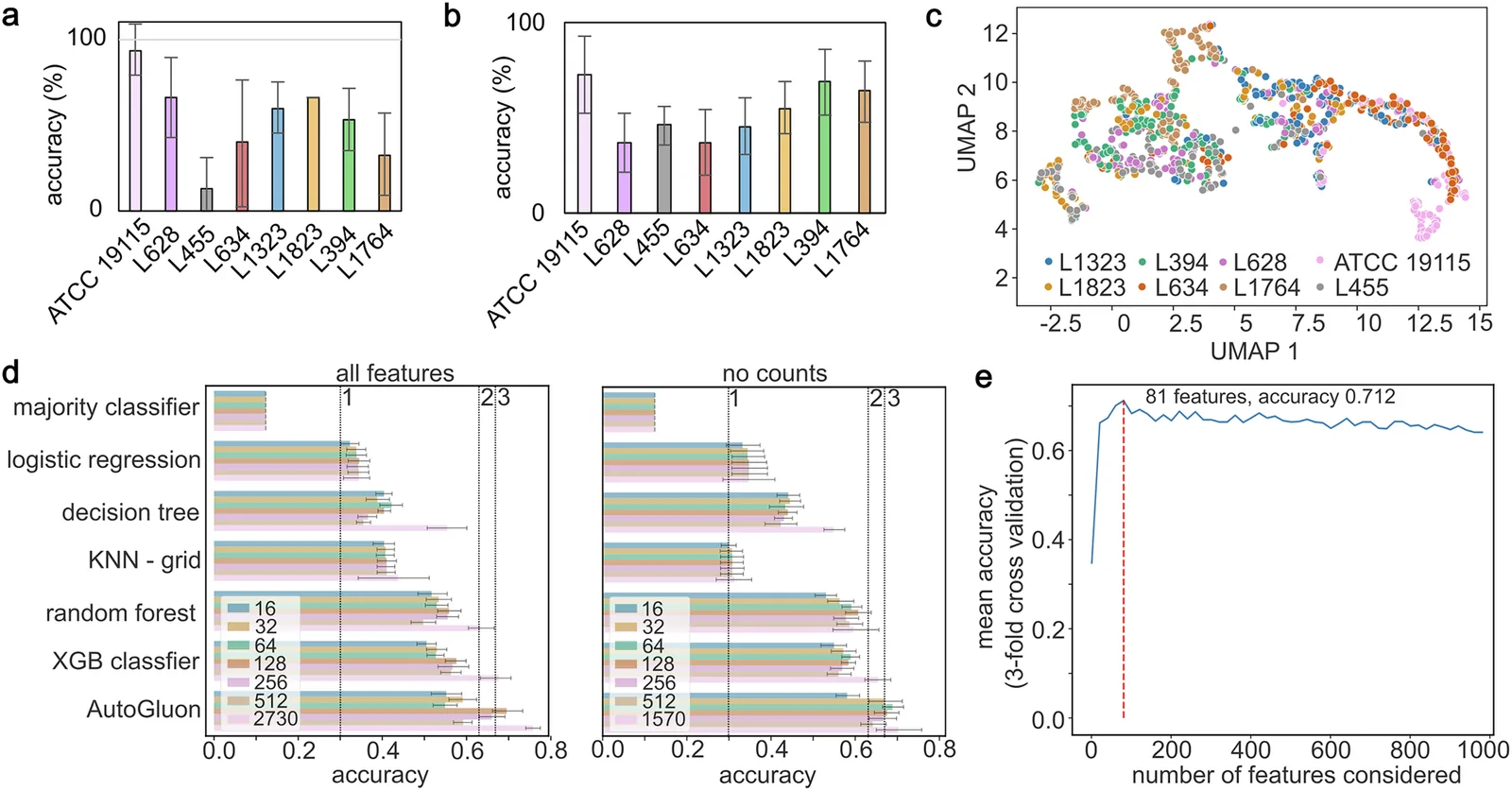
Classification performance and algorithm comparison for identifying *L. monocytogenes* strains based on images. **a** Average visual classification accuracy achieved by three laboratory members inspecting selected images of each strain. **b** Average classification accuracy per strain achieved using the random forest algorithm. **c** UMAP visualisation showing the clustering of strains in two-dimensional space based on all computed features, prior to classifier training. **d** Classifier performance for strain identity prediction. Performance using all computed features as input (all features), with dimensions up to 512 representing feature projections and 2700 representing the full original feature set, and classification performance excluding the feature “count” using the same dimension projections for comparison (no counts). The human baseline is marked by the dotted line for each individual laboratory member. **e** Feature ablation results indicate that only a subset of features is necessary for high classification accuracy.

To establish a human performance baseline, three laboratory members independently classified a masked subset of 3D confocal images from each strain after familiarisation with labelled reference images (Table S3). Accuracy was defined as the proportion of correctly identified strains out of the total number of masked images assessed. The overall classification accuracies achieved were 62.5%, 30%, and 67.5%, respectively. The modest overall accuracy and considerable variation between individuals indicate that visual classification of *L. monocytogenes* strains from 3D biofilm images is genuinely difficult and insufficiently reliable for systematic use. The most accurately classified strain was strain ATCC 19115, followed by strains L628, L1823 and L1323; the most misclassified strains were L1764, L455 and L634 (Fig. 2a). The accuracy also showed notable variability in standard deviation across strains, suggesting that some biofilms possess features more easily distinguishable by the human eye, while others remain visually indistinguishable. However, these differences may inflate the overall accuracy metric.

For ML benchmarking, accuracy was defined as the mean proportion of correctly classified held-out images across all folds and repetitions of the stratified cross-validation procedure. All ML classifiers substantially outperformed the majority classifier (accuracy 12%), confirming that the approximately 2700 quantitative features extracted by MicroICS effectively capture strain-discriminating structural information (Fig. 2d). Among the classifiers evaluated, XGBoost (XGB), random forest, and AutoGluon all achieved accuracies above 50%, with random forest and AutoGluon matching or exceeding the best human baseline of 67.5%—a result that is particularly notable given that the minimum human accuracy was only 30%, reflecting the inherent difficulty of the task.

Among the classifiers evaluated, tree ensemble methods (random forest and XGB) showed remarkably consistent performance across both the full feature set (~2700 dimensions) and the SVD-reduced feature set (512 dimensions). SVD projects the dataset onto a new coordinate system to reduce dimensionality, which shortens processing times but can introduce noise and cause some performance degradation. Importantly, because the resulting feature groups differ depending on the target number of dimensions, results obtained at different SVD settings are not directly comparable in terms of which biological features drive classification. AutoGluon achieved the highest overall accuracy, particularly on the SVD-reduced dataset (256 dimensions), where both computational feasibility and classification performance were optimised. However, AutoGluon’s default configuration did not identify dimensionality reduction as beneficial, requiring manual inclusion of SVD as a pre-processing step. Although AutoGluon achieved the highest predictive accuracy, its substantial computational overhead made it impractical for routine use on standard hardware. Random forest was therefore selected as the recommended classifier, offering a favourable balance of predictive performance, interpretability, memory usage, and runtime without requiring parallelisation or specialist computing resources.

Random forest accuracy closely matched the best human baseline (Fig. 2d). Per-strain comparison showed that random forest outperformed visual assessment for strains L455 (ML: 46% vs visual: 13%) and L1764 (ML: 64% vs visual: 33%), while visual assessment was higher for L628 (ML: 37% vs visual: 66%), likely reflecting the more visually prominent structural characteristics of that strain (Fig. 2b). Overall, per-strain classification improved compared to visual assessment, as indicated by reduced variability in accuracy across strains (Fig. 2a, b), suggesting that the pipeline captures subtle 3D structural information present in the images but not accessible through visual inspection alone. The count-based features contributed meaningfully to classification: their exclusion consistently reduced accuracy across all classifiers, consistent with bacterial abundance being part of the structural fingerprint that distinguishes strains, even though these features depend on intensity thresholds and their absolute values should be interpreted with reference to the original visual segmentation.

To assess robustness, we examined whether classifier rankings remained stable across repeated cross-validation folds^33,34^. The relative ordering of classifiers was consistent across all five repeats of the 3-fold cross-validation, regardless of whether the full or reduced feature set was used, and whether count-based features were included or excluded. This stability indicates that performance differences between classifiers reflect genuine and reproducible characteristics of the methods rather than artefacts of any particular data split, and provides confidence that the conclusions are reliable across the range of biological and technical variability present in the dataset.

The added value of the ML approach does not stem from generating fundamentally different descriptors than those obtainable with established quantitative image analysis tools, but from the multivariate organisation of these descriptors: identifying which combinations of features are genuinely predictive for a given biological question, separating strain-associated signals from technical confounders such as experimental date, and enabling inference on unseen images, including those with experimentally perturbed structures. Classification performance inherently depends on the features supplied to the model, whether automatically extracted by MicroICS or incorporated from external tools, and the resulting biological conclusions are shaped accordingly. To further demonstrate this, classification performance using BiofilmQ-derived features and the combined MicroICS and BiofilmQ feature set is presented below the following feature analysis section, along with the corresponding feature rankings, which provide the necessary context for interpreting the classification outcomes reported above.

### Feature analysis and interpretation for strain identity classification

Before applying supervised classification, the discriminative potential of the MicroICS feature space was first explored using UMAP—an approach that reduces thousands of structural measurements to a two-dimensional map, allowing visual assessment of whether biofilms from different strains occupy distinct regions of the feature space without using strain identity as input. UMAP was applied to features from individual biological replicates (Fig. S3) and to the combined dataset (Fig. 2c). In the UMAP projection of individual replicates, ATCC 19115 and L1764 were clearly separated from other strains, consistent with their visually distinct morphologies, while L1823 and L455 showed partial separation. The remaining strains formed largely overlapping clusters, suggesting greater structural similarity within this group and indicating that unsupervised feature organisation alone is insufficient for reliable strain discrimination across the full panel.

Supervised ML thus substantially improved strain discrimination (Fig. 2c, d). Ablation analysis of the full feature space—comprising more than 2700 descriptors spanning morphological, textural and intensity-based descriptor families (Table S4)—identified a compact subset of 81 image-derived features sufficient for maximum classification accuracy across eight *L. monocytogenes* strains (Fig. 2e and Table S5). These features primarily capture morphological (GPT volume), intensity-based, texture (fractal dimension, void fraction), and grey level co-occurrence matrix (GLCM) correlation- based features of biofilm structure. None were computed over entire images; all were derived from layer calculations (Table S5). The reduction from 2700 to 81 features without loss of accuracy indicates that the model does not depend on a long tail of weak predictors and that strain-discriminating information is concentrated in a compact, interpretable descriptor set.

To eliminate potential batch effects, we removed features shared between batch (experimental date) and strain identity tasks—such as maximum and minimum fractal dimension and intensity standard deviation—retaining only strain-specific (identity) predictors (Table S5). These two high-ranking features aggregated by extremes overlapped between strain and date, but their quantiles (90^th^ and 10^th^ percentiles) did not; while minimum and maximum focus on the extreme edges of the dataset, percentiles usually give a more reliable overview of where the data points are concentrated, especially for biological data with large variability or outliers. Among the 81 most important features was also the standard deviation of minimum intensity values per layer, indicating that the lowest possible value for signal dispersion is also important for discriminating between strains.

As ML classification may not be intuitive for biologists, a statistical analysis of the key MicroICS-derived features is provided (Fig. S4). This demonstrates that each feature captures distinct biological aspects, collectively enabling the complex, multidimensional classification achieved by the ensemble model. The comparison of the key features using MANOVA was significant (unexplained variance rate: Wilks’ lambda < 0.001, *p* < 0.001). The subsequent univariate ANOVA revealed a highly significant strain effect for all features (*F*(7, 948) > 24.26, *p* < 0.001). The results of the Games-Howell pairwise comparisons are presented in Fig. S4. These statistical results confirm that the top features collectively capture strain-specific biofilm signatures that are too complex for individual variables alone.

Cross-correlation analysis of 81 MicroICS-derived features (Figs. S5 and S6) identified 20 least-correlated descriptors (|*r*| < 0.3, pale heatmap) that capture orthogonal biofilm aspects (intensity thresholds, geometry) and provide complementary classification information. In contrast, the 20 most-correlated features (|*r*| > 0.8, red/blue blocks) revealed redundant families differing only by aggregation choices (intensity cutoffs, normalisation), making them ideal for consolidation during feature selection to streamline the ML pipeline and reduce multicollinearity.

### Classification performance and feature analysis using externally generated features: BiofilmQ as a complementary source of features

To evaluate the extensibility of the MicroICS framework, classification performance was assessed using features generated by BiofilmQ alongside MicroICS-derived features. The two approaches are complementary by design: BiofilmQ generates biologically interpretable 3D object-based descriptors that require user-defined segmentation thresholds, while MicroICS produces data-driven image statistics at the layer level without manual parameter setting.

Classification using BiofilmQ features alone achieved an accuracy of 0.98 with 181 features. The combined MicroICS and BiofilmQ feature set—comprising 3040 features in total—achieved a classification accuracy exceeding that of MicroICS alone using only 81 features, matching the feature count of MicroICS alone while requiring fewer features than BiofilmQ alone to reach equivalent accuracy (Fig. S7 and Table S5). Among the top-ranked features in the combined set, both BiofilmQ-derived 3D object metrics and MicroICS intensity statistics were represented, confirming that neither tool alone captures the full discriminative signal available in the data.

Batch effect analysis of the combined feature set revealed that the overlap with acquisition date was considerably more pronounced among top-ranked BiofilmQ features—predominantly volume and surface variability metrics—suggesting these may reflect imaging conditions as much as biological structure. Such features should be interpreted with caution, and their recurrence across experimental batches should be monitored when applying the framework to new datasets.

The model selected and trained during classification benchmarking—in this case, random forest—was subsequently deployed for inference on unseen biofilm images, as described in the following section. The current inference module operates on MicroICS-derived features only, as feature extraction and inference are integrated into a single computational step; support for external feature sets is under development and will be incorporated in a future release.

### Strain identity inference on unseen *L. monocytogenes* biofilm images with experimentally altered biofilm structures

The performance of the inference module was evaluated on biofilm images of strains L628 and L1764 grown on melon extract, steak tartare extract, and milk residues. Biofilm growth and imaging conditions matched those of the original training set, with no interference from the extracts during imaging.

We optimised the inference module using 5-fold stratified sampling on control images of two selected training strains, captured on dates different from those included in the training. As the true strain identities for these images were known, this allowed precise performance evaluation and guided model optimisation beyond parameter tuning. Inference accuracy was defined as the proportion of individual images correctly assigned to their true strain label, with group-level predictions determined by a majority vote across replicate images. Classification accuracies of 0.90 (SD 0.03) for L1764 and 0.63 (SD 0.14) for L628 were achieved, with mean prediction confidences of 0.71 and 0.39, respectively. These results are considered acceptable, given that the strains represent tasks of different complexity, and with adequate experimental replication, reliable conclusions can still be drawn.

**Fig. 3:**
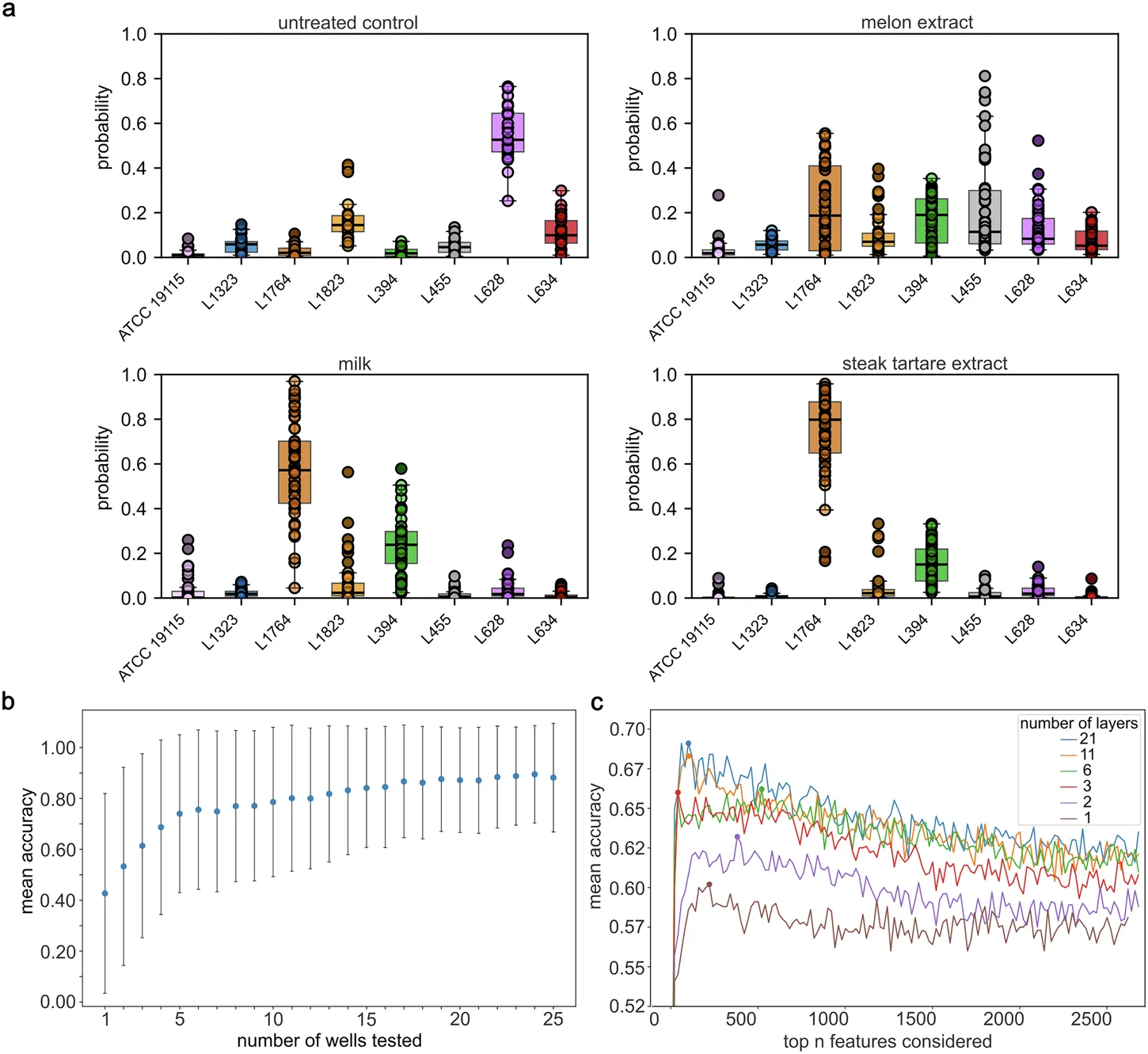
Inference module performance for strain identification and data optimisation. **a** Box and whisker plot analysis of L628 probability distributions and complete class profiles across food extract treatments. Box plots show the distribution of predicted probabilities across eight classes (ATCC 19115, L628, L455, L634, L1323, L1823, L394, and L1764; each strain represents a classification class) for each treatment group, with individual measurements shown as coloured dots. Food extract treatments shifted L628 biofilms toward L1764-like morphology, with steak tartare and milk showing the strongest resemblance, followed by melon. **b**, **c** Random forest performance with reduced input data for strain differentiation. The performance of the random forest algorithm is evaluated as input data is progressively reduced in two ways: **b** through wet-lab experimental optimisation using the developed pipeline, and **c** by minimising training datasets for strain classification.

Food extract treatments (melon, steak tartare, and milk) caused L628 biofilms to shift toward L1764-like morphology. The probability of L1764-like morphology was 0.22 (SD 0.18) for melon, 0.58 (SD 0.22) for milk, and 0.74 (SD 0.19) for steak tartare (Fig. 3a). Domain experts confirmed that biofilms treated with milk and steak tartare resembled the scattered aggregate growth of L1764, while L628 formed more bottom-concentrated biofilms with microcolonies and floating aggregates (Fig. S8). However, melon-treated biofilms were classified as L1764-like by majority vote, but with low confidence (Table S6 and Fig. 3a). This demonstrates the model’s nuance: it selects the closest matching strain while indicating limited structural similarity through a low probability. Visually, melon-treated L628 biofilms differed notably from all strains in the training set.

Feature rankings from training identified key discriminative features, which remained the top predictors under food treatments according to average SHAP values. Notably, the same structural descriptors—like fractal dimension and void volume—drove both the original L628 classification and its shift towards L1764-like morphology (resulting in ‘class switching’), with class differences rooted in feature values (Figs. S9 and 10). Controls confirmed model robustness (L1764: 0.96; L628: 0.76; Fig. 3a), while L1764 itself showed no significant structural changes across nutrients (Table S6 and Fig. S9).

### Use of the developed pipeline to optimise the experimental design to distinguish between the strains

To determine the number of technical replicates required to maintain strain identity prediction accuracy, we pooled wells from three experiments and iteratively tested 1 to 25 wells, measuring classification accuracy at each step. Accuracy improved greatly as we increased the number of wells from 1 to 5, and then gradually increased to 17 wells before reaching a plateau (Fig. 3b). This indicates that the use of 17 wells (each of which was analysed at 5 different positions within the well) is sufficient to obtain high accuracy of classification of *L. monocytogenes* strains from 3D biofilm images.

We then tested whether reducing the number of layers in 3D biofilm images affected the classification accuracy by simulating images with fewer layers. Using images with all 21 layers resulted in the highest accuracy (Fig. 3c). The reduction to 11 layers resulted in an accuracy close to that of 21 layers but with a consistently lower trend, meaning that missing layers already cause some loss of useful information. Further reduction of layers resulted in a significant drop in accuracy, which is probably not acceptable for biofilm image classification (Fig. 3c). Several features could not be calculated with only one layer, degrading the quality of the input and thus leading to significantly poorer results. Regardless of the number of layers, fewer than 500 features explained the complexity of the system, with this number generally increasing as the number of layers decreased. Overall, reducing the number of layers in a 3D image from 21 to 11 layers may be a reasonable compromise between accuracy and imaging time, depending on the application.

### Classification of clinical and non-clinical *L. monocytogenes* CCs from 3D biofilm features

Strains were grouped as clinically associated (CC1, CC4) or non-clinically associated (CC9, CC121) based on the CC, creating a binary classification problem with four strains per class. Random forest classification performed adequately for this task (Fig. S11), with 61 features sufficient to capture system complexity (Table S7). Count-ensemble features dominated the top predictors, and abundance-related descriptors were more important for clinical association classification than for strain identity classification, where textural features predominated.

**Fig. 4:**
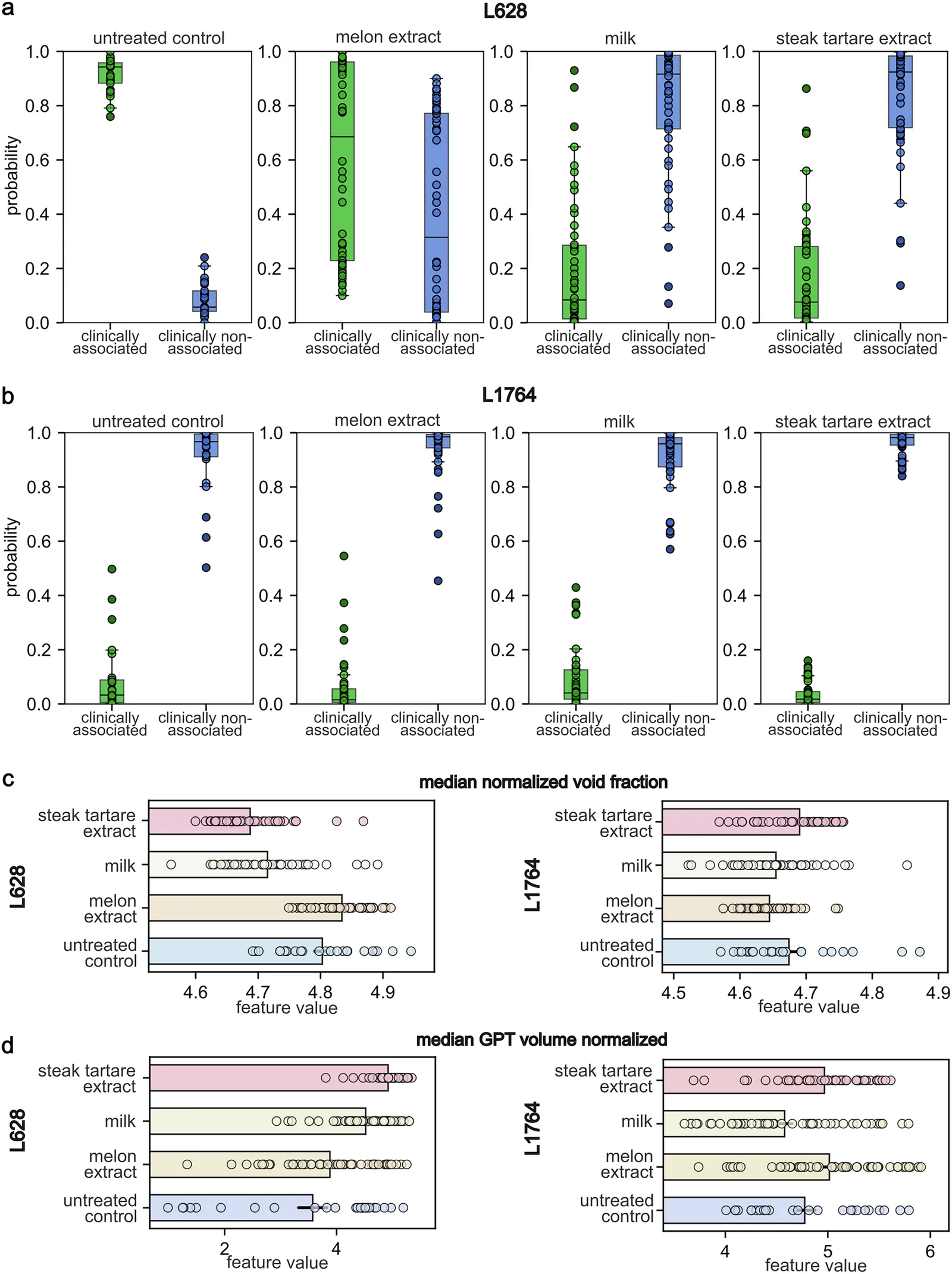
Inference module performance for linking clinical associations assigned by CC to biofilm structure features. **a** Food extract treatment shifts the clinically associated strain L628 towards structural features characteristic of non-clinically associated strains. Box-and-whisker plots show clinical association probabilities (CC-based classification: clinically associated vs non-associated) for strains L628 and L1764 across treatments (untreated control, melon, milk, steak tartare extracts). Coloured dots represent individual predictions. **b**, **c** Bar plots display the values of two selected key structural features (void fraction and GPT volume) for clinical association classification across food extract treatments. These plots illustrate how feature values contribute to class switching. Void fraction emerged as important for inference, while GPT volume remained a strong predictor from training.

To test whether biofilm structural changes could shift strains toward clinically associated or non-clinically associated biofilm structural features, we reused images from the identity task. Food extract treatments induced robust class switching in strain L628 under milk and steak tartare conditions, shifting its 3D structure toward the clinically non-associated class. This does not imply reduced clinical relevance for L628—separate validation is required—but it again demonstrates detectable structural changes. Strain L1764 remained stable across perturbations (Fig. 4). Average SHAP analysis on inference data revealed distinct shifts from training feature rankings: from count-based dominance to predominance of intensity features, void fraction, and consistent GPT volume importance in the clinical association classifier (Fig. S12). In addition, these shifts directly corresponded to observed class switching (Figs.S4 and S12).

## Discussion

As the importance of microbial biofilms to human health is increasingly recognised, the need to characterise their3D structures also grows. Elucidating this structure is crucial for understanding their biological complexity, functions, and responses to environmental or therapeutic interventions. Here, *L. monocytogenes* was used as a model system—an exemplary pathogen whose biofilms enable persistence in food environments, leading to recurrent contamination, recalls, and outbreaks when undetected^29^. Reflecting their importance, previous studies have characterised the diverse 3D structures of these biofilms, ranging from monolayers and unstructured multilayers to knitted chain networks and honeycomb-like patterns, which vary by strain, growth conditions, and preparation methods^31,35,36^. The structural diversity observed across the eight strains in this study is broadly consistent with previously reported strain-dependent variation in *Listeria* biofilm structure. Notably, the apparent morphology is also strongly influenced by experimental setup—systematic confocal microscopy-based analyses under static conditions have described different structural arrangements from those seen in flow-based systems—further limiting direct cross-study comparison. Direct comparison is also constrained by differences in sample preparation, imaging modality, and fluorescent labelling approaches—methodological variables known to influence apparent biofilm morphology. The structures observed here are therefore interpreted as part of an established continuum of *Listeria* biofilm structure whose exact appearance depends on experimental context, and not as new morphotypes. Visual classification of biofilm images has nevertheless proved insufficiently reliable and reproducible across the intra- and inter-experimental variability inherent in biofilm assays—a limitation confirmed by considerable variation in classification accuracy among laboratory members in this study. Conventional quantitative approaches applied to *Listeria* and other biofilm-forming species have used biovolume and thickness as primary structural descriptors; while informative, these measures do not capture the full complexity of three-dimensional biofilm structure. COMSTAT remains foundational for quantitative 3D morphometry, while BiofilmQ substantially expands spatially resolved quantification, visualisation, and analysis of biofilm-internal properties; both are well-established tools for structural characterisation. The specific contribution of MicroICS is to complement these tools by adding a downstream predictive layer: integrating structural feature extraction with supervised classifier comparison, feature prioritisation, SHAP-based attribution, and inference on unseen images within a single reproducible workflow—transforming a descriptive inventory of structural measurements into a predictive and interpretable phenotyping framework that can be applied to different biological questions without modification.

The modular architecture of MicroICS also enables externally generated features to be incorporated directly into the classification pipeline, as shown by integrating BiofilmQ-derived features with MicroICS features. The combined feature set achieved higher classification accuracy with fewer features than either tool alone, confirming that the two approaches capture complementary structural information. However, BiofilmQ features—mainly volume and surface variability metrics—showed substantial overlap with acquisition date, indicating sensitivity to imaging batch effects, whereas MicroICS intensity-based features showed minimal overlap, suggesting greater robustness to technical variation. These findings demonstrate both the practical extensibility of the framework and an important consideration for users: when incorporating external features, overlap with technical variables such as acquisition batch should be assessed and accounted for during feature selection.

Strain identity was selected as the primary benchmark task because it provides a biologically meaningful and verifiable test case: ground truth is unambiguous, performance can be evaluated against human observers, and the dataset can be constructed systematically from available epidemiological and genetic metadata. This approach also yields a reusable benchmark dataset of diverse, biomarker-characterised strains with paired images and biological annotations—a resource that may support reproducibility and benchmarking in future studies. Beyond strain identity, the framework was extended to clinical association through CC grouping, demonstrating its applicability to different biological questions using the same pipeline.

Classifiers evaluated included GridSearchCV-optimised models, random forest, logistic regression, XGB, and the AutoML framework, AutoGluon. Ensemble tree methods were consistently robust across both full and reduced feature sets, confirming their suitability for high-dimensional biological data. Although AutoGluon achieved the highest overall accuracy, its default configuration did not identify dimensionality reduction as beneficial without manual intervention—an important limitation of heuristic-based AutoML frameworks when applied to data with unusual structural characteristics. The relative ranking of classifiers remained stable regardless of feature subset, demonstrating methodological reliability across varying data conditions.

The strain discrimination achieved by the classifiers was supported by the breadth of the feature space: the MicroICS framework extracted approximately 2700 quantitative biofilm features from 3D images, covering structural, textural, and signal intensity aspects using primarily literature-derived metrics. All classifiers tested substantially outperformed the majority classifier, confirming that this feature space effectively captures strain-discriminating structural information. The considerable variation in human classifier accuracy highlights that *L. monocytogenes* biofilm structures do not differ between strains in ways that are readily apparent to the trained eye. Random forest classification achieved overall accuracy comparable to the human baseline, with less variation in per-strain accuracy than visual classification, where large outliers tended to inflate the average. The model substantially outperformed visual assessment for strains that humans found most difficult—notably L455 and L1764—suggesting that the extracted features encode biologically meaningful, strain-specific structural information not accessible through visual inspection alone. Although random forest was not the highest-performing algorithm overall, it offered sufficient accuracy to distinguish subtle structural differences while remaining feasible on standard hardware, making it the practical choice for routine use. The exclusion of count-based features consistently reduced classifier accuracy, consistent with bacterial abundance contributing to the structural fingerprint that distinguishes strains, even though these features depend on intensity thresholds and should be interpreted with reference to the original visual segmentation.

The MicroICS calculated features distinguishing the eight *L. monocytogenes* strains were predominantly layer-based, emphasising structural gradients along the vertical axis rather than global image properties. This aligns with the spatial organisation of biofilm development: initial attachment and early growth occur at the substratum, producing the densest and most structurally consistent signal, while upper-layer occupancy—reflecting loosely bound or late-stage biofilm—varies between strains and thus provides greater discriminative information. This vertical heterogeneity is a recognised biological property of mature biofilms, where oxygen and nutrient gradients drive differential growth along the *z*-axis, and it can be hypothesised that strain-specific differences in surface attachment or matrix production may manifest most clearly in these upper-layer dynamics. The importance of layer-specific information is also directly demonstrated by the rapid decrease in classification accuracy when vertical resolution is reduced. Maintaining classification performance also required a substantial number of technical replicates per strain, reflecting high natural variability even under controlled conditions and without perturbation of 3D structure by biofilm washing. Inter-replicate variation, particularly from independent biological inocula, was considerable. Sufficient experimental replication, therefore, remains essential—the classification framework cannot compensate for inadequate sampling of biological variability, but is well suited to extracting discriminative signals from large, high-dimensional datasets where human assessment is impractical.

To maintain accuracy in inference across experimental batches, control images were used for data augmentation, with replicates divided between training and inference to introduce beneficial variability and provide checks against batch effects. The modestly lower classification accuracy for L628 compared to L1764 reflects inherent strain-specific challenges, likely attributable to its subtler and more variable biofilm phenotype. Majority voting across replicate images effectively mitigated image-level noise, increasing confidence in group-level predictions. Strain-specific classification performance should therefore always be reported and interpreted alongside overall accuracy in any application context.

Biofilms grown on food-extract-coated surfaces tested the framework’s sensitivity to biologically relevant structural perturbations. The two strains responded differently: L1764 maintained a stable structural phenotype across all matrices, while L628 exhibited pronounced structural shifts, with the degree varying by matrix. Milk and steak tartare induced the strongest transition of L628 biofilms towards an L1764-like dispersed phenotype, while melon produced a more atypical morphology that the model identified as phenotypic novelty through low confidence scores, rather than forcing a confident but potentially erroneous assignment—a behaviour desirable in exploratory biological applications. Fractal dimension and void fraction were the key structural descriptors driving both the original strain discrimination and the treatment-induced class switching. Fractal dimension reflects the geometric complexity and space-filling properties of biofilm structure, with higher values indicating more irregular or aggregated structures and lower values indicating smoother, more uniformly distributed organisation; void fraction denotes the proportion of unoccupied volume, distinguishing dense from sparse communities. Consistent with their contrasting morphologies, L628 exhibited higher fractal dimension and lower void fraction than L1764, and the shift of L628 toward lower fractal dimension and higher void fraction under milk and steak tartare treatment is consistent with a transition from aggregate-based to dispersed growth—a structural reorganisation whose functional implications for surface persistence and resistance to removal warrant further investigation. These results suggest that expanding training strain diversity and growth conditions will be an important next step towards broader applicability of the framework.

The distinction between strain identity prediction and CC-based clinical grouping illustrates how different biological questions are reflected in different aspects of biofilm structure. Strain identity prediction favoured textural complexity, whereas clinical grouping prioritised abundance-related features—a distinction consistent with the known epidemiology of *L. monocytogenes* niche adaptation. Hypervirulent clones are typically associated with clinical and host contexts, whereas hypovirulent clones such as CC9 and CC121 are frequently linked to food-processing environments and persistence, where proficient biofilm formation is considered advantageous^30^. However, the relationship between persistence and biofilm formation is not uniform across studies, and a simple genotype-to-biofilm mapping has not been established. These results therefore suggest that 3D biofilm structure captures part of the ecological signal associated with CC background, rather than providing a direct readout of virulence potential. Interpretation of the abundance-related features driving this classification warrants additional caution: count-ensemble descriptors are derived from intensity thresholds that conflate object counts with signal strength and do not represent pure bacterial abundance in an absolute sense. Combined with the limited number of strains per class in the present study, these structural descriptors should be regarded as starting points for hypothesis formation rather than reliable indicators of clinical behaviour – potentially useful for comparative analysis and strain prioritisation, but requiring validation through genomic and functional approaches before any clinical inference can be drawn.

Task-specific feature priorities also apply to inference settings. Strain identity classification depends on stable, biologically ingrained structural fingerprints that remain consistent across training and inference datasets. In contrast, clinical association—a statistically defined grouping—shows more flexible feature dependence, with food-matrix treatments shifting the balance from abundance-based to intensity- and texture-based predictors as L628 biofilm structure reorganised from dense microcolonies to a dispersed structure. This sensitivity to treatment-driven structural reorganisation demonstrates that the framework captures biologically meaningful phenotypic transitions rather than artefactual classification shifts.

This pilot study on *L. monocytogenes* revealed substantial differences in structural plasticity between strains: L628 exhibited pronounced structural shifts under food treatments, while L1764 maintained phenotypic stability—a difference reflecting strain-specific environmental responsiveness relevant to understanding persistence and adaptation. Such plasticity must be interpreted with caution and should not be directly equated with task-specific classifications, particularly clinical association grouping. The structural predictors identified here should therefore be regarded as starting points for hypothesis formation rather than established biological markers. Although biofilm structure has long been qualitatively linked to function, systematically relating quantitative 3D structural descriptors to specific biological outcomes across strains and conditions remains challenging. MicroICS provides a scalable framework for generating such hypotheses from high-throughput imaging data, identifying which structural features co-vary with biologically meaningful groupings and indicating targeted experiments—such as genetic manipulation and biochemical characterisation—that can test whether these associations are causal.

This study intentionally focused on single-channel, static 3D structural images as a controlled proof-of-principle setting. Multi-channel fluorescence imaging—for example, simultaneously capturing biomass, matrix components, or viability—and time-series acquisition tracking biofilm development or response to treatment are likely to provide especially information-rich inputs for the type of workflow proposed here, and represent natural extensions of the framework. The modular architecture of MicroICS makes the incorporation of time-resolved or multi-channel feature extraction technically feasible and is a planned direction for future development. This study does not aim to cover all possible imaging modalities or use cases, but rather to establish a reproducible, accessible foundation that can be extended as imaging datasets grow in complexity.

The primary contribution of this work is practical: MicroICS offers researchers an accessible, open-source framework for extracting a high-dimensional structural phenotype space from 3D biofilm images and linking that space to relevant biological questions using supervised ML. These capabilities are not currently available within a single integrated workflow in existing biofilm image analysis tools, which provide robust structural quantification but leave downstream predictive analysis, feature ranking, and inference to the user. This pilot study demonstrates that meaningful phenotypic signals can be extracted from a limited but well-characterised panel of *L. monocytogenes* strains: strain-specific structural differences are detectable and quantifiable, ML can prioritise biologically interpretable feature combinations, and the framework generalises to experimentally perturbed conditions. Broader validation across more strains, species, laboratories, imaging conditions, and biological endpoints will be required before the approach can be recommended for diagnostic or screening applications; the present study, therefore, provides methodological groundwork and a reusable workflow rather than a deployable strain-identification or clinical-prediction tool.

## Methods

### Strains and cultivation

The bacterial panel comprised eight *L. monocytogenes* strains with known epidemiology (Table S1 and Fig. S1). It was designed to reflect the variability of *L. monocytogenes* across different genotypes (CCs) and environments. Seven strains were obtained from the strain collection of the National Veterinary Institute, Faculty of Veterinary Medicine, University of Ljubljana. Strain ATCC 19115 was also included in the panel. Based on the results of the previously performed multilocus sequence typing (MLST), the investigated strains were representative of hypervirulent/clinically associated (CC1, CC2, CC4 and CC6; *n* = 5), hypovirulent/food-associated (CC9 and CC121; *n* = 2) and intermediate clones (CC8; *n* = 1). The strains originated from different environments, including animals, food and humans. The genetic characteristics of the analysed strains and additional metadata on the strains are listed in Table S1. Strains were grown in Luria–Bertani broth (10 g/L tryptone, 5 g/L yeast extract, 10 g/L NaCl, pH 7.5) or on Luria–Bertani agar at 37 °C.

### Whole-genome sequencing and annotation of bacterial genomes, identification of genetic markers and genetic similarities

Genomic DNA was extracted with the DNeasy Blood & Tissue kit (Qiagen, Germany) according to the manufacturer’s instructions. Details regarding the sequencing technology are provided in Table S1. Raw reads were assembled using Shovill v1.0.9 (https://github.com/tseemann/shovill) with the—trim option and SPAdes v3.13.1 as the underlying assembler. Genome annotation was performed with Prokka v1.14.6^37^. Virulence genes were identified in the assembled genomes with VirulenceFinder 2.0 (https://cge.food.dtu.dk/services/VirulenceFinder/) using the default 90% identity and 60% coverage cut-offs. Genes of the LIPI-4 pathogenicity island were queried using BLASTn with thresholds of 90% identity and 60% coverage^38^. Premature stop codon mutations in the *inlA* gene leading to a truncated InlA were identified based on the alignment of amino acid (translated) sequences. Wall teichoic acid synthesis and biofilm-related proteins were identified using a custom database and stand-alone BLASTp and cut-off values of 60% coverage and 90% identity^39^. Each BLASTp hit was then manually verified using phmmer^40^. Dendrograms were generated via the hierarchical clustering routines in the SciPy cluster module (https://docs.scipy.org/doc/scipy/reference/cluster.html) using the median (a.k.a. “weighted”) linkage criterion. Clustering was performed on binary feature vectors encoding the presence or absence of each biomarker presented in Table S2 and Fig. S1.

### Preparation of biofilms and biofilm labelling for the training dataset

To prepare the biofilms, an overnight culture of each strain was harvested by centrifugation and the bacterial suspension was adjusted so that the absorbance at 600 nm was 0.1. The bacterial suspension was aliquoted (100 µl) into the wells of µCLEAR microtiter plates (Greiner BioOne, Austria) in 5–10 technical replicates. The bacteria were allowed to attach for 60 min at 37 °C, after which the bacterial suspension was aspirated to remove unattached planktonic cells and fresh growth medium was added. Initial attachment of cells to the surface was confirmed by brightfield microscopy prior to biofilm development. The biofilm was grown aerobically at 37 °C for 18-24 h. The biofilm-embedded bacteria were labelled with the DNA dye Syto 9 by carefully adding the dye to the growth medium above the biofilm to a final concentration of 3 µM. Syto 9 was chosen for its selective binding to DNA/RNA, high brightness, and lack of binding to media components or extracellular matrix components, thereby avoiding non-specific labelling artefacts. Imaging was performed immediately after labelling. Three independent repetitions of the experiment were performed.

### Biofilm formation on polystyrene coated with extract of melon, steak tartare extract and milk for inference testing

To generate images that serve as proof-of-concept for inference, biofilms of strains L628 and L1764 were cultured on polystyrene surfaces coated with extracts of cantaloupe melon, steak tartare and ultra-high temperature (UHT) milk. These extracts were selected based on the types of foods associated with *L. monocytogenes* outbreaks. The surface-coating approach mimics conditions found in food processing environments, where biofilms form on equipment or contact surfaces carrying residual food material, which is one of the most relevant contamination routes in real-world food production. For extract preparation, the whole melon was treated with a stomacher, and the liquid fraction was collected; steak tartare was homogenised with an equal weight of water (1:1 *w*/*w*), and UHT milk was used as received. Large particles were removed by centrifugation at 10,000×*g* for 10 min at 4 °C, followed by filtration through a 0.45-μm pore filter. The sterile extracts were stored at −20 °C until use, and sterility was confirmed by agar smears.

The wells of µCLEAR microtiter plates were coated with 70 µl of each extract (five replicates per extract) and incubated overnight at 4 °C. Before coating, steak tartare and milk extracts were diluted 50-fold in sterile distilled water to prevent interference with subsequent imaging and biofilm development; melon extract was used undiluted. After incubation, the wells were washed with phosphate-buffered saline to remove unbound extract. Bacterial suspensions were then added, and biofilms were allowed to develop under the conditions described above. Labelling was performed as previously described. The experiment was conducted in two independent biological replicates.

### Confocal microscopy

Biofilm imaging was performed using a Zeiss LSM10 confocal laser scanning microscope (Carl Zeiss, Germany) with a 63× (1.40 NA) oil immersion objective. Imaging was performed with a 488-nm excitation laser (30%) and 493-554 emission filters with a 54-µm pinhole, with master gain and digital offset adjusted for a quality image (metadata are available at 10.5281/zenodo.20377151). Ten micrometres of biofilm (from the bottom of the plate upwards) were visualised by acquiring 21 images with a *z*-step of 0.5 µm, an image size of 1024 × 1024 and a field of view of 134 µm × 134 µm. Image acquisition was automated using the microscope’s autofocus function, which ensured consistent scanning across all wells and positions. Images were acquired across 5–10 technical replicates (wells per strain), five positions per well, and all three independent biological replicates. This broad sampling scheme was designed to capture the natural range of biological and technical variability, including minor acquisition imperfections, rather than to produce a narrowly controlled dataset. This approach trains the model under conditions representative of routine automated imaging. The images captured in lsm format were exported to tif format using the ImageJ macro. Representative images for each strain in the dataset are shown in Fig. S2.

### Visual classification of biofilm images

To establish a human performance baseline for interpreting algorithmic classification accuracy, three laboratory members with no prior experience in biofilm image interpretation independently classified a subset of images. Ten images per strain were selected from the full dataset (80 images in total) and divided into two groups: a familiarisation set with strain identity labels available and a prediction set with strain identity masked, available at 10.5281/zenodo.20390961. Evaluators were instructed to use the familiarisation set to learn distinguishing features and then assign strain identity to each image in the masked prediction set, taking as much time as needed. Contrast and brightness adjustment in ImageJ was permitted; no other analytical tools were provided. All predictions were made independently. Results are presented in Table S3.

### Biofilm feature calculation using MicroICS

The calculation of features was implemented using efficient NumPy-based expressions, allowing multithreaded processing of all acquired images (1000) in a batch on standard hardware. The detailed use of the pipeline is described in the user manual, available at https://github.com/SkBlaz/biofilm-classification/tree/main/manual. The systematic computation of these features from raw image data is termed feature extraction or feature generation.

Features were calculated in two different ways: (i) the feature was computed for the whole image, or (ii) the layers of the image were first considered individually, after which a per-image value was obtained by aggregating the values of individual layers. The following aggregations were used: arithmetic mean (mean), median (med), standard deviation (std), variance (var) and percentiles (q10, q25, q75 and q90). The text in brackets was used as part of the feature name in the datasets. In addition, normalised values of features were calculated for each layer.

The calculated features were related to geometric aspects of the biofilm (morphological features), texture features reflecting the spatial distribution within the biofilm, statistics-based intensity features, and features identified through large language models (LLMs). Details of the calculated features necessary to understand the calculations and aid interpretation are provided in Table S4.

Leveraging the capabilities of GPT-4o (timestamp: November 5, 2024, https://arxiv.org/abs/2303.08774), we explored the creation of new feature computation functions through symbolic reasoning and interpolation. The input to the model included all Python code used to compute existing features from 3D image data, which consisted of 20 Python functions at the time of the experiment. The goal was to expand this set with additional, complementary features that could improve model performance.

Prompt: “You are a large language model designed to write Python code based on an existing code base. Your goal is to produce efficient Python functions that compute features for the final task of strain classification. Focus on functions not entailed in the input set of functions. Make sure function interfaces remain as consistent as possible.”

In response to this prompt, the model produced over 50 feature computation suggestions (see repository at https://github.com/SkBlaz/biofilm-classification, path src/feature_generation.py). These suggestions were systematically assessed for their relevance, feasibility and potential usefulness for the classification task. Following this evaluation, seven new features were selected for integration into the final feature set. These features, labelled with the prefix “GPT” in the feature descriptions, were identified as complementary to the existing features, met the criteria for inclusion and are described in Table S4.

### Biofilm feature calculation using BiofilmQ

BiofilmQ^19^ (standalone version, without MATLAB) was used for 3D biofilm image analysis. Before import, raw lsm files were converted to individual 3D tif *z*-stacks using a custom Fiji/ImageJ script based on the Bio-Formats importer. Filenames were structured to encode strain identity, well position, timepoint, channel, and *z*-depth information required for BiofilmQ recognition and import of tif files. Physical voxel dimensions were set to d*xy* = 0.1317882 µm and d*z* = 0.5 µm. For image preprocessing, two denoising strategies were applied depending on strain characteristics. Strains L455, L394, and L1823, which exhibited higher image noise levels, were pre-processed using convolution with a kernel size of (5,3) pixels, followed by top-hat filtering with a structuring element size of 31 voxels. The remaining strains required only top-hat filtering with a structuring element size of 15 voxels, as their signal-to-noise ratio was sufficient without additional convolution. Segmentation was performed using strain-specific thresholding algorithms optimised for each strain’s biofilm. Sensitivity was optimised per strain using representative images and applied uniformly to all 125 images per strain.

Features were calculated under two object segmentation conditions: cube-based declumping with a cube size of 10 voxels (1.32 µm, approximately one bacterial cell length) and without declumping, yielding per-object and global biofilm metrics including surface area, roughness, volume fraction, substrate coverage, nearest neighbour distances, intensity statistics, Haralick texture features, and autocorrelation. Results were exported as CSV files and combined across both segmentation conditions. BiofilmQ features were subsequently merged with MicroICS features for all strains solely for a learning benchmark assessment, evaluating how the inclusion of biologically oriented 3D object-based metrics affects classification performance and feature importance ranking compared to general image metrics alone. These combined features were not used for inference or statistical analysis of individual biofilm features.

### Uniform manifold approximation and projection (UMAP) generation

UMAP is a non-linear dimensionality reduction technique that optimises for a low-dimensional embedding, aiming to preserve the original fuzzy topological structure^41^. First, UMAP computes a k-nearest neighbours graph (k-NNG) in the original feature space and estimates local distances using a smooth, continuous probability distribution. It then builds a corresponding fuzzy simplicial set representation, which captures both local and global manifold structure. Through stochastic gradient descent minimising cross-entropy between the high- and low-dimensional fuzzy sets, UMAP finds an embedding that reflects underlying data geometry. In our analyses, we generated UMAP projections using the umap-learn Python package (https://umap-learn.readthedocs.io/en/latest/), configuring it for binary feature inputs to visualise sample clustering based on MicroICS-generated features in two dimensions.

### Performance comparison of ML models

After computing features for a given set of 3D biofilm images, ML models were built and evaluated. Although the dataset used in this work—approximately one thousand 3D images for the final experiments—is at the upper end of what is typically reported for this type of analysis, stratified cross-validation was used as the main evaluation procedure to ensure robust and unbiased performance estimates. The dataset was divided into three stratified folds, ensuring equal class distribution in each, with two folds used for training and one for testing in turn. This procedure was repeated five times with different random splits to reduce instability arising from any particular data partition and to provide a more reliable estimate of generalisation performance than a single train/test split. The average accuracy across all folds and repeats is reported.

The following ML classification algorithms were used to benchmark the predictive performance: random forests, extreme gradient-boosted trees (XGB), logistic regression, k-NN classifier with hyperparameter optimisation (via cross-validation on the training set) and AutoGluon, a state-of-the-art AutoML approach that was recently shown to offer competitive performance across many real-life tabular data sets^42^.

In addition, we designed the experiments to examine the effects of feature types and dimensionality reduction. The experiments were divided into two main categories: one that included count-based features and one that excluded them, as count-based features are the most frequently represented features and are not easily understood intuitively. Additionally, singular value decomposition (SVD) was evaluated as a dimensionality reduction technique. Given the high dimensionality of the dataset, SVD was implemented to reduce the number of dimensions to 32, 64, 128, 256, or 512 (with up to 15 iterations) to mitigate potential issues with algorithms less suited to high-dimensional inputs while preserving variance in the data^43^.

Comparison included hyperparameter tuning for the evaluated classifiers as follows:

- Random forest (RF): the scikit-learn implementation with default parameters was used, amounting to 100 estimators.
- XGB: the binary:logistic objective function was employed, with 100 trees, each limited to a maximum depth of three. Other hyperparameters were set to default values.
- Logistic regression: the scikit-learn implementation with default parameters was used, serving as a weak baseline for comparison.
- k-NN: the scikit-learn implementation with default parameters was used. Additionally, GridSearchCV was used to optimise the number of neighbours, ranging between 3 and 30.
- AutoGluon: the default Tabular Classifier object was used without any manual configuration.
- The majority classifier was considered a weak baseline.

### Implementation of feature ranking

As we refined our microscopy imaging and ML analysis, we used feature ranking to identify the most important predictors^44^. This approach assigns numeric scores to features, representing the predictive capacity of a feature with respect to the target variable. To conduct feature ranking, we utilised the random forest algorithm - a gold-standard approach for tree ensemble-based feature ranking^45^. In the later stage of the process, feature ranking was also used to conduct ablation studies. By considering only the top k features according to the ranking, we were able to assess approximately how many of the generated features are required for sufficient predictive performance.

### Statistical analysis

We performed statistical analysis on the 81 most important features (from feature ranking) to understand each feature’s individual contribution to strain classification (identity task). A one-way multivariate analysis of variance was performed to compare the strains based on the feature values. The measurement times were included as blocks in the model. This was followed by one-way ANOVAs for all features with Bonferroni’s Type I error correction to avoid inflation of the familywise error rate.

Prior to the analysis, constant features and features with multiple negative values were eliminated. The GPT correlation calculations were in the range of 10^−7^ for several images, especially for strain L1323, which was notably different from the other calculations, so these calculations were not included. The statistical analysis was finally performed for 72 features.

The normality of the model residuals was accepted based on the absolute values of their skewness and kurtosis, which were below 1 and 2, respectively.

Finally, pairwise comparisons were performed using the Games-Howell post hoc test, a statistical procedure designed to address the violation of the assumption of homogeneity of variances.

Pearson’s correlation coefficients were calculated for the 20 top features to analyse their pairwise relationships.

The statistical analysis was performed in the programming environment R (version 4.2.2, R Core Team, 2024). The visualisation was carried out with the package “ggplot2”^46^.

### Ablation studies to optimise data acquisition

We conducted two studies to determine how the amount of data used affects the predictive power of the trained random forest model. Since 3D biofilm imaging is laboratory-work intensive and time-consuming, we wanted to identify the minimum amount of data needed to obtain a useful model while reducing labour costs.

In the first study, we varied the number of layers used from the full set of 21 image layers down to a single layer, to assess whether all 21 image layers (required for a vertical resolution of 0.5 µm) are necessary or if fewer layers can be used without compromising model performance, thereby increasing the vertical resolution.

In the second study, we investigated how many different wells (corresponding to technical repetitions) need to be analysed (from 1 to 25) without compromising the performance of the prediction model, based on the 25 wells used in this study.

We artificially varied the vertical resolution of the biofilm measurements. Starting with measurements at a full resolution of 21 layers, then reducing this to 11 layers by omitting every other layer, and similarly to 6, 3, 2 and 1 layers, leaving only the bottom layer. Each layer resolution thus resulted in a separate data set of images. These datasets were then run through the entire pipeline, training the random forest model as described above.

A pipeline was used to investigate the relationship between the number of wells tested and the predictive performance of a trained model. This was an iterative process where, in each iteration, the training data was filtered to include only samples from a specified number of wells, ranging from 1 to the maximum number of wells (25). Here, in contrast to the layer reduction ablation study, the calculated features were only ever aggregated over the different wells, and reducing the number of wells only increases the volatility of the aggregated values. A random forest model was then trained on this data for each iteration and evaluated on a separate test set. The experiment was repeated multiple times for each number of pools, and the results were aggregated to obtain the mean and standard deviation of the predictive performance (accuracy) of the models.

### Inference

The inference procedure was implemented as an integral component of the analytical framework. Each training iteration produced a set of stored models together with the corresponding feature-construction routines; inference was therefore incorporated directly within the software environment (Docker image) to ensure reproducibility and ease of reuse across experiments.

The inference module was optimised using images from two epidemiologically and structurally distinct strains, L628 and L1764. The first dataset included control images from independent experiments captured under the same conditions as the training set, providing known strain identities for accurate performance evaluation. Stratified random sampling, repeated five times for robustness, was used to select 50% of control images for inclusion in the training set through data augmentation to maintain class balance and capture experimental variability, while the remaining 50% served as an independent test set for inference. This approach notably improved inference accuracy by incorporating real experimental variability directly into model training.

A second dataset of food extract-perturbed biofilms further validated model generalisation to structurally altered morphologies.

During inference, pre-trained models were applied to previously unseen data. The input data were first transformed into a feature space congruent with that of the training phase, after which predictions were generated.

Model outputs included class-specific prediction probabilities and corresponding Shapley values (SHAP), which provide interpretable, instance-level explanations of feature contributions. SHAP values explain ML predictions by quantifying each image feature’s contribution to the output. Positive (higher) values indicate a stronger influence towards a specific class prediction, while negative values reduce its likelihood, making model decisions interpretable. Importantly, SHAP values do not simply restate structural descriptor values; they quantify each feature’s contribution to a specific prediction in the context of all other features considered simultaneously by the model. This distinguishes ML-based interpretation from standard univariate comparisons of structural descriptors, which cannot capture feature interactions or rank predictive importance across a high-dimensional feature space.

### Inference of strain identity and clinical association of strains in the given dataset

Strain identity inference used strain names (ATCC 19115, L628, L455, L634, L1323, L1823, L394, L1764) directly as classes, with labels extracted from image filenames. For clinical association inference, strains were classified by CC into clinically associated (L628, L455, L634, L1823, ATCC 19115; CC1, CC2, CC4, CC6) and non-clinically associated (L1764, L394, L1323; CC121, CC8, CC9) groups, rather than by isolation source (food/feed vs human/animal). This CC-based classification currently provides the most robust, globally standardised division for *L. monocytogenes*, as CCs integrate genomic and epidemiological data across studies, transcending variable source metadata. Strain names in image filenames were replaced using this grouping so that labels could again be extracted from the image names.

Both tasks used identical model training procedures. Among the tested algorithms, random forest was selected for its optimal performance and interpretability of feature importance.

### Streamlining end-to-end analysis

Feature extraction, classifier comparison, and inference are the main computational steps in the analysis of 3D image datasets (Fig. 1). Feature extraction is implemented using efficient NumPy-based expressions and can process batches of 1000 3D images on standard laptop hardware. Intermediate outputs are saved during execution so that partial computation is not lost if a subsequent step fails or is interrupted. To ensure broad accessibility for domain experts without extensive programming experience, we developed a containerised analysis framework executable from a standard command-line interface. Docker enables containerisation of software that typically consists of multiple libraries and shell invocations, making it not directly portable across systems. However, with Docker, the produced image can be run on both Unix- and Windows-based machines once built, eliminating the need to manually manage dependencies. The Docker image uses an Ubuntu 22 base and contains all dependencies and code required to run the analysis as soon as the user has a dedicated folder populated with 3D images.

The default workflow uses random forest as the primary classifier, chosen for its strong predictive performance and interpretability, as well as its favourable balance of runtime and memory demand, remaining feasible on standard laptops without parallelisation. More computationally intensive options, including AutoGluon and TPOT, are available but may require substantially greater memory and processing time. User-defined parallelisation is supported for larger datasets. Scalability to high-throughput applications depends on available computational resources and on imaging throughput upstream of the pipeline. Project domain experts validated the tool prior to deployment, supporting the assumption that other domain users can operate it without specialist computational knowledge. A detailed manual is provided to ensure usability https://github.com/SkBlaz/biofilm-classification.

### Ethics and biosecurity

This article does not contain any studies with human participants or animals performed by any of the authors.

## Supplementary information

Supplementary_information_Biofilms&Microbiome_revised.

## Data Availability

All materials associated with the manuscript are available at Zenodo: https://doi.org/10.5281/zenodo.20377151 and https://doi.org/10.5281/zenodo.20390961.

## Code availability

The code is available on GitHub at https://github.com/SkBlaz/biofilm-classification.
