# Supplementary material for: MicroICS: predictive phenotyping of Listeria monocytogenes biofilms from three-dimensional structural features
Source: NPJ Biofilms Microbiomes. 2026 Jul 8;12:216. doi: 10.1038/s41522-026-01083-8 (PMC13724688; doi:10.1038/s41522-026-01083-8)

MicroICS: Predictive phenotyping of *Listeria monocytogenes* biofilms from three-dimensional structural features

Supplementary information

Nika Janež[1], Blaž Škrlj[2], Aljaž Osojnik[2], Márta Ladányi[3], Martin Breskvar[2], Matej Petković[2], Boštjan Kokot[4,5], Petra Čotar[4], Bojan Papić[6], Majda Golob[6], Tjaša Peternel[1], Jerica Sabotič[1]

[1] Department of Biotechnology, Jožef Stefan Institute, Ljubljana, Slovenia
[2] Department of Knowledge Technologies, Jožef Stefan Institute, Ljubljana, Slovenia
[3] Department of Applied Statistics, Institute of Mathematics and Basic Science, Hungarian University of Agriculture and Life Sciences (MATE), Budapest, Hungary
[4] Department of Condensed Matter Physics, Jožef Stefan Institute, Ljubljana, Slovenia
[5] Centre for Advanced Optical Microscopies, Jožef Stefan Institute, Ljubljana, Slovenia
[6] University of Ljubljana, Veterinary Faculty, Institute of Microbiology and Parasitology, Ljubljana, Slovenia

Table S1. Epidemiology, genotypes and sequencing details of the eight *L. monocytogenes* strains used in this study.

Name of strain	L628	L455	L1764	L394	L634	L1323	L1823	ATCC 19115
**Sequence type**	ST1	ST1	ST121	ST9	ST4	ST8	ST6	ST2
**Clonal complex**	CC1	CC1	CC121	CC9	CC4	CC8	CC6	CC2
**Source**	Animal clinical	Animal feed	Food	Food	Animal subclinical	Human clinical	Food	Human clinical
**Isolation year**	2014	2012	2020	2010	2014	2013	2020	/
**Description**	Cow brain (meningoencephalitis)	Silage	RTE meat product (deer salami)	Fresh fruit	Raw cow milk (subclinical mastitis)	Human blood (rhombencephalitis), outbreak 2013)	RTE meat product (smoked fish)	/
**NCBI (SRA) accession number**	SRR9067586	SRR30853287	ERR10549951	SRR30853286	SRR9067593	SRR30853285	SRR30853284	JAJOHJ000000000.1
**Sequencing technology**	Ion Torrent PGM	Illumina NextSeq 500	Illumina NextSeq 500	Illumina NextSeq 500	Ion Torrent PGM	Illumina MiSeq	Illumina NovaSeq 6000	Illumina MiSeq
**Publication**	Papić et al. 2019 doi: 10.1111/jam.14418	This study	Karlsmore et al. 2024 doi: 10.1016/j.meegid.2024.105582	This study	Papić et al. 2019 doi: 10.1111/jam.14418	This study	This study	NCBI and ATCC

Table S2. The presence (+) or absence (-) of genetic markers in the genomes of the eight *L. monocytogenes* strains used in this study that are associated with virulence, biofilm formation, antimicrobial resistance, stress response and wall teichoic acid synthesis.

Name of gene/protein	Biomarker	Role of biomarker	L628	L455	L1764	L394	L634	L1323	L1823	ATCC 19115
**ActA**	pathogenicity, biofilm phenotype associated protein	motility	-	-	-	+	-	+	+	+
**AgrA**	pathogenicity	autoinducer (communication)	+	+	+	+	+	+	+	+
**Ami**	pathogenicity, biofilm phenotype associated protein	adherence	-	-	+	+	-	+	-	-
**Aut**	pathogenicity	invasion (Auto protein)	-	-	+	+	-	+	-	-
**FlaA**	pathogenicity, biofilm phenotype associated protein	motility	+	+	+	+	+	+	+	+
**FlgC**	pathogenicity	motility	+	+	+	+	+	+	+	+
**FlgE**	pathogenicity	motility	+	+	+	+	+	+	+	+
**GadA**	pathogenicity	survival at low pH	-	-	-	+	-	+	-	-
**GadB**	pathogenicity	survival at low pH	+	+	+	+	+	+	+	+
**GadC**	pathogenicity	survival at low pH	+	+	+	+	+	+	+	+
**Gmar**	pathogenicity	antirepressor; regulates flagellar motility	+	+	+	+	+	+	+	+
**Lap**	pathogenicity	adherence	+	+	+	+	+	+	+	+
**LapB**	pathogenicity	adherence	+	+	+	+	+	+	+	+
**OatA**	pathogenicity	immune modulation	+	+	+	+	+	+	+	+
**OppA**	pathogenicity	adherence	+	+	+	+	+	+	+	+
**OrfX**	pathogenicity	survival within macrophages and in the infected host	+	+	+	+	+	+	+	+
**OrfZ**	pathogenicity	part of PrfA regulon	+	+	+	+	+	+	+	+
**Rli55**	pathogenicity	riboswitch-regulated noncoding regulatory RNA that function by sequestering a protein	+	+	+	+	+	+	+	+
**Rli60**	pathogenicity	regulation of virulence (controls the internal isoleucine pool and CodY activity)	+	+	+	+	+	+	+	+
**Rsbv**	pathogenicity	tolerance under environmental stress conditions and regulation of virulence	+	+	+	+	+	+	+	+
**biLE**	pathogenicity	PrfA-regulated bile exclusion system	+	+	+	+	+	+	+	+
**bsh**	pathogenicity	bile salt resistance	+	+	+	+	+	+	+	+

Name of gene/protein	Biomarker	Role of biomarker	L628	L455	L1764	L394	L634	L1323	L1823	ATCC 19115
**btlB**	pathogenicity	gastrointestinal persistence and bile tolerance	+	+	+	+	+	+	+	+
**chiA**	pathogenicity	suppression of host innate immunity	+	+	+	+	+	+	+	+
**clpB**	pathogenicity	induced thermotolerance and virulence	+	+	+	+	+	+	+	+
**clpc**	pathogenicity	ATPase promoting early escape from the phagosome of macrophages (survival under stress conditions)	+	+	+	+	+	+	+	+
**clpe**	pathogenicity	ATPase required for prolonged survival at 42 °C (survival under stress conditions)	+	+	+	+	+	+	+	+
**clpp**	pathogenicity	serine protease involved in proteolysis; survival under stress conditions	+	+	+	+	+	+	+	+
**codY**	pathogenicity	metabolic regulator that directly activates *prfA*	+	+	+	+	+	+	+	+
**ctaP**	pathogenicity	cysteine transport, acid resistance, bacterial membrane integrity, adherence to host cells	+	+	+	+	+	+	+	+
**ctsR**	pathogenicity	regulator that affects piezotolerance, stress resistance, motility and virulence	+	+	+	+	+	+	+	+
**dal**	pathogenicity	alanine racemase involved in the synthesis of D-alanine	+	+	+	+	+	+	+	+
**degU**	pathogenicity	response regulator that affects biofilm formation, chemotaxis, motility and heat stress resistance	+	+	+	+	+	+	+	+
**dltA**	pathogenicity	LTA synthesis	+	+	+	+	+	+	+	+
**fbpA**	pathogenicity	adherence	+	+	+	+	+	+	+	+
**fri**	pathogenicity	stress tolerance and intracellular multiplication in macrophage-like cells	+	+	+	+	+	+	+	+
**fur**	pathogenicity	Fur regulator of ferric iron uptake	+	+	+	+	+	+	+	+
**hfq**	pathogenicity	tolerance to osmotic and ethanol stress; long-term survival under amino acid-limiting conditions	+	+	+	+	+	+	+	+
**hly**	pathogenicity	exotoxin listeriolysin O	+	+	+	+	+	+	+	+
**htrA**	pathogenicity	enhanced bacterial survival under stress conditions	+	+	+	+	+	+	+	+
**hupC**	pathogenicity	heme uptake, virulence	+	+	+	+	+	+	+	+
**iap**	pathogenicity	invasion (p60 protein), cell divison	+	+	+	+	+	+	+	+
**inlA**	pathogenicity, biofilm phenotype associated protein	invasion	+	+	-	+	+	+	+	+
**inlA (truncated)**	decreased pathogenicity and	decreased invasion	-	-	+	-	-	-	-	-

Name of gene/protein	Biomarker	Role of biomarker	L628	L455	L1764	L394	L634	L1323	L1823	ATCC 19115
	biofilm phenotype associated protein									
**inlB**	pathogenicity and biofilm phenotype associated protein	invasion	+	+	+	+	+	+	+	+
**inlC**	pathogenicity	immune modulation	+	+	+	+	+	+	+	+
**inlF**	pathogenicity	adherence	-	-	-	+	-	+	-	-
**inlH**	pathogenicity	evasion of host defenses	+	+	+	+	+	+	+	+
**inlJ**	pathogenicity	adherence	+	+	+	+	+	+	+	+
**inlK**	pathogenicity	immune modulation	-	-	+	+	-	+	-	-
**lgt**	pathogenicity	lipoprotein anchoring, intracellular growth	+	+	+	+	+	+	+	+
**lhrC**	pathogenicity	sRNAs involved in intracellular growth in macrophages	+	+	+	+	+	+	+	+
**lipA**	pathogenicity	secreted phosphatase involved in virulence	+	+	+	+	+	+	+	+
**lisK**	pathogenicity	two-component signal transduction system LisRK involved in tolerance to nisin and cephalosporins	-	-	+	+	-	-	-	-
**lisR**	pathogenicity	two-component signal transduction system LisRK involved in tolerance to to nisin and cephalosporins	+	+	-	-	+	+	+	+
**lmo0514**	pathogenicity	survival in plasma and during infection of mice	+	+	+	+	+	+	+	+
**lmo0610**	pathogenicity	sigma B-regulated internalin-like protein	-	-	+	+	-	+	-	-
**lmo2026**	pathogenicity	inlL; adherence	-	-	-	+	-	-	-	-
**lmo2085**	pathogenicity	putative peptidoglycan bound protein with an LPXTG motif	+	+	+	+	+	+	+	+
**lntA**	pathogenicity	immune modulation	+	+	+	+	+	+	+	+
**lpeA**	pathogenicity	invasion into non-professional phagocytes	+	+	+	+	+	+	+	+
**lplA1**	pathogenicity	use of host metabolites for *in vivo* growth	+	+	+	+	+	+	+	+
**lsp**	pathogenicity	post-translational modification; surface protein anchoring	+	+	+	+	+	+	+	+
**mogR**	pathogenicity	regulation of flagellar motility gene expression; required for full virulence	+	+	+	+	+	+	+	+
**mpl**	pathogenicity	exoenzyme, protease	+	+	+	+	+	+	+	+
**mprf**	pathogenicity	resistance to cationic antimicrobial peptides (CAMPs)	+	+	+	+	+	+	+	+
**murA**	pathogenicity	peptidoglycan turnover	+	+	+	+	+	+	+	+

Name of gene/protein	Biomarker	Role of biomarker	L628	L455	L1764	L394	L634	L1323	L1823	ATCC 19115
**perR**	pathogenicity	tolerance to hydrogen peroxide	+	+	+	+	+	+	+	+
**pgdA**	pathogenicity	immune modulation; resistance to lysozyme	+	+	+	+	+	+	+	+
**pgl**	pathogenicity	lmo0558; growth in glucose-limiting conditions, resistance to oxidative stress	+	+	+	+	+	+	+	+
**plcA**	pathogenicity	exotoxin, phospholipase	+	+	+	+	+	+	+	+
**plcB**	pathogenicity	exotoxin, phospholipase	+	+	+	+	+	+	+	+
**prfA**	pathogenicity	regulation	+	+	+	+	+	+	+	+
**prsA2**	pathogenicity	post-translational modification	+	+	+	+	+	+	+	+
**pycA**	pathogenicity	replication in mammalian macrophages and epithelial cells	+	+	+	+	+	+	+	+
**recA**	pathogenicity	DNA repair, activator of the SOS response	+	+	+	+	+	+	+	+
**relA**	pathogenicity	growth after adhesion to a model surface	+	+	+	+	+	+	+	+
**secA2**	pathogenicity	required for virulence in mice and cell–cell spread in cultured cells	+	+	+	+	+	+	+	+
**sigB**	pathogenicity	alternative sigma factor Sigma B controlling the general stress response regulon	+	+	+	+	+	+	+	+
**sipX**	pathogenicity	signal peptidase SipX required for full virulence	+	+	+	+	+	+	+	+
**sipZ**	pathogenicity	signal peptidase SipZ required for full virulence	+	+	+	+	+	+	+	+
**sod**	pathogenicity	superoxide dismutase; detoxification reactive oxygen intermediates	+	+	+	+	+	+	+	+
**srtA**	pathogenicity	binding LPXTG proteins to the cell wall	+	+	+	+	+	+	+	+
**srtB**	pathogenicity	binding LPXTG proteins to the cell wall	+	+	+	+	+	+	+	+
**stp**	pathogenicity	phosphatase required for growth in a murine model of infection	+	+	+	+	+	+	+	+
**svpA**	pathogenicity	nutritional/metabolic factor	+	+	+	+	+	+	+	+
**tcsA**	pathogenicity	CD4+ T cell-stimulating antigen	+	+	+	+	+	+	+	+
**tig**	pathogenicity	trigger factor; enhanced survival in the presence of heat and ethanol	+	+	+	+	+	+	+	+
**uhpT**	pathogenicity	nutritional/metabolic factor	+	+	+	+	+	+	+	+
**vip**	pathogenicity	invasion	-	-	-	+	-	-	-	-
**virR**	pathogenicity	regulator of virulence and resistance to nisin	+	+	+	+	+	+	+	+
**fosX (lmo1702)**	antimicrobial resistance	fosfomycin resistance	+	+	+	+	+	+	+	+

Name of gene/protein	Biomarker	Role of biomarker	L628	L455	L1764	L394	L634	L1323	L1823	ATCC 19115
**ClpL**	stress survival	heat resistance	-	-	+	-	-	-	-	-
**Tn6188_qac (ermC)**	antimicrobial resistance	tolerance to benzalkonium chloride	-	-	+	-	-	-	+	+
**lmo0444**	stress survival	stress survival islet (SS1)	-	-	-	+	-	+	-	-
**lmo0445**	stress survival	stress survival islet (SS1)	-	-	-	+	-	+	-	-
**lmo0446 (pva)**	stress survival	stress survival islet (SS1)	-	-	-	+	-	+	-	-
**lmo0447 (gadD1)**	stress survival	stress survival islet (SS1)	-	-	-	+	-	+	-	-
**lmo0448 (gadT1)**	stress survival	stress survival islet (SS1)	-	-	-	+	-	+	-	-
**lin0464**	stress survival	survival upon oxidative and alkaline stress (SS2)	-	-	+	-	-	-	-	-
**lin0465**	stress survival	survival upon oxidative and alkaline stress (SS2)	-	-	+	-	-	-	-	-
**ilsA**	pathogenicity	pathogenicity island LIPI-3; bacteriocin listeriolysin S that modulates the host microbiota	+	+	-	-	+	-	+	+
**ilsG**	pathogenicity	pathogenicity island LIPI-3	+	+	-	-	+	-	+	+
**ilsH**	pathogenicity	pathogenicity island LIPI-3	+	+	-	-	+	-	+	+
**ilsX**	pathogenicity	pathogenicity island LIPI-3	+	+	-	-	+	-	+	+
**ilsB**	pathogenicity	pathogenicity island LIPI-3	+	+	-	-	+	-	+	+
**ilsY**	pathogenicity	pathogenicity island LIPI-3	+	+	-	-	+	-	+	+
**ilsD**	pathogenicity	pathogenicity island LIPI-3	+	+	-	-	+	-	+	+
**ilsP**	pathogenicity	pathogenicity island LIPI-3	+	+	-	-	+	-	+	+
**LM9005581_70009**	pathogenicity	pathogenicity island LIPI-4 (putative cellobiose-family phosphotransferase system)	-	-	-	-	+	-	-	-
**LM9005581_70010**	pathogenicity	pathogenicity island LIPI-4	-	-	-	-	+	-	-	-
**LM9005581_70011**	pathogenicity	pathogenicity island LIPI-4	-	-	-	-	+	-	-	-
**LM9005581_70012**	pathogenicity	pathogenicity island LIPI-4	-	-	-	-	+	-	-	-
**LM9005581_70013**	pathogenicity	pathogenicity island LIPI-4	-	-	-	-	+	-	-	-
**LM9005581_70014**	pathogenicity	pathogenicity island LIPI-4	-	-	-	-	+	-	-	-
**TarO1**	WTA synthesis	WTA backbone synthesis	+	+	+	+	+	+	+	+
**TarO2**	WTA synthesis	WTA backbone synthesis	+	+	+	+	+	+	+	+
**TagA**	WTA synthesis	WTA backbone synthesis	+	+	+	+	+	+	+	+

Name of gene/protein	Biomarker	Role of biomarker	L628	L455	L1764	L394	L634	L1323	L1823	ATCC 19115
**Tag B**	WTA synthesis	WTA backbone synthesis	+	+	+	+	+	+	+	+
**TagD**	WTA synthesis	WTA backbone synthesis	+	+	+	+	+	+	+	+
**TarF**	WTA synthesis	WTA backbone synthesis	+	+	-	-	+	+	+	+
**TarI**	WTA synthesis	WTA backbone synthesis	+	+	+	+	+	+	+	+
**TarJ**	WTA synthesis	WTA backbone synthesis	+	+	+	+	+	+	+	+
**TarK**	WTA synthesis	WTA backbone synthesis	-	-	-	-	-	-	-	-
**TarL**	WTA synthesis	WTA backbone synthesis	-	+	+	+	+	+	-	-
**Liv1073**	WTA synthesis	glycosyltransferase GT-2 (possibly type II WTA backbone synthesis)	+	+	+	+	+	+	+	+
**GtcA**	pathogenicity, WTA synthesis	WTA flippase	+	+	+	+	+	+	+	+
**GalU**	WTA synthesis	WTA galactosylation of non4b serotype	+	+	+	+	+	+	+	+
**GlcV (GtcB)**	WTA synthesis	WTA galactosylation of non4b serotype	-	+	+	+	+	+	-	+
**PmpA**	WTA synthesis	WTA galactosylation of non 4b serotype	+	+	-	-	+	-	+	+
**GltA**	WTA synthesis	WTA glucosylation in 4b serotype	+	+	-	-	+	-	+	+
**TarS**	WTA synthesis	glycosylation by GlcNAC in Sa	-	-	+	+	-	+	+	+
**TarP**	WTA synthesis	glycosyltransferase in Sa	-	-	-	-	-	-	-	-
**Lmo2550**	WTA synthesis	WTA glycosylation by GlcNac in 1/2a serotype	+	-	+	+	-	+	-	+
**Lmo1079**	WTA synthesis	WTA glycosylation by GlcNac in 1/2a serotype	-	-	+	+	-	+	-	-
**RmlA**	WTA synthesis	WTA rhamnosylation in 1/2a serotype	-	-	+	+	-	+	-	-
**RmlT**	WTA synthesis	WTA rhamnosylation in 1/2a serotype	-	-	+	+	-	+	-	-
**Lmo2229 = PBP4**	biofilm phenotype associated protein	Peptidoglycan turnover	+	+	+	+	+	+	+	+
**Lmo2554**	biofilm phenotype associated protein	LTA synthesis	+	+	+	+	+	+	+	+
**Lmo0644**	biofilm phenotype associated protein	LTA synthesis	+	+	+	+	+	+	+	+
**Lmo 2056**	biofilm phenotype associated protein	secreted protein	+	+	+	+	+	+	+	+
**BapL = Lmo0435**	biofilm phenotype associated protein	LPXTG protein involved in colonization	-	-	+	+	-	-	-	-
**Lmo2026 = InlL**	biofilm phenotype associated protein	LPXTG protein involved in colonization	-	-	-	+	-	-	-	-
**Lmo0676 = FliP**	biofilm phenotype associated protein	flagellar biosynthesis	+	+	+	+	+	+	+	+

Name of gene/protein	Biomarker	Role of biomarker	L628	L455	L1764	L394	L634	L1323	L1823	ATCC 19115
**Lmo0196 = SpoV**	biofilm phenotype associated protein	regulator|global regulatory factor	+	+	+	+	+	+	+	+
**Lmo0197 = SpoV**	biofilm phenotype associated protein	global regulatory factor	+	+	+	+	+	+	+	+

Figure S1. A dendrogram showing genetic similarity among strains, based on marker genes associated with virulence, antimicrobial resistance, teichoic acid synthesis, and biofilm formation (Table S2). The main similarities and differences are summarised to illustrate the genetic background of the strains. All strains harboured the virulence factor *inlA*, which is essential for intestinal invasion, except for strain L1764, which has a truncated and thus non-functional InlA. Other key virulence genes - including the regulator *prfA*, the exotoxins *plcA* and *plcB* and the haemolysin *hly* — were present in all strains. Genes associated with biofilm formation overlap with those involved in cell wall synthesis and other essential cell functions and were also present in all strains. Strains L455, ATCC 19115, L628, L634 and L1823 carried the pathogenicity island LIPI-3, which encodes listeriolysin S that enables *L. monocytogenes* to escape from a host cell vacuole into cytosol. These strains clustered together in the dendrogram and belonged to hypervirulent CCs (Disson et al. 2021,doi: 10.1016/j.tim.2021.01.008). They also harboured genes for WTA galactosylation and glucosylation. Strain L634 also contained pathogenicity island LIPI-4, which is associated with brain and placental invasion. In contrast, strains L1323, L394 and L1764 form a separate cluster belonging to clonal complexes (CC121, CC9, CC8) that are not normally associated with clinical cases (Disson et al. 2021, doi: 10.1016/j.tim.2021.01.008). They harboured stress response genes and WTA rhamnosylation genes that are associated with biofilm formation (Park et al. 2022, doi: 10.1128/spectrum.02769-22). Taken together, the comparative genomic analysis confirmed the high genetic diversity of the studied strains.

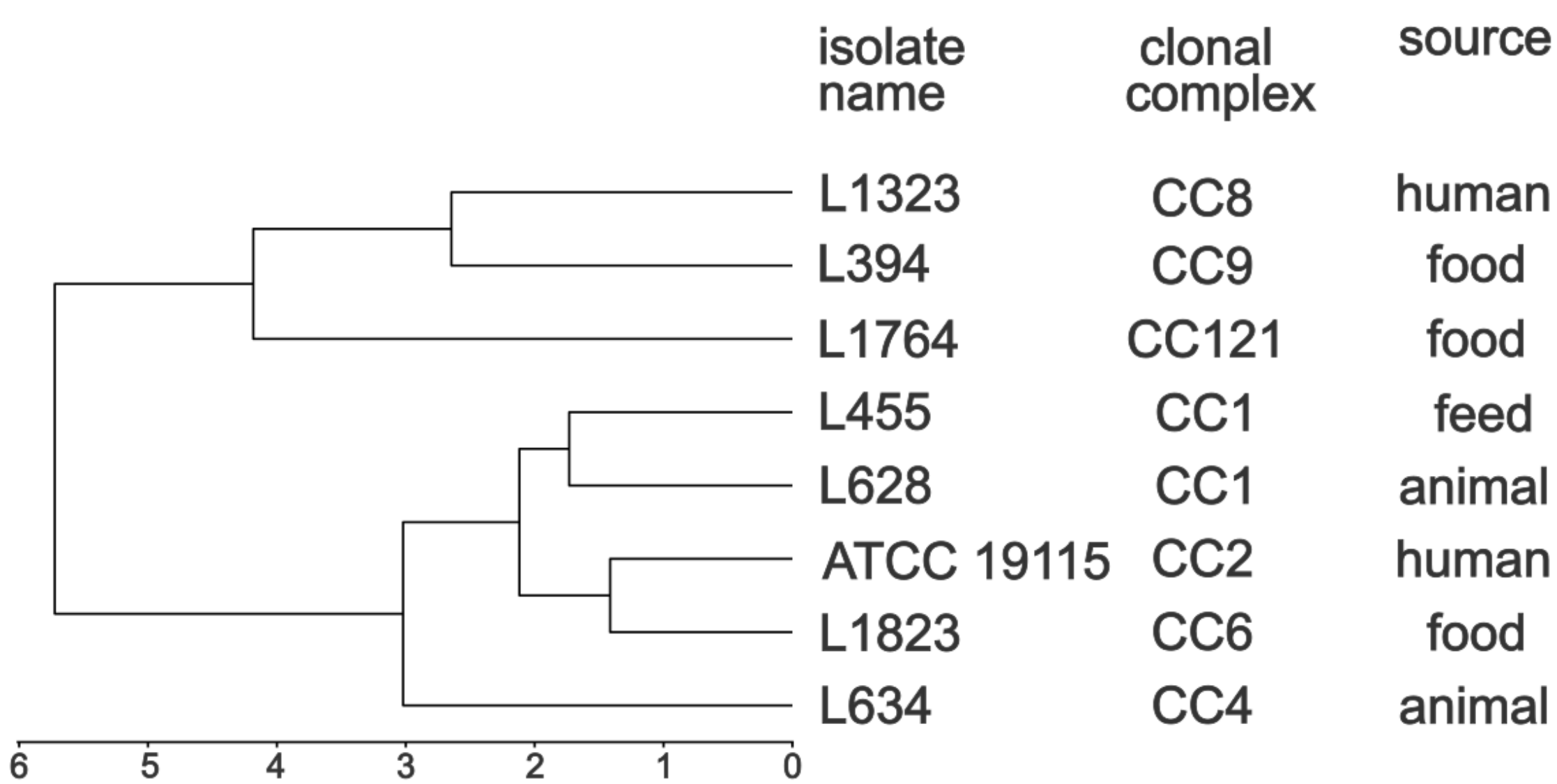

Figure S2. Confocal microscopy images highlighting structural differences in biofilms formed by the selected strains, showing representative views from the bottom and middle layers of the biofilm. Two very different biofilm structures were found visually. Strain ATCC 19115 formed dense biofilms concentrated in the bottom layers of the 3D image, with microcolonies on the surface, whereas the biofilm of strain L1764 was distributed homogeneously throughout the entire volume. Strains L628, L634, L1323, L1823, L394 and L455 exhibited biofilm characteristics that lay between these two extremes. There was also a notable difference in the brightness of the images of strain ATCC 19115, which was repeatedly lower compared with the other strains, less bright was also L1323, but the rest of the strains did not stand out.

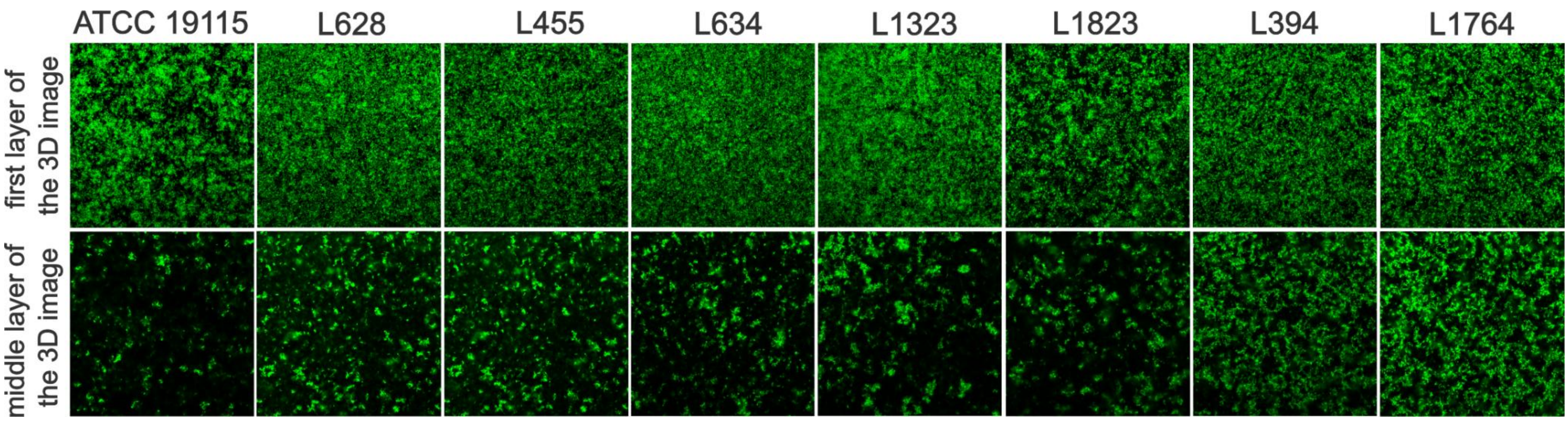

Table S3. Results of the visual classification conducted by three laboratory members. They were provided with a set of 40 images displaying the original strain names to use as a training test, followed by a different set of 40 images of the same strains with the names concealed (masked). They predicted the strain name for each image to the best of their knowledge. A brief description of their approach is provided below.

		Person 1		Person 2		Person 3		
**name of the mask**	**strain on the image with masked name**	**predicted strain**	**correct prediction (1), incorrect prediction (0)**	**predicted strain**	**correct prediction (1), incorrect prediction (0)**	**predicted strain**	**correct prediction (1), incorrect prediction (0)**	**Total number of correct predictions per strain**
1	L634	L455	0	L1823	0	L634	**1**	1
2	L1764	L394	0	L628	0	L1764	**1**	1
3	L1764	L1823	0	L394	0	L1764	**1**	1
4	L394	L394	**1**	L1764	0	L394	**1**	2
5	L455	L455	**1**	L634	0	L19115	0	1
6	L634	L1323	0	L1823	0	L1323	0	0
7	L394	L455	0	19115	0	L394	**1**	1
8	L455	L394	0	L1323	0	L634	0	0
9	L1823	L1823	**1**	L1823	**1**	L1764	0	2
10	L628	L628	**1**	L1824	0	L628	**1**	2
11	L634	L455	0	L628	0	L634	**1**	1
12	L394	L394	**1**	L394	**1**	L1323	0	2
13	L1823	L1764	0	L1823	**1**	L1823	**1**	2
14	19115	19115	**1**	L1764	0	L19115	**1**	2
15	L628	L628	**1**	L628	**1**	L628	**1**	3
16	L634	L1323	0	L629	0	L634	**1**	1
17	L394	L394	**1**	L1323	0	L394	**1**	2
18	L1323	L1323	**1**	L1823	0	L1323	**1**	2
19	L1823	L1823	**1**	L628	0	L1823	**1**	2
20	19115	19115	**1**	19115	**1**	L19115	**1**	3
21	L634	L634	**1**	L634	**1**	L634	**1**	3
22	L394	L394	**1**	L17664	0	L1764	0	1
23	L1323	L1323	**1**	L394	0	L1323	**1**	2
24	L1823	L1823	**1**	L628	0	L1823	**1**	2
25	19115	19115	**1**	19115	**1**	L19115	**1**	3
26	L1764	L1764	**1**	L394	0	L1823	0	1
27	L455	L628	0	19115	0	L628	0	0
28	L1323	L1323	**1**	L1823	0	L1323	**1**	2
29	L1823	L1823	**1**	L1824	**1**	L394	0	2
30	19115	19115	**1**	19115	**1**	L19115	**1**	3
31	L1764	L1823	0	L394	0	L1823	0	0
32	L455	L1323	0	L455	**1**	L628	0	1
33	L1323	L394	0	L628	0	L1323	**1**	1
34	L628	L628	**1**	L394	0	L628	**1**	2
35	L628	L628	**1**	L1823	0	L628	**1**	2
36	L1764	L1764	**1**	L17764	**1**	L1823	0	2
37	L455	L1323	0	L628	0	L1323	0	0
38	L1323	L1323	**1**	L634	0	L1323	**1**	2
39	19115	19115	**1**	191115	**1**	L19115	**1**	3

		Person 1		Person 2		Person 3		
**name of the mask**	**strain on the image with masked name**	**predicted strain**	**correct prediction (1), incorrect prediction (0)**	**predicted strain**	**correct prediction (1), incorrect prediction (0)**	**predicted strain**	**correct prediction (1), incorrect prediction (0)**	**Total number of correct predictions per strain**
40	L628	L455	0	L634	0	L628	**1**	1
	**number of correct predictions**		**25**		**12**		**27**	
	**% of correct predictions**		**62.5**		**30**		**67.5**	

Description of the approach to visual image classification:

Person 1: I adjusted the brightness and contrast of the images using ImageJ. Then I examined all the images from the training set, viewing images of all strains side by side, and tried to identify features that would differentiate them. I concluded that the main difference between the strains was the number of layers occupied by the biofilm. I then randomly selected one image from each strain to compare with the images from the test group. I first examined all the images at the highest z-levels and gradually eliminated the strains that differed most from the test strains at that height. For the last three to four images, I examined the entire height, comparing the lower levels – specifically, the growth on the substrate and the pattern at around z10—to determine whether they were arranged in clusters or microcolonies, and how this pattern changed with height.

Person 2: I opened and viewed all the images from the training set, then selected one image per strain to use for comparison with the images from the test set. I opened the test set images individually and compared them with the selected training set images. I examined differences in bacterial size, the height of biofilm growth, whether microcolonies formed pyramid shapes, or whether the growth was scattered within the 3D image volume.

Person 3: I examined the training set and then opened one image per strain, placing them side by side. I kept these images open while predicting the strains in the test set. The procedure for sorting the test images was as follows: first, I briefly reviewed the entire Z-stack of the image, then examined the substrate and compared it to the open images of all eight strains. Next, I compared the bacteria on the substrate in detail, focusing on size and shape. If this provided a clear indication for the prediction, I did not examine the Z-stack further. If this was insufficient to make a decision, I reviewed the Z-stack to assess how quickly the biofilm signal disappeared and how much remained in the final 21st stack.

Table S4. List and descriptions of biofilm features calculated by the MicroICS pipeline.

Morphological features:

Feature name in the manuscript	Feature name in the datafile provided by the pipeline	Input	Value normalization	Calculation per _	Description of feature	Threshold
**Counts (threshold < n)***	counts(inten<threshold-SUMMARYCustomAlgos.tsv	raw image	no	layer	The number of bacteria in each layer of the 3D image is determined by first applying the threshold value, then segmenting the individual bacteria, and finally counting them. The raw image or an image whose intensity values have been normalized is used as input. For the “normalized” feature, the counts per layer are normalized as a proportion of the sum of all values * 100 (to obtain percentages) per value.	Range from 0.01 to 0.2 increments for 0.01
**Counts (normalized intensity, threshold < n)***	counts(Norminten<threshold-SUMMARYCustomAlgos.tsv	intensity normalised on raw image	yes	layer		Range from 0.01 to 0.2 increments for 0.01
**Counts (normalized per layer, threshold < n)***	counts(inten<thresholdNormalized-SUMMARYCustomAlgos.tsv	raw image	yes	layer		Range from 0.01 to 0.2 increments for 0.01
**Counts (normalized intensity, normalized per layer, threshold < n)***	counts(Norminten<thresholdNormalized-SUMMARYCustomAlgos.tsv	intensity normalised on raw image	yes	layer		Range from 0.01 to 0.2 increments for 0.01
**Biovolume**	BioVolumeThr-SUMMARYCustomAlgos.tsv	raw image	no	image	The biofilm volume is calculated by counting the number of pixels belonging to the biofilm after thresholding and multiplying this by the voxel size, which represents the physical dimensions of each pixel in height, width, and length (in micrometres). This gives the total volume occupied by the biofilm in the 3D image. Only one volume value is given per image, and the aggregated values remain consistent.	Range from 0.01 to 0.29 increments for 0.01
**GPT volume**	GPTVolume-SUMMARYCustomAlgos.tsv	raw image	no	layer	The GPT volume is similar to the biovolume described above, but here the biovolume is first determined layer by layer after thresholding, and then the values of these layer counts are aggregated for each image.	Fixed value determined for each image
**Normalized GPT volume**	GPTVolumeNormalized-SUMMARYCustomAlgos.tsv	raw image	yes	layer		Fixed value determined for each image
**Substratum relative coverage**	SubstratumRelativeCoverage-SUMMARYCustomAlgos.tsv	raw image	no	image	Percentage of the surface of the well (1st layer of the image) covered with biofilm.	Fixed value determined for each image

**Feature “counts”: Counts are a unique feature that captures variations in counts of bacteria within biofilm by changing the threshold used to detect individual bacteria. It is an ensemble of features known to be correlated with each other and useful in machine learning to capture all aspects of thresholding and counting.*

Statistics-based intensity features:

Feature name in the manuscript	Feature name in the datafile provided by the pipeline	Input type	normalization	Calculation per _	Description of feature	Threshold
**Global mean**	globalMean-SUMMARYDiffGlobal.tsv	raw image	no	image	The average pixel intensity of the entire 3D image reflects the overall brightness of the biofilm. A single value is calculated for each image, whereby all aggregated values are identical.	no
**Layer-wise global maximum****	maxdiffs-SUMMARYDiffGlobal.tsv	raw image	no	image	The average pixel intensities are calculated for each layer of the 3D image, and the difference between the average of each layer and the next is calculated. Only the maximum difference is given, which represents the largest change in brightness between the layers and extends the understanding of the global mean.	no
**Layer-wise global minimum****	mindiffs-SUMMARYDiffGlobal.tsv	raw image	no	image	The difference between the average intensities of consecutive layers is calculated in a similar way, but only the minimum difference is given to highlight areas of little or no change, which may correspond to stable biofilm regions or the background.	no
**Layer-wise global mean****	mdiffs-SUMMARYDiffGlobal.tsv	raw image	no	image	The mean difference between the intensities of successive layers is calculated and reported and reflects the overall uniformity or variability of brightness in the vertical dimension of the biofilm.	no
**/**	eigen-SUMMARYDiffGlobal.tsv	raw image	no	/	A value representing the differences in the overall 3D image is noted but is not currently calculated.	no
**Intensity differences****	diff-SUMMARYCustomAlgos.tsv	raw image	no	layer	The difference between maximum and minimum pixel intensity within each layer is calculated and aggregated, where: (a) the mean aggregation indicates the image contrast, b) the median reflects the typical intensity, c) the standard deviation indicates the variability of the contrast between the layers, and d) the precentiles describe the distribution skewness of the contrast between the layers.	no
**Normalized intensity differences****	diffNormalized-SUMMARYCustomAlgos.tsv	raw image	yes	layer	The differences between the maximum and minimum intensities per layer are normalized as a percentage of the total max-min sum across all layers and then aggregated to characterize the relative contrast distribution.	no
**Mean intensity**	mean-SUMMARYCustomAlgos.tsv	raw image	no	layer	The mean pixel intensity is calculated for each layer and then averaged across the layers, representing the average brightness per layer; higher values indicate denser or brighter regions and vice versa.	no
**Normalized mean intensity**	meanNormalized-SUMMARYCustomAlgos.tsv	raw image	yes	layer	The mean intensity on each layer is normalized as a percentage of the total sum of mean intensities per image before averaging across layers.	no
**Median intensity**	med-SUMMARYCustomAlgos.tsv	raw image	no	layer	The median pixel intensity is calculated for each layer, and then the median values are aggregated across all layers.	no
**Normalized median intensity**	medNormalized-SUMMARYCustomAlgos.tsv	raw image	yes	layer	The median intensity per layer is normalized as a percentage of the total sum of the median intensities per image and then aggregated across layers.	no
**Maximum intensity**	max-SUMMARYCustomAlgos.tsv	raw image	no	layer	The maximum pixel intensity is calculated per layer and aggregated, highlighting localized bright spots such as clumps or active cells.	no

Feature name in the manuscript	Feature name in the datafile provided by the pipeline	Input type	normalization	Calculation per _	Description of feature	Threshold
**Normalized maximum intensity**	maxNormalized-SUMMARYCustomAlgos.tsv	raw image	yes	layer	The maximum intensity per layer is normalized as a percentage of the total maximum per image and then aggregated.	no
**Minimum intensity**	min-SUMMARYCustomAlgos.tsv	raw image	no	layer	The minimum pixel intensity per layer is calculated and aggregated to capture the darkest or least dense biofilm regions; in combination with other statistics, it shows biofilm gaps, heterogeneity and spatial structure.	no
**Normalized minimum intensity**	minNormalized-SUMMARYCustomAlgos.tsv	raw image	yes	layer	The minimum intensity per layer is normalized as a percentage of the total minimum sum per image before aggregation.	no
**Standard deviation of intensity**	std-SUMMARYCustomAlgos.tsv	raw image	no	layer	The standard deviation of pixel intensities per layer is calculated and aggregated; low values indicate uniform intensity (low contrast), while high values indicate high contrast and high variability.	no
**Normalized standard deviation of intensity**	stdNormalized-SUMMARYCustomAlgos.tsv	raw image	yes	layer	The standard deviation per layer is normalized as a percentage of the total standard deviation sum per image and then aggregated.	no

***Layer wise features:*
Layer wise features capture how much the intensity fluctuates between the layers and how features differ across the volume.

Texture features:

Feature name in the manuscript	Feature name in the datafile provided by the pipeline	Input type	normalization	Calculation per _	Description of feature	Threshold
**Void fraction (min Prop)**	minProp-SUMMARYCustomAlgos.tsv	raw image	no	layer	The number of pixels with intensity below the average pixel intensity is counted for each image. Min prop refers to dark areas, such as empty regions not occupied by biofilm.	no
**Normalized void fraction**	minPropNormalized-SUMMARYCustomAlgos.tsv	raw image	yes	layer		no
**Homogeneity**	Homogenity-SUMMARYCustomAlgos.tsv	raw image	no	image	Homogeneity is calculated using the co-occurrence matrix, which captures the distribution of intensity changes between neighbouring pixels in the X, Y and Z directions (after Beyenal et al. 2004, doi: 10.1016/j.mimet.2004.08.003). It measures how similar neighbouring image objects are, with higher values indicating greater uniformity. One value is given per image, and the aggregated values are identical.	no
**Thickness**	ThicknessThreshold=0.01-SUMMARYCustomAlgos.tsv	raw image	no	image	Pixel intensities are tracked vertically from the first image layer, recording for each pixel the height at which the intensity drops to zero. The average of these heights is reported as a single image value using the same aggregation.	Range from 0.01 to 0.29 increments for 0.01
**Roughnes**	RoughnessThreshold=threshold-SUMMARYCustomAlgos.tsv	raw image	no	image	The average height difference between neighbouring pixels at a given layer thickness is calculated and reflects the roughness of the biofilm surface. One value per image is given, with uniform aggregation.	Range from 0.01 to 0.29 increments for 0.01
**Horizontal spreading**	SpreadingHorizontal-SUMMARYCustomAlgos.tsv	raw image	no	image	Three distributions (Dx, Dy, Dz) of the x, y, and z coordinates of the biofilm pixels are created, as described by Mueller et al. (2006, doi: 10.1186/1472-6785-6-1), and the variances for each axis are calculated. Horizontal spreading is calculated by summing up variances in x and y. Vertical spreading (in z direction) equals the variance of z. Total spreading (in z direction) is calculated by summing up variances in x, y and z.	no
**Total spreading**	SpreadingTotal-SUMMARYCustomAlgos.tsv	raw image	no	image		no
**Vertical spreading**	SpreadingVertical-SUMMARYCustomAlgos.tsv	raw image	no	image		no

Feature name in the manuscript	Feature name in the datafile provided by the pipeline	Input type	normalization	Calculation per _	Description of feature	Threshold
**GPT fractal dimension**	GPTFractalDim-SUMMARYCustomAlgos.tsv	raw image	no	layer	The fractal dimension reflects the complexity and irregularity of the surface or boundary of the biofilm and quantifies this complexity at different scales; higher values indicate more complex and branched structures rather than smooth shapes.	no
**Normalized GPT fractal dimension**	GPTFractalDimNormalized-SUMMARYCustomAlgos.tsv	raw image	yes	layer		no
**GPT homogeneity**	GPTHomogeneity-SUMMARYCustomAlgos.tsv	raw image	no	image	This feature is calculated similarly to non-GPT homogeneity, but is first computed for each layer and then aggregated for each image; higher homogeneity indicates a more uniform and consistent texture.	no

The following features were calculated by Graycomatrix method, which constructs a grayscale co-occurrence matrix (GLCM) by calculating how many times a pixel with intensity value (grayscale) *i* occurs in a given spatial relationship to a pixel with value *j*.

Feature name in the manuscript	Feature name in the datafile provided by the pipeline	Input type	normalization	Calculation per _	Description of feature	Threshold
**GPT contrast**	GPTContrast-SUMMARYCustomAlgos.tsv	raw image	no	image	Contrast emphasises the difference between grey-level values. Larger differences in intensity contribute more to the sum, so a higher contrast value indicates greater variability in the intensities of neighbouring pixels.	no
**GPT correlation**	GPTCorrelation-SUMMARYCustomAlgos.tsv	raw image	no	image	The correlation value is calculated similarly to the Pearson correlation coefficient, using the correlations between values in the graycomatrix. High correlations indicate that certain grey-level pairs occur in a predictable manner.	no
**GPT dissimilarity**	GPTDissimilarity-SUMMARYCustomAlgos.tsv	raw image	no	image	Similar to contrast, but it is calculated from absolute differences instead of a squared difference. Dissimilarity also captures local variations in gray levels, but its contribution grows more linearly compared to contrast's squared term. Dissimilarity is opposite to homogeneity.	no
**GPT energy**	GPTEnergy-SUMMARYCustomAlgos.tsv	raw image	no	image	Energy is another way to express the uniformity or orderliness in the texture. It is often used because it is on a [0,1] scale if P is normalized.	no

Figure S3. UMAP projections of all features calculated from the images for each individual biological replicate (experiments performed on different days) for the eight strains studied. This illustrates the increasing complexity when adding data from multiple replicates, which are nevertheless necessary to capture biological variation.

a. 1st biological replicate b. 2nd biological replicate c. 3rd biological replicate

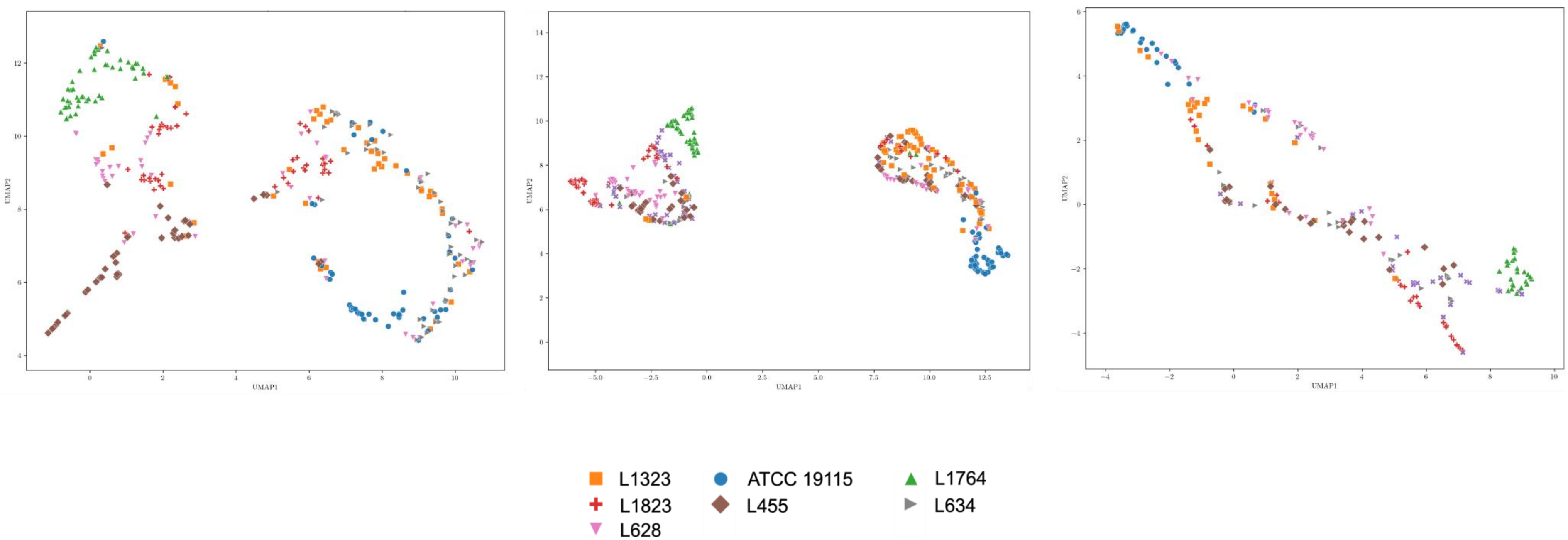

Table S5. The 81 most influential features for *L. monocytogenes* strain classification (identity task) were selected using random forest-based feature importance and ablation analysis. Features overlapping with those critical for predicting experimental date (batch effect) from biofilm features suggest potential confounding by temporal effects or batch recurrence. Such features may therefore be less reliable for biological interpretation, as they could reflect methodological artefacts rather than intrinsic strain differences.

score	feature name	aggregation type	found among the top-ranking features according to the date	calculated for layer or image
0.08112858	GPT fractal dimension	max	yes	layer
0.06571528	counts (normalized intensity, normalized per layer, threshold < 0.11)	max	no	layer
0.05874927	minimum intensity	var	no	layer
0.05795771	minimum intensity	std	no	layer
0.03175054	counts (normalized intensity, normalized per layer, threshold < 0.14)	max	no	layer
0.02784039	min prop	min	yes	layer
0.02188584	counts (threshold < 0.06)	median	no	layer
0.02038614	GPT fractal dimension	q90	no	layer
0.01730499	counts (normalized intensity, normalized per layer, threshold < 0.13)	max	no	layer
0.01443651	counts (normalized intensity, normalized per layer, threshold <0.12)	max	no	layer
0.01389953	counts (threshold < 0.08)	median	no	layer
0.01328647	counts (normalized intensity, threshold < 0.22)	max	no	layer
0.01243395	counts (normalized intensity, threshold < 0.05)	q10	no	layer
0.01223955	min prop	q10	no	layer
0.0087634	counts (intensity < 0.069)	median	no	layer
0.00790731	normalized GPT fractal dimension	var	yes	layer

score	feature name	aggregation type	found among the top-ranking features according to the date	calculated for layer or image
0.00757033	normalized GPT fractal dimension	q10	no	layer
0.00696023	counts (normalized intensity, threshold < 0.12)	max	no	layer
0.00693635	normalized GPT fractal dimension	std	yes	layer
0.00630384	Intensity differences	median	no	layer
0.00591981	normalized standard deviation of intensity	min	no	layer
0.00521997	standard deviation of intensity	var	no	layer
0.00516137	normalized standard deviation of intensity	q25	no	layer
0.00485178	counts (normalized intensity, threshold < 0.05)	min	yes	layer
0.0045148	counts (normalized intensity, normalized per layer, threshold < 0.29)	median	no	layer
0.00445399	GPT volume	q90	yes	layer
0.0042627	counts (threshold < 0.1)	q25	no	layer
0.00419321	counts (normalized intensity, threshold < 0.29)	std	no	layer
0.00410091	standard deviation of intensity	std	no	layer
0.00393888	counts (normalized intensity, threshold < 0.21)	max	no	layer
0.00382617	counts (normalized intensity, threshold < 0.04)	min	no	layer
0.00362833	counts (threshold < 0.07)	max	no	layer
0.00356759	counts (threshold < 0.07)	var	no	layer
0.00356677	counts (normalized intensity, threshold < 0.29)	var	no	layer

score	feature name	aggregation type	found among the top-ranking features according to the date	calculated for layer or image
0.00352052	Intensity differences	q75	no	layer
0.003445	counts (threshold < 0.07)	std	no	layer
0.00341029	counts (normalized intensity, threshold < 0.13)	max	no	layer
0.00279609	counts (normalized intensity, normalized per layer, threshold < 0.23)	median	no	layer
0.00277509	counts (normalized intensity, threshold < 0.23)	max	no	layer
0.00248325	maximum intensity	median	no	layer
0.00247693	GPT fractal dimension	std	no	layer
0.00246749	counts (threshold < 0.06)	q90	yes	layer
0.00244786	min prop	q25	no	layer
0.00223703	counts (normalized intensity, threshold < 0.26)	q75	no	layer
0.00218651	counts (normalized intensity, normalized per layer, threshold < 0.14)	q90	no	layer
0.00210787	Normalized standard deviation of intensity	q75	no	layer
0.00210103	counts (threshold < 0.06)	var	no	layer
0.00208782	normalized median intensity	std	no	layer
0.00205966	counts (normalized intensity, normalized per layer, threshold < 0.1)	min	no	layer
0.00202265	counts (threshold < 0.06)	q75	yes	layer
0.00201584	counts (normalized per layer, threshold < 0.06)	q90	no	layer
0.00199038	counts (normalized intensity, threshold 0.1)	max	yes	layer

score	feature name	aggregation type	found among the top-ranking features according to the date	calculated for layer or image
0.00195483	normalized median intensity	var	no	layer
0.00193883	normalized GPT fractal dimension	q75	yes	layer
0.00189067	normalized min prop	max	no	layer
0.00186792	Intensity differences	mean	no	layer
0.00184007	normalized GPT volume	q90	no	layer
0.00178068	counts (normalized intensity, normalized per layer, threshold < 0.11)	min	no	layer
0.00176622	minimum intensity	q90	no	layer
0.00174161	GPT volume	median	no	layer
0.0017407	GPT correlation	q10	no	layer
0.00173914	counts (normalized intensity, normalized per layer, threshold < 0.1)	max	no	layer
0.00172901	counts (normalized per layer, threshold < 0.06)	max	no	layer
0.00172085	counts (threshold < 0.09)	median	no	layer
0.00171266	GPT fractal dimension (normalized per layer)	q25	no	layer
0.00171073	counts (normalized intensity, normalized per layer, threshold < 0.03)	max	yes	layer
0.00168393	normalized standard deviation of intensity	q10	no	layer
0.0016726	normalized min Prop	q90	no	layer
0.00165754	GPT correlation	q25	no	layer
0.00163483	counts (normalized intensity, normalized per layer, threshold 0.06)	q10	no	layer

score	feature name	aggregation type	found among the top-ranking features according to the date	calculated for layer or image
0.00160801	counts (normalized intensity, threshold < 0.2)	max	no	layer
0.00160775	counts (normalized intensity, normalized per layer, threshold < 0.15)	max	no	layer
0.0015765	normalized minimum intensity	q25	no	layer
0.0015559	counts (normalized intensity, normalized per layer, threshold < 0.29)	q90	no	layer
0.00155201	counts (threshold < 0.05)	q90	yes	layer
0.00153526	counts (normalized intensity, threshold < 0.14)	max	yes	layer
0.00152136	normalized median intensity	max	no	layer
0.00147419	Intensity differences	q90	no	layer
0.00145549	counts (threshold < 0.07)	min	no	layer
0.00140754	counts (normalized intensity, normalized per layer, threshold < 0.24)	median	no	layer
0.00139902	GPT correlation	q90	no	layer

Figure S4. Results of pairwise comparison using the Games-Howell post hoc test of the key features distinguishing between strains (task identity) in relation to the strains, illustrating how different features and aggregation types contribute to the classification. The feature names in the diagrams are abbreviated and the full name of the feature is given above the diagram. The aggregation types are abbreviated as follows: min – minimum, q10 – 10th percentile q25 – 25th percentile, med – medium, q75 – 75th percentile, q90 – 90th percentile, max – maximum, std – standard deviation, var – variance. Statistical significance is indicated by letters in the bars; if two bars have different letters, the values are significantly different. If there are no significant differences between two values, they are given the same letter.

GPT fractal dimension (F66)

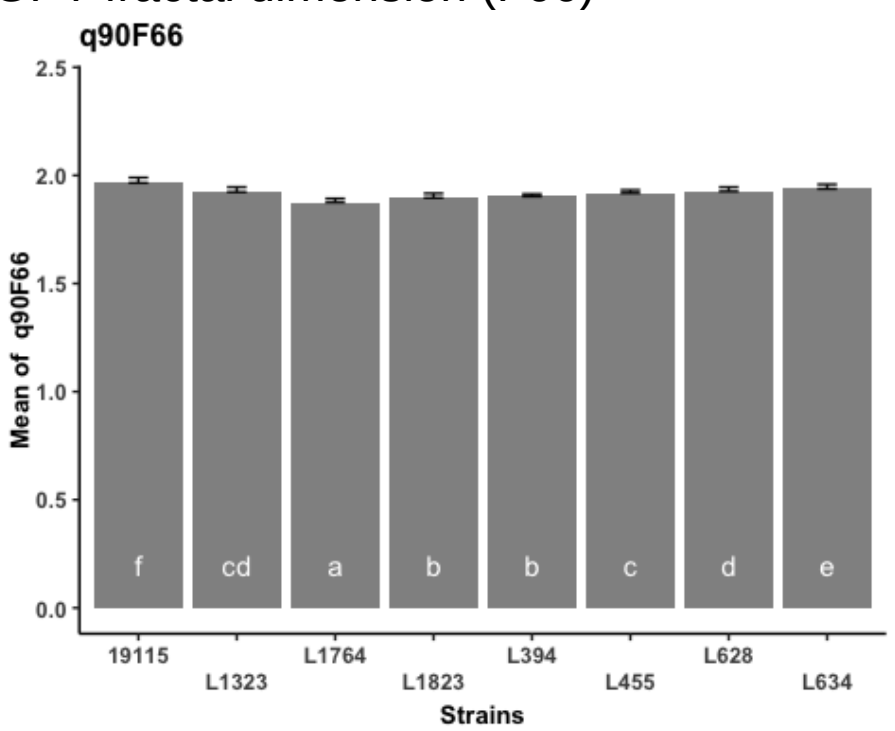

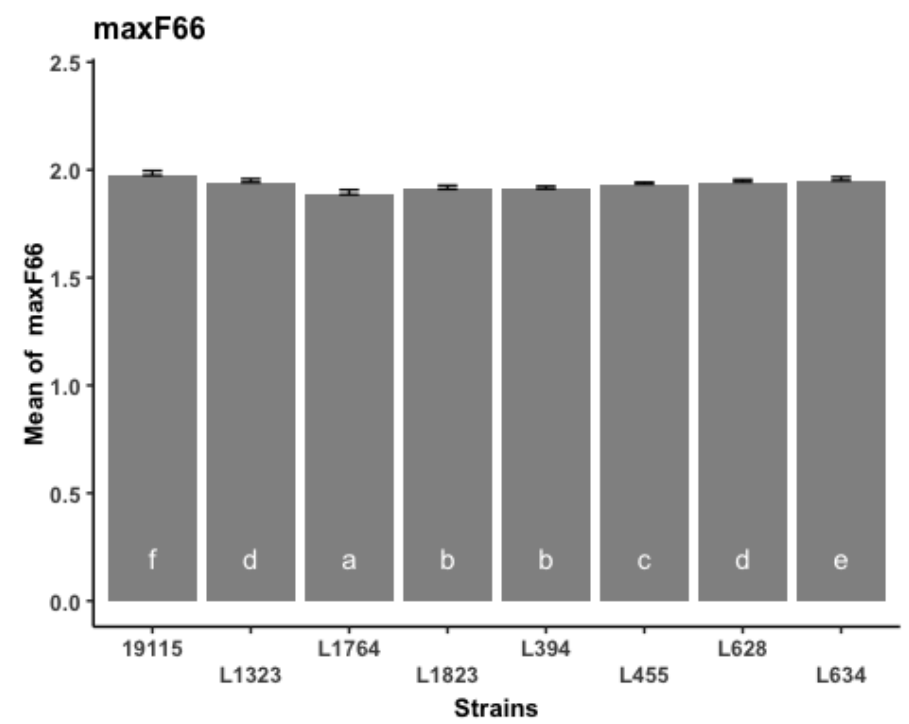

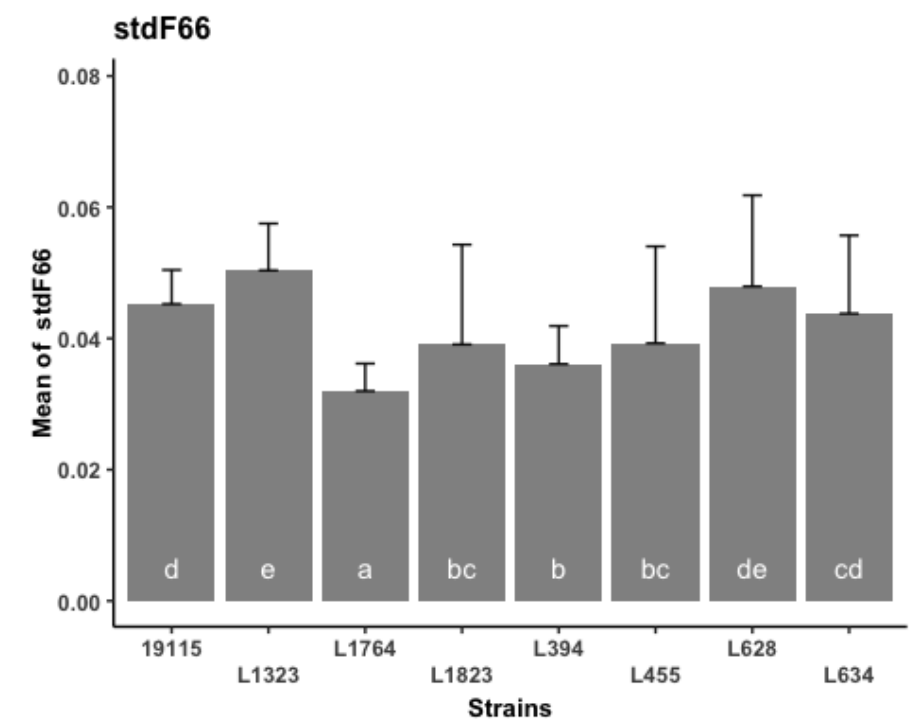

normalized GPT fractal dimension (F67)

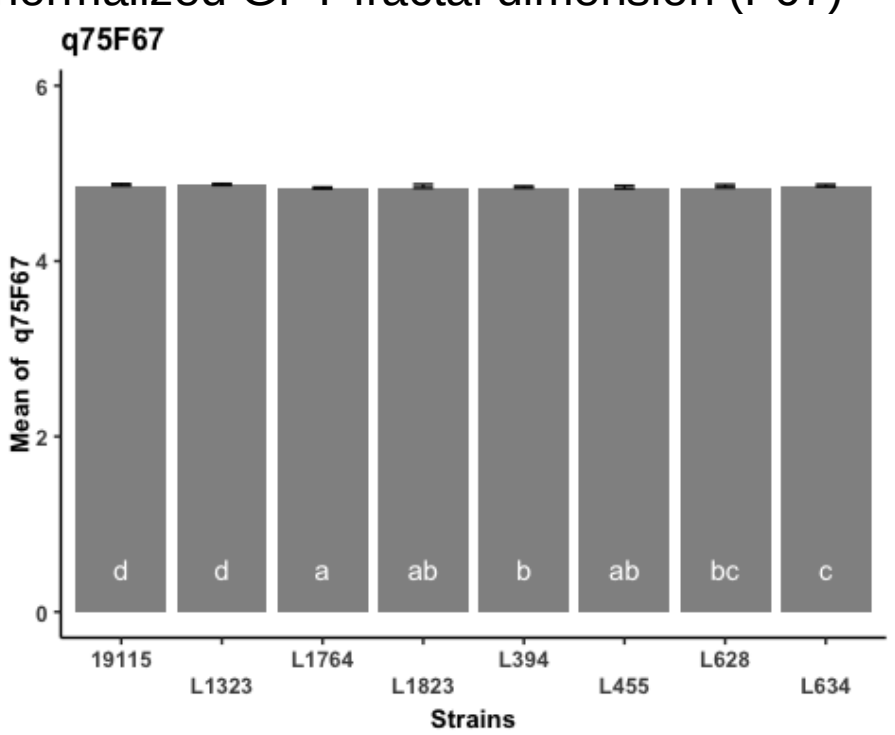

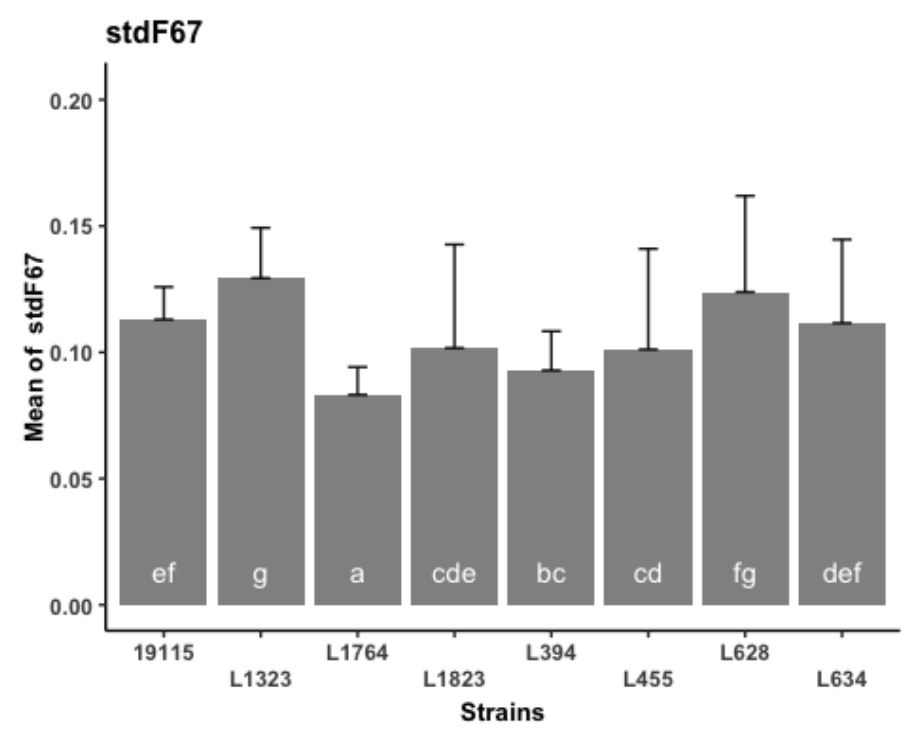

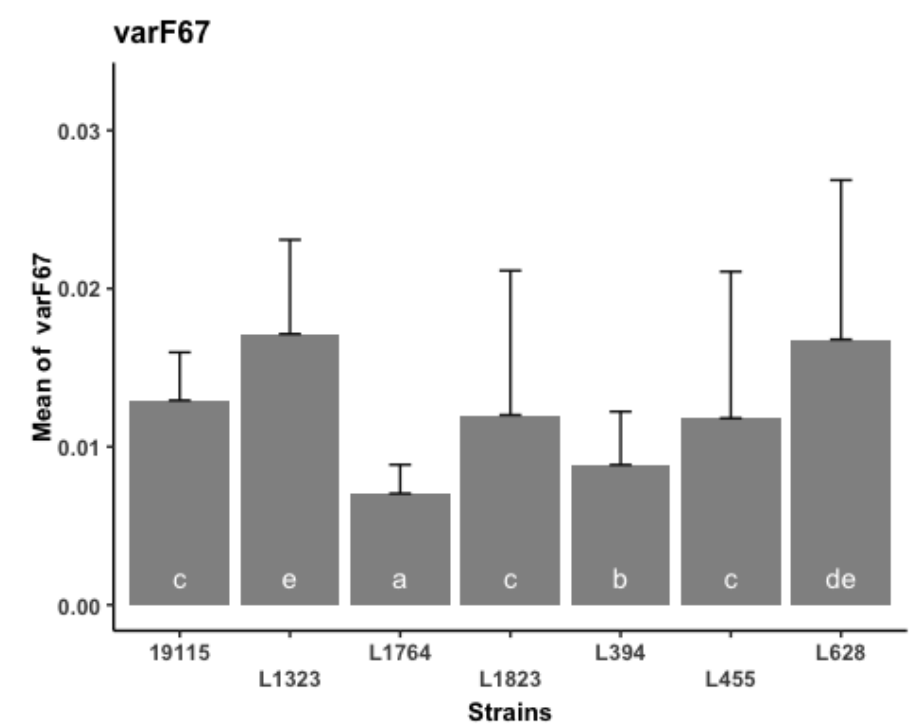

GPT volume (F70)

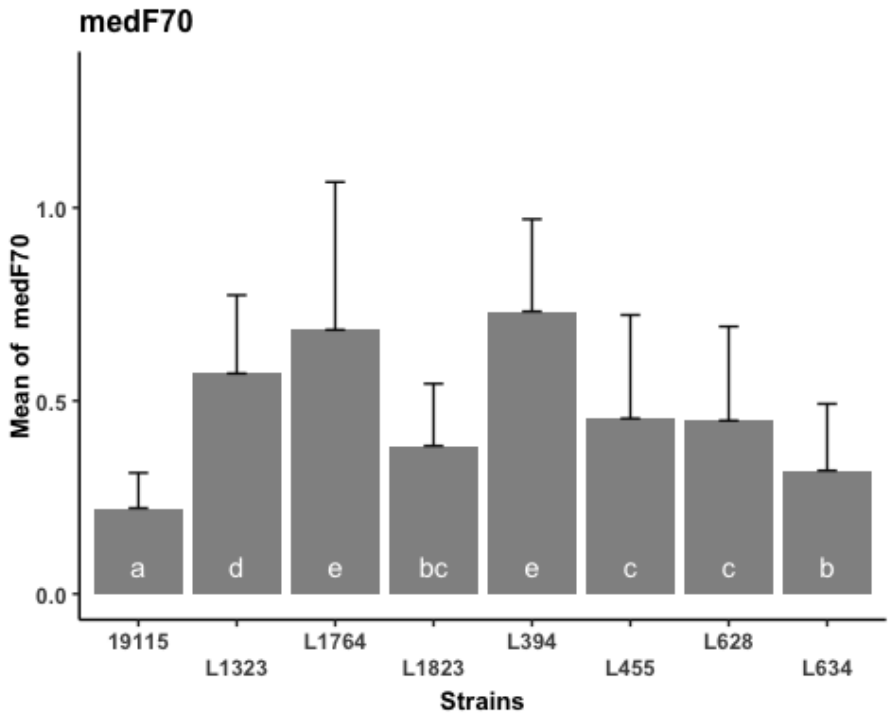

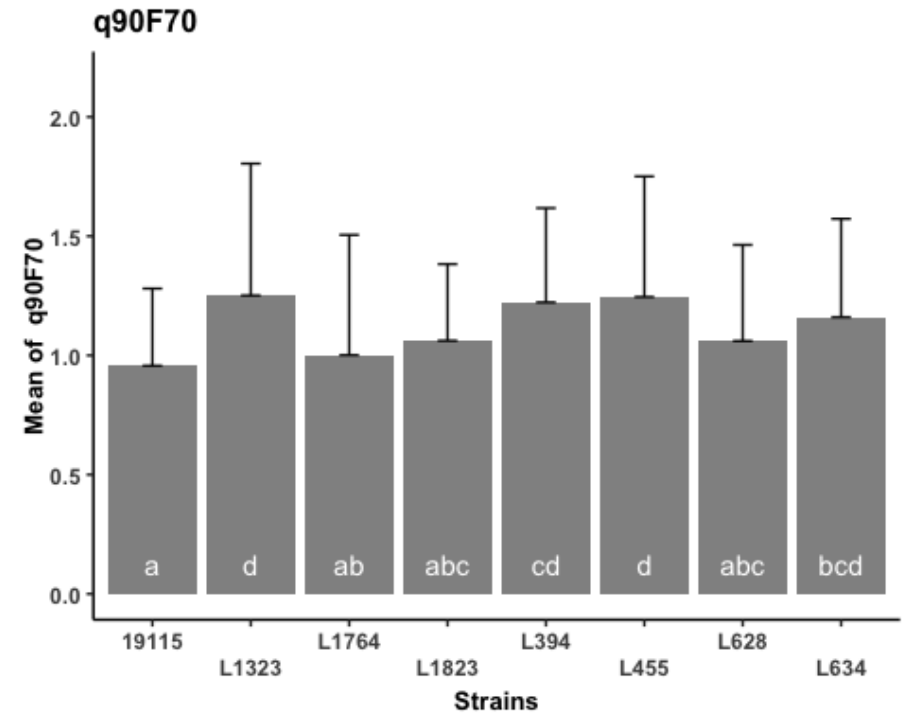

normalized GPT volume (F71)

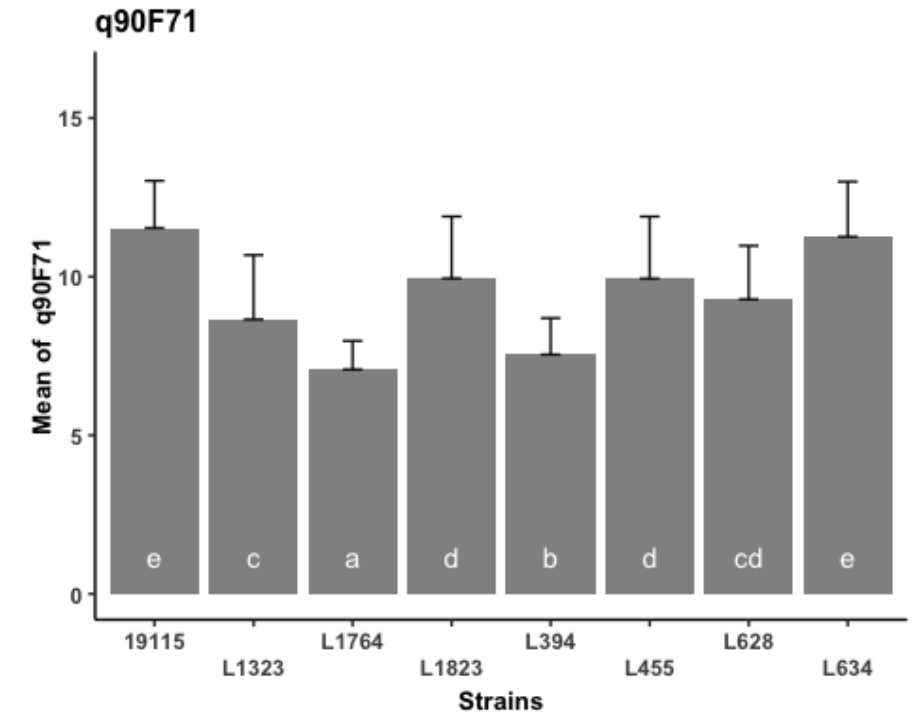

counts (normalized intensity, normalized per layer, threshold < 0.03) (F144)

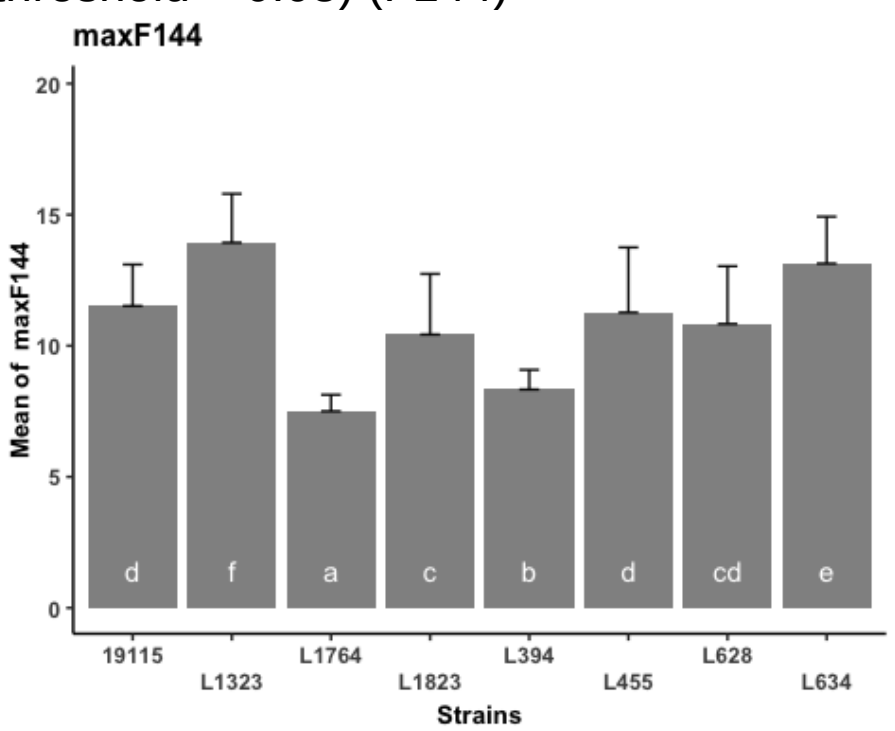

counts (normalized intensity, threshold < 0.04) (F145)

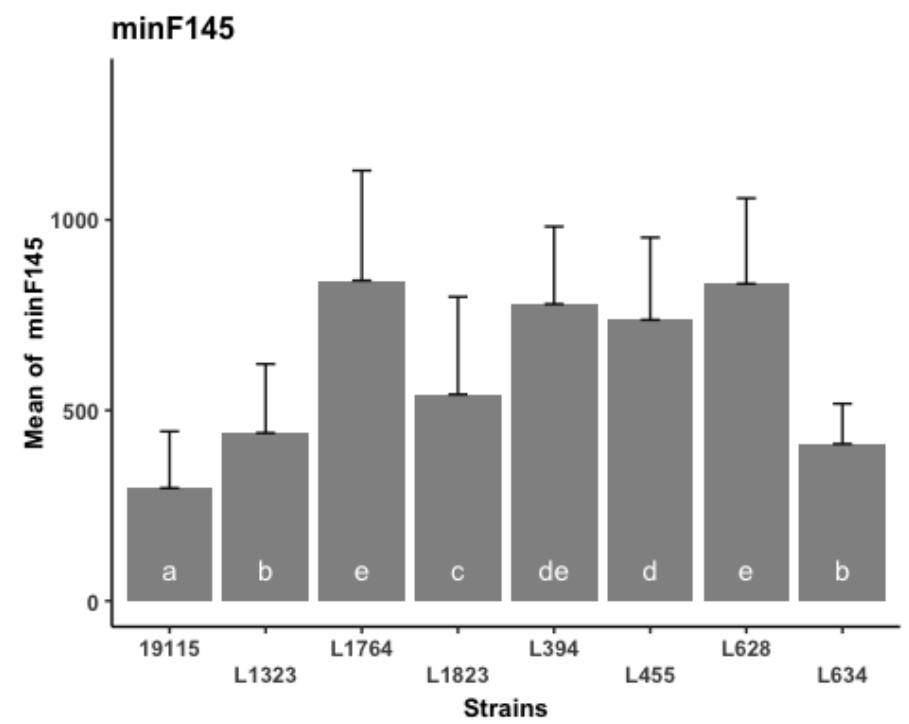

counts (normalized intensity, threshold < 0.05) (F147)

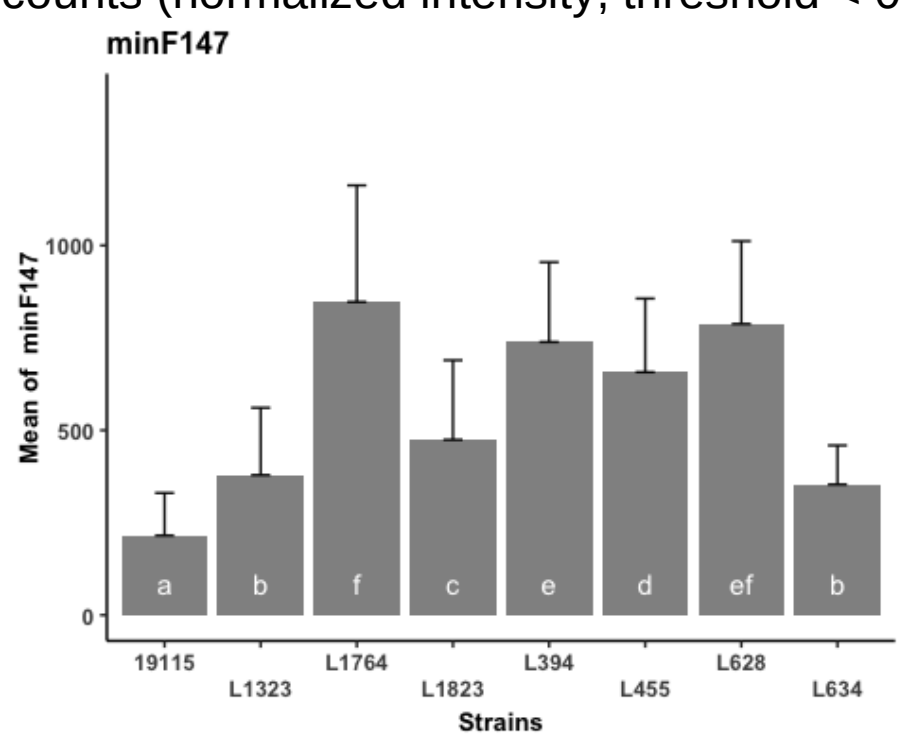

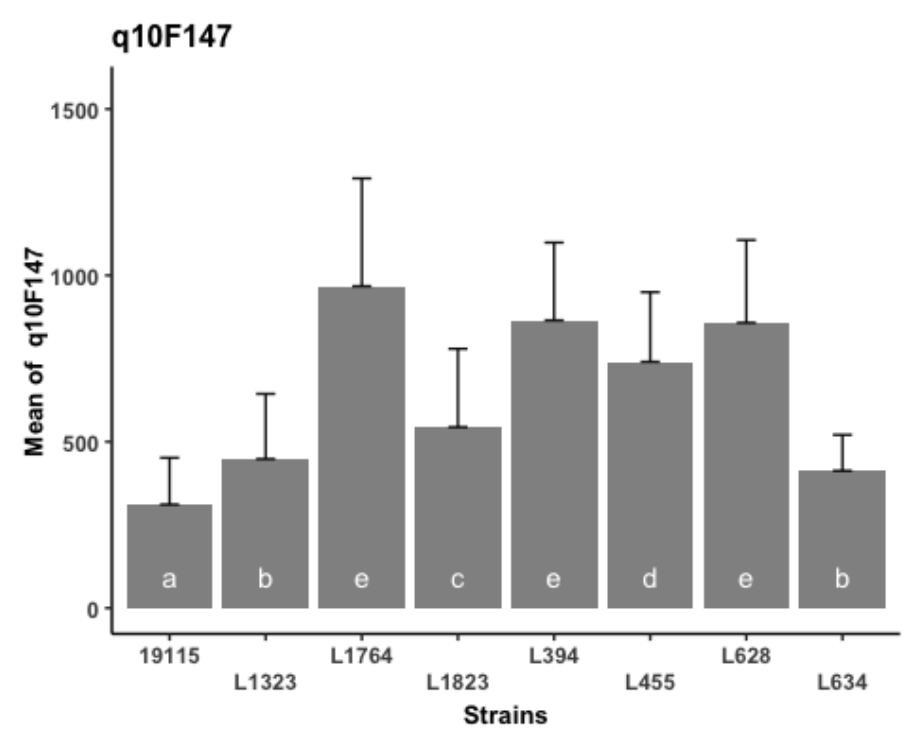

counts (normalized intensity, normalized per layer, threshold 0.06) (F150)

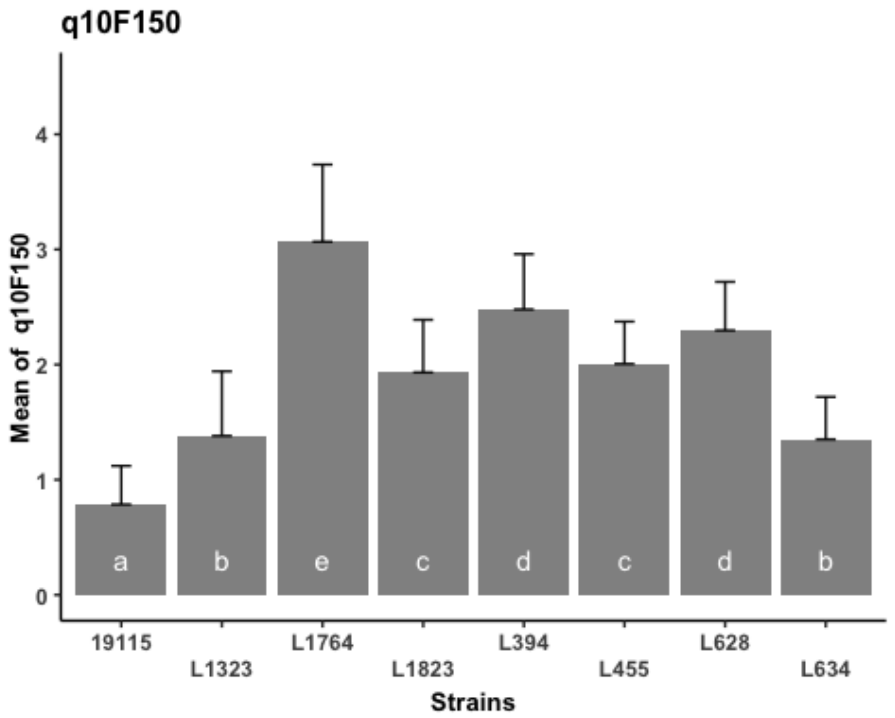

counts (normalized intensity, threshold 0.1) (F156)

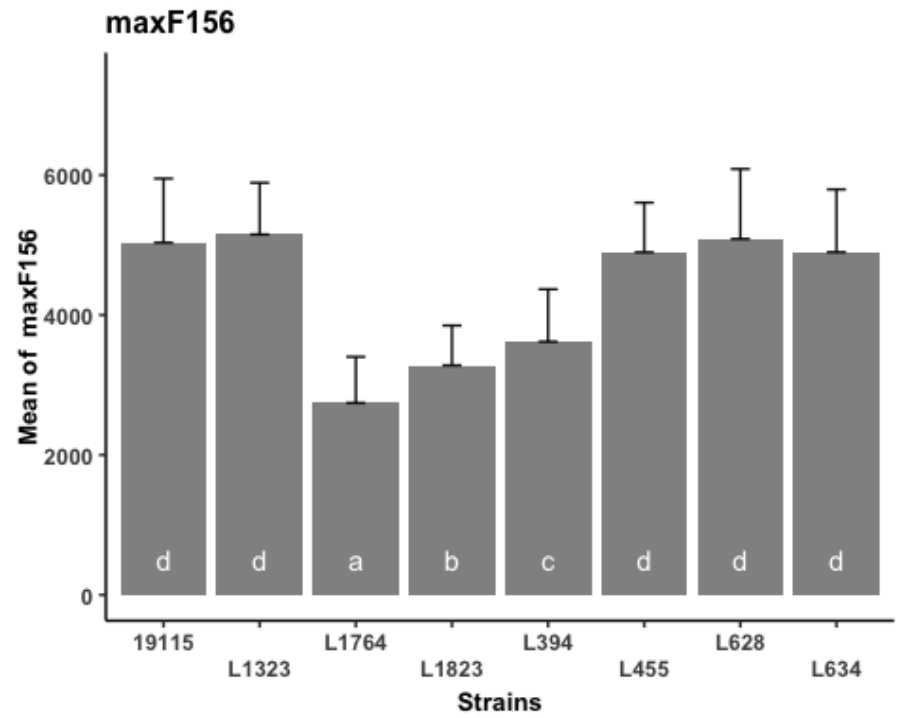

counts (normalized intensity, normalized per layer, threshold < 0.1) (F157)

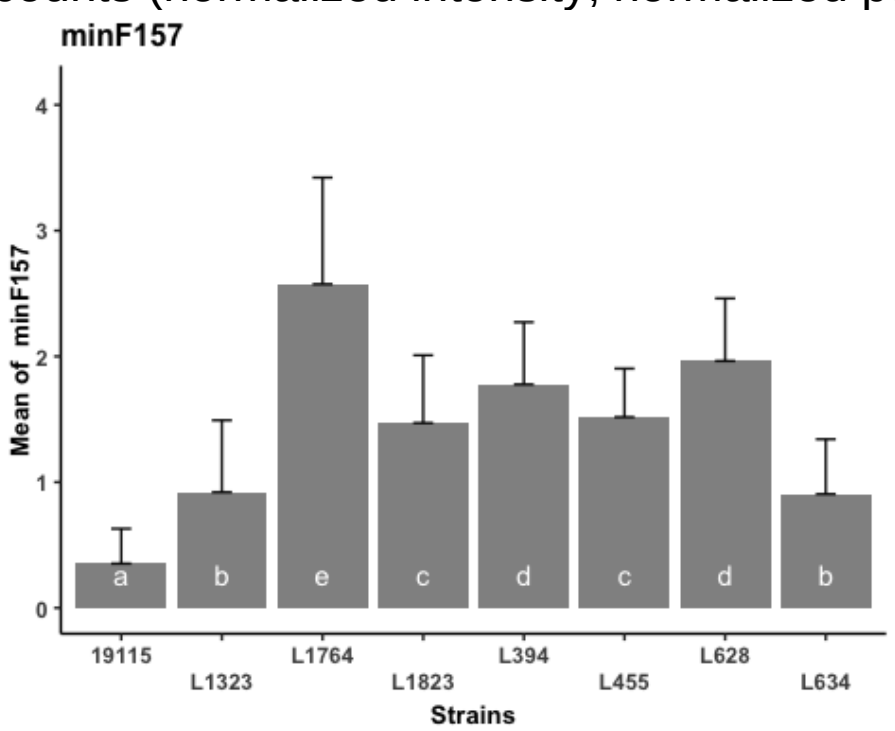

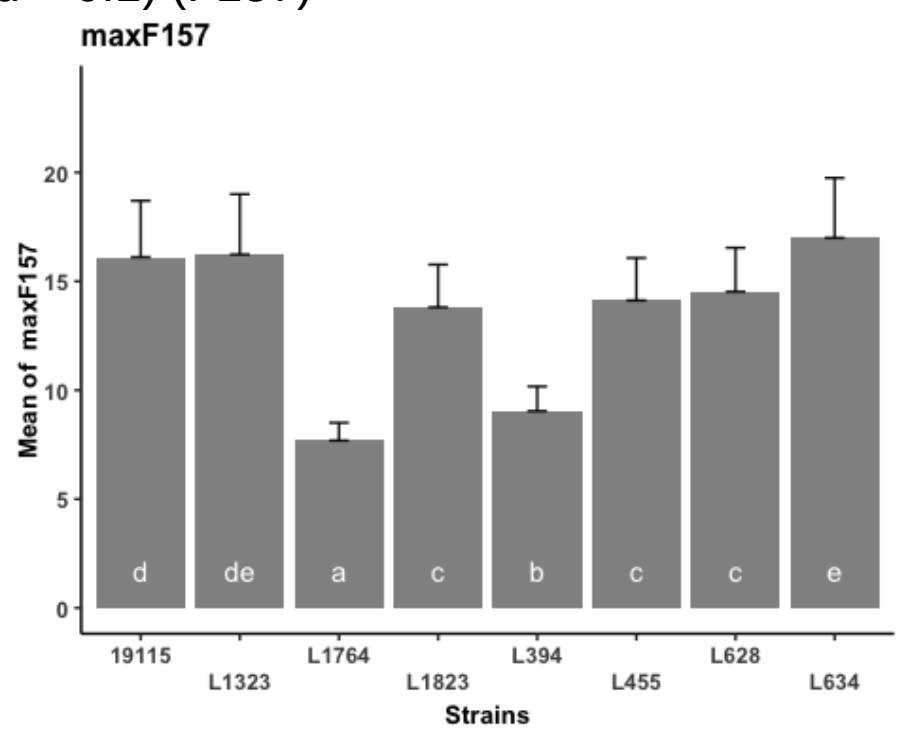

counts (normalized intensity, normalized per layer, threshold < 0.11) (F160)

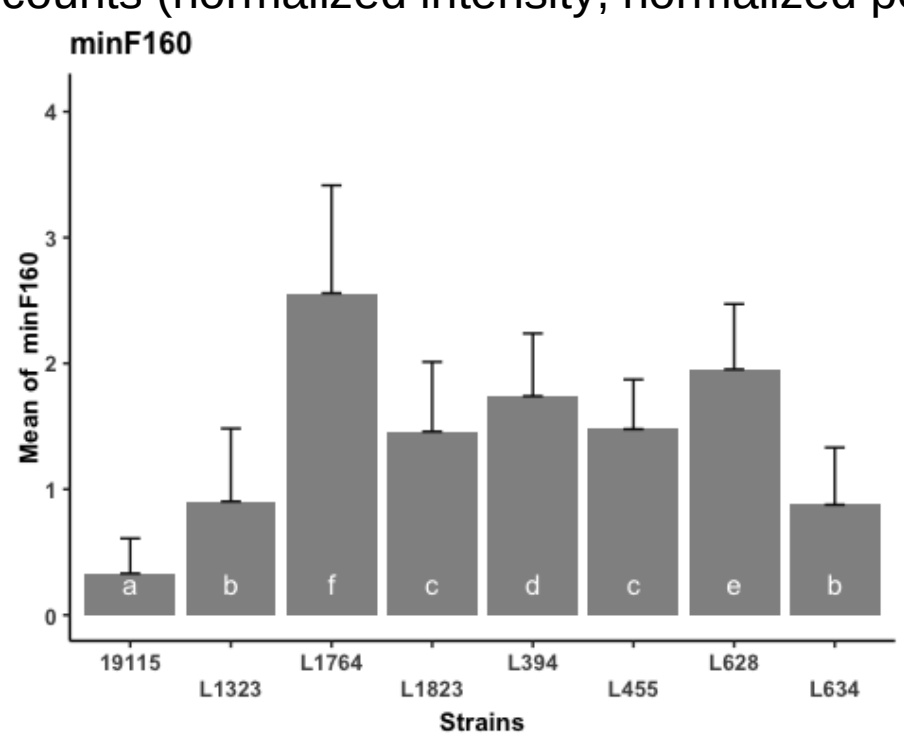

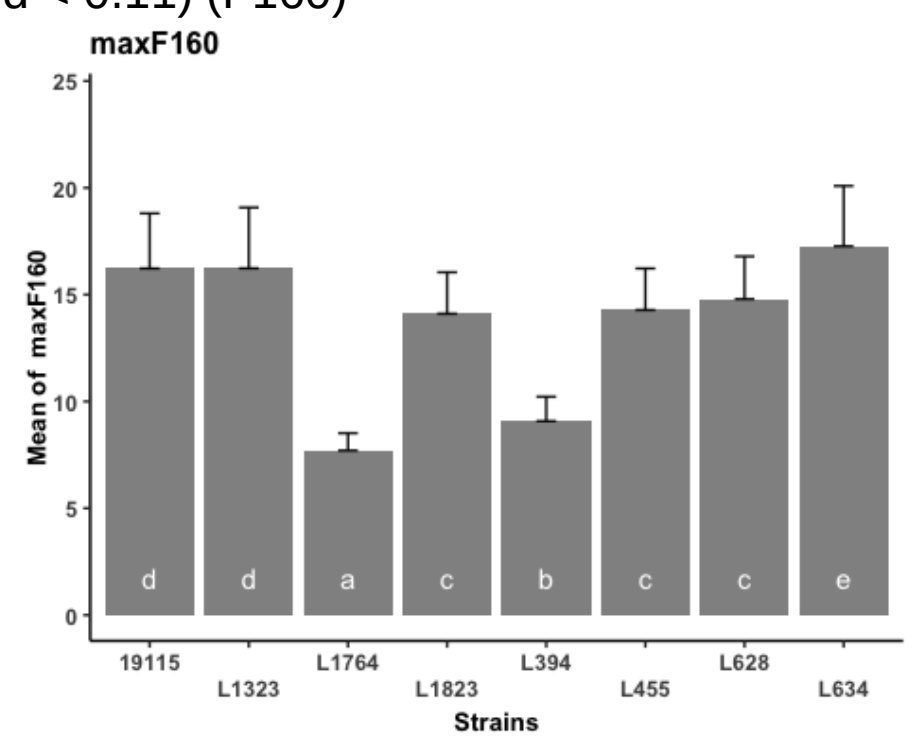

counts (normalized intensity, threshold < 0.12) (F161)

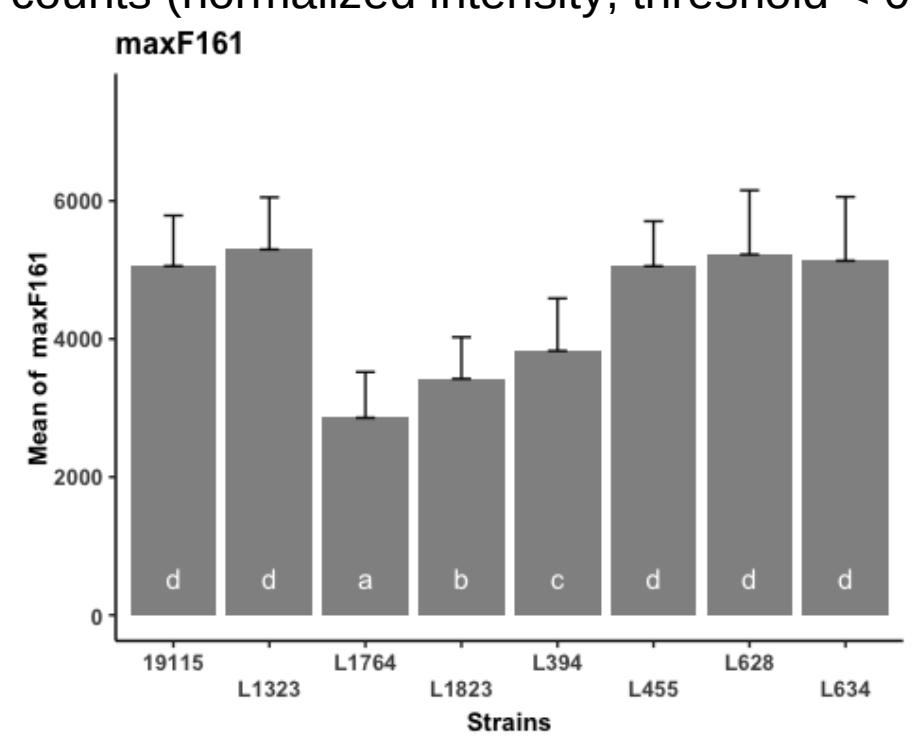

counts (normalized intensity, normalized per layer, threshold <0.12) (F162)

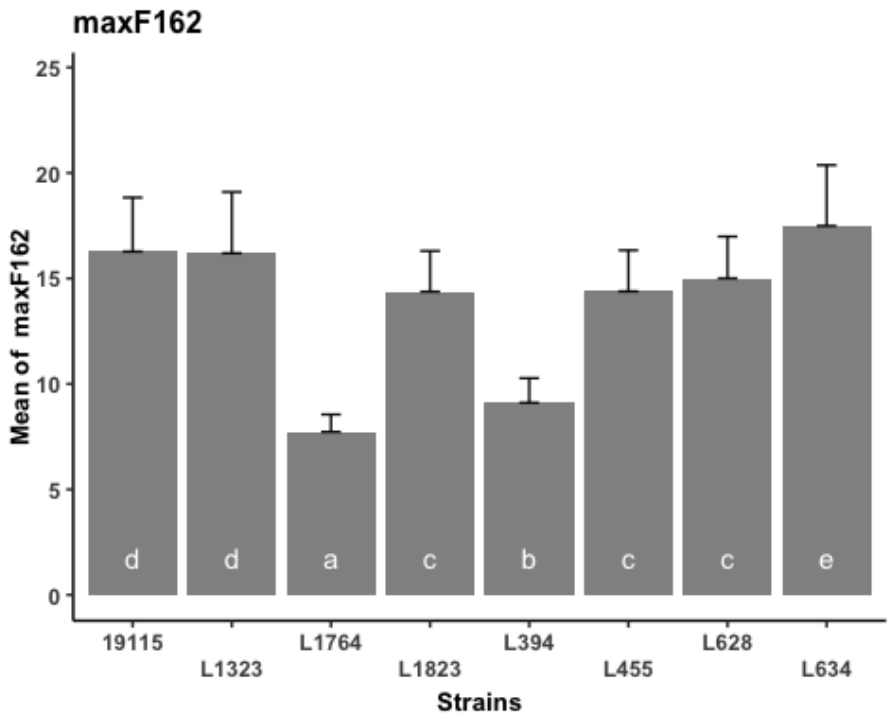

counts (normalized intensity, threshold < 0.13) (F163)

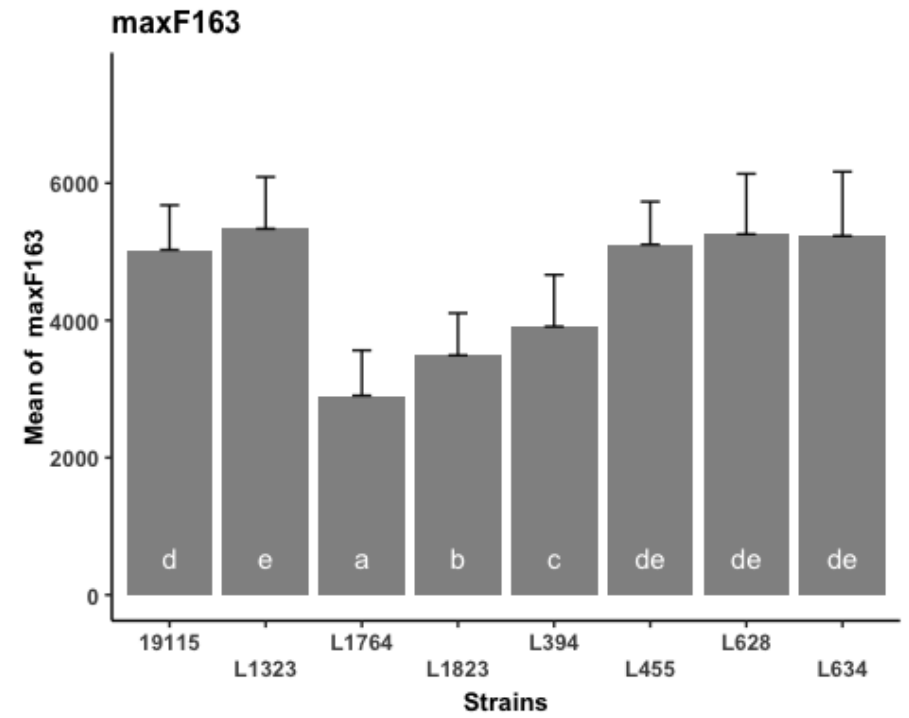

counts (normalized intensity, normalized per layer, threshold < 0.13) (F164)

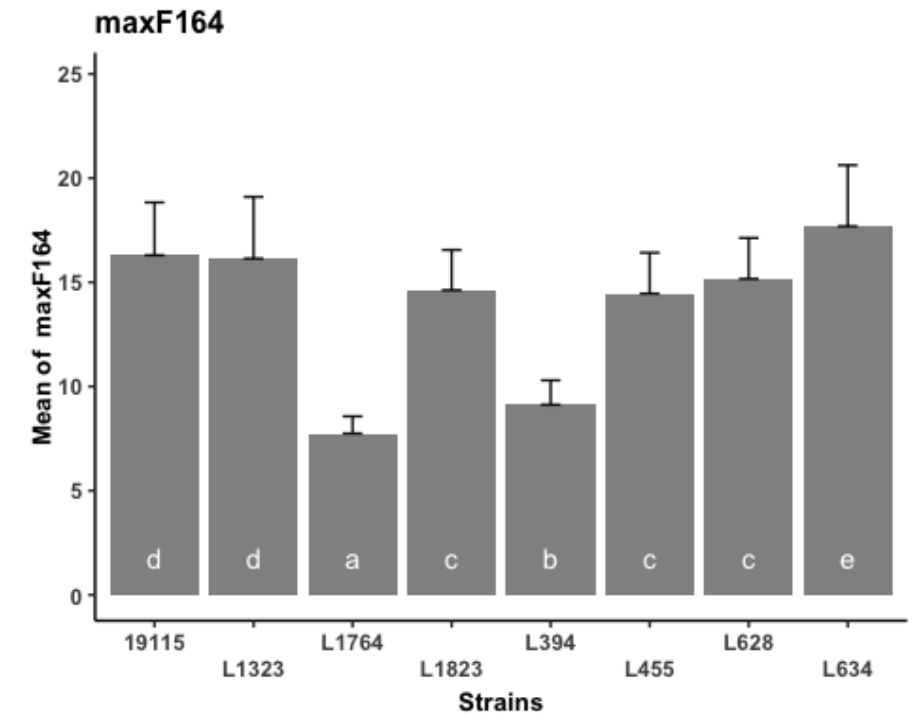

counts (normalized intensity, threshold < 0.14) (F165)

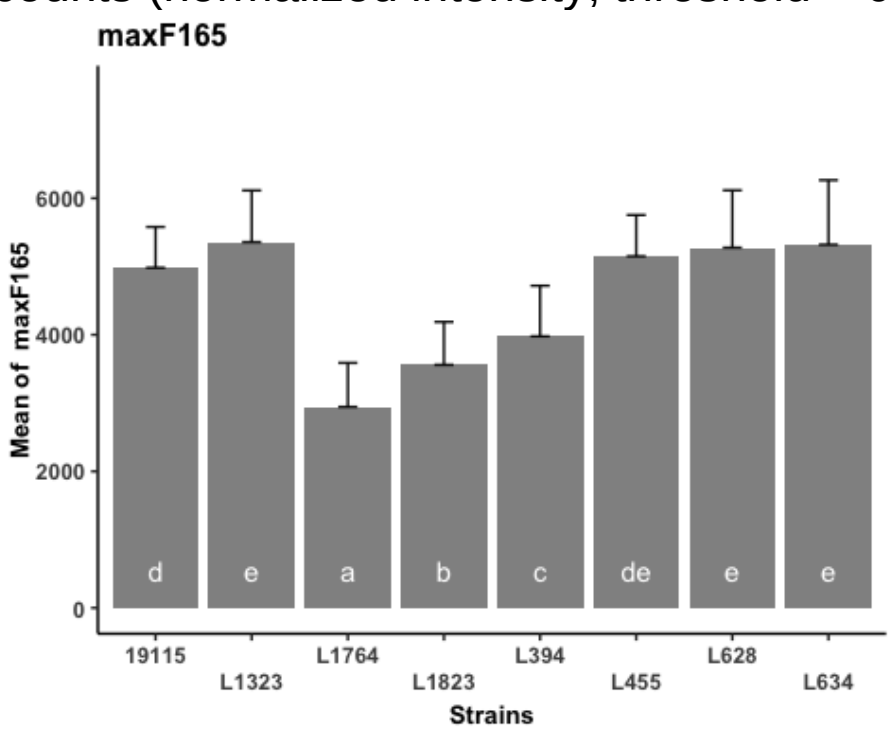

counts (normalized intensity, normalized per layer, threshold < 0.14) (F166)

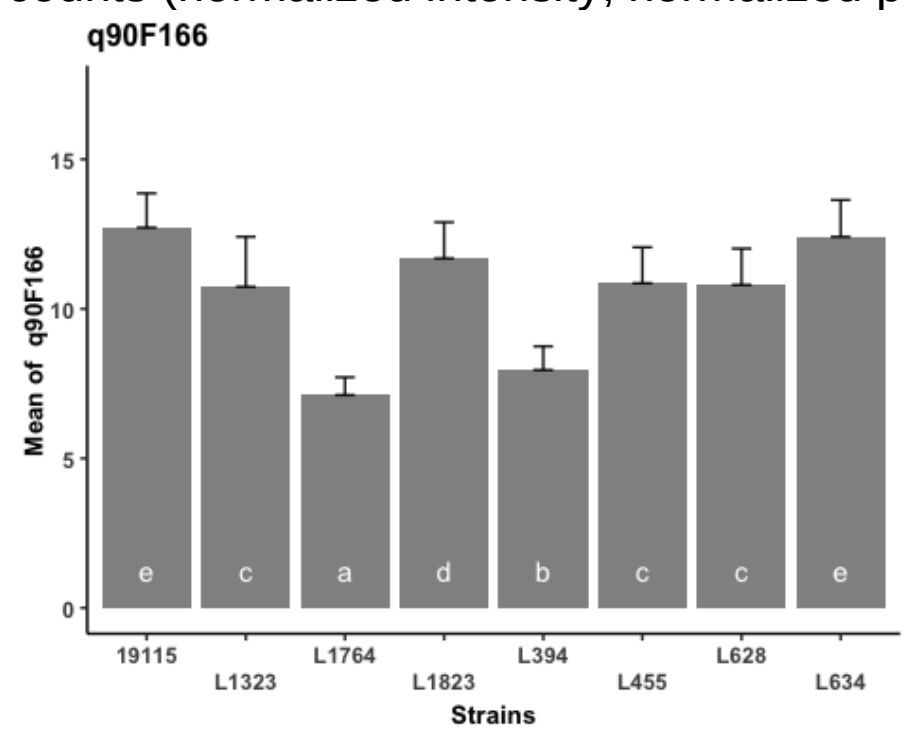

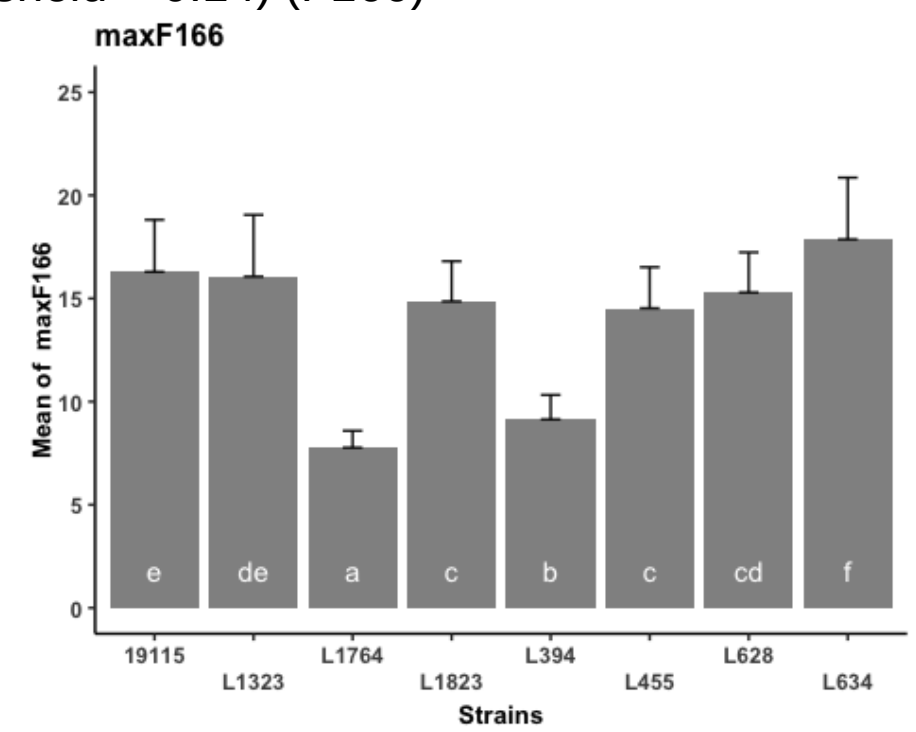

counts (normalized intensity, normalized per layer, threshold < 0.15) (F168)

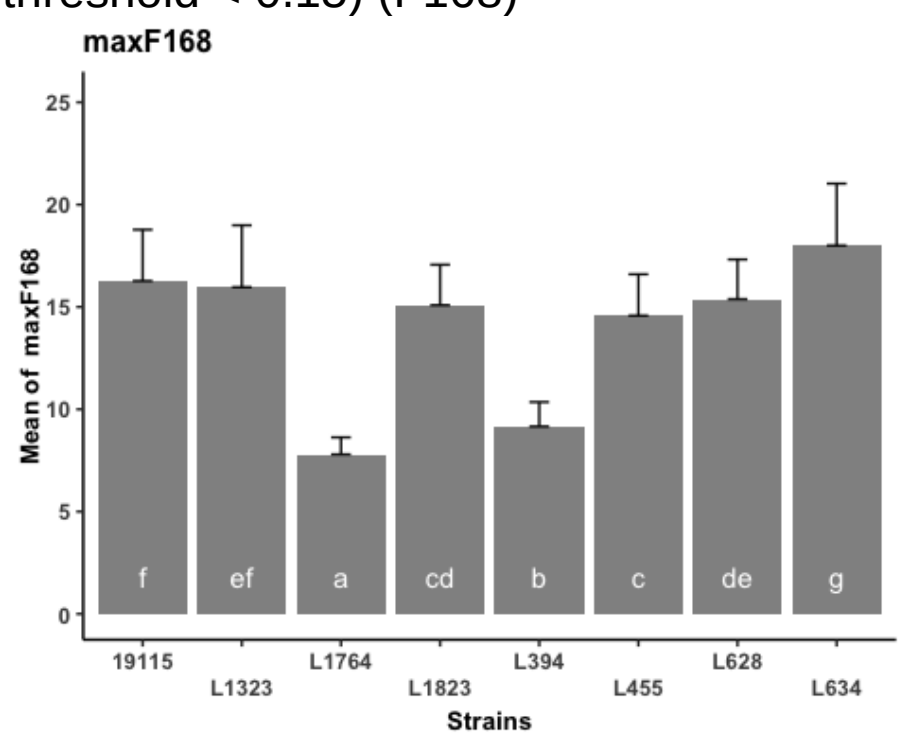

counts (normalized intensity, threshold < 0.2) (F177)

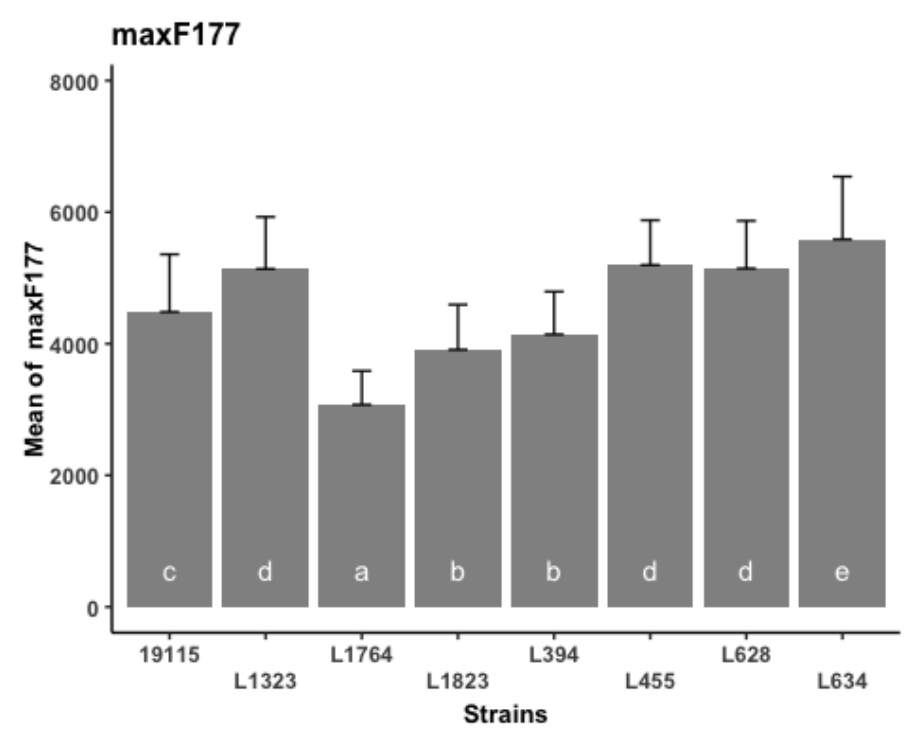

counts (normalized intensity, threshold < 0.21) (F178)

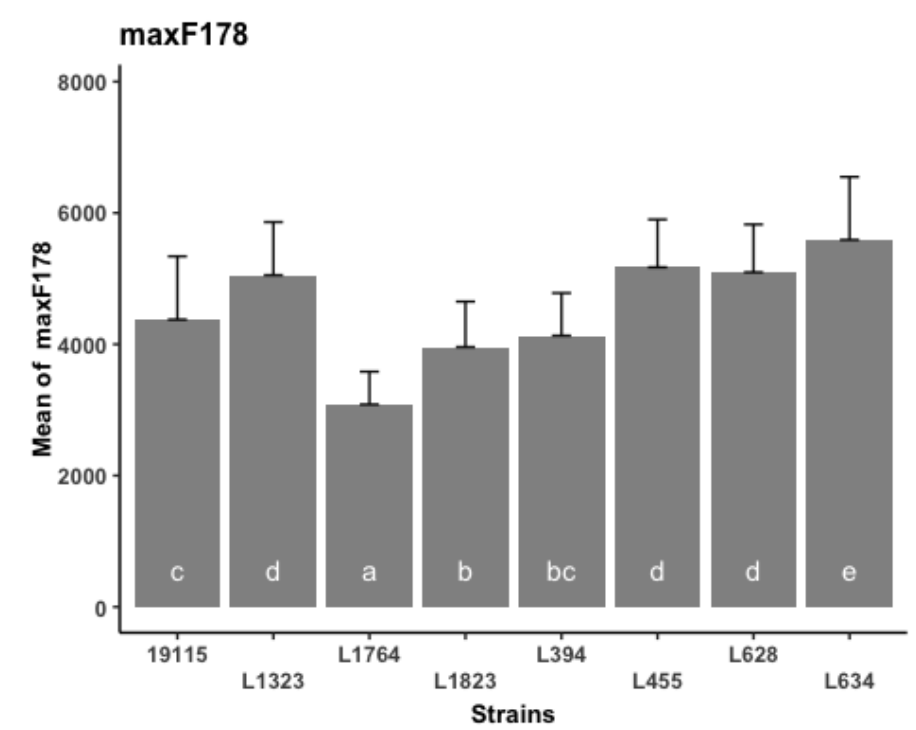

counts (normalized intensity, threshold < 0.22) (F180)

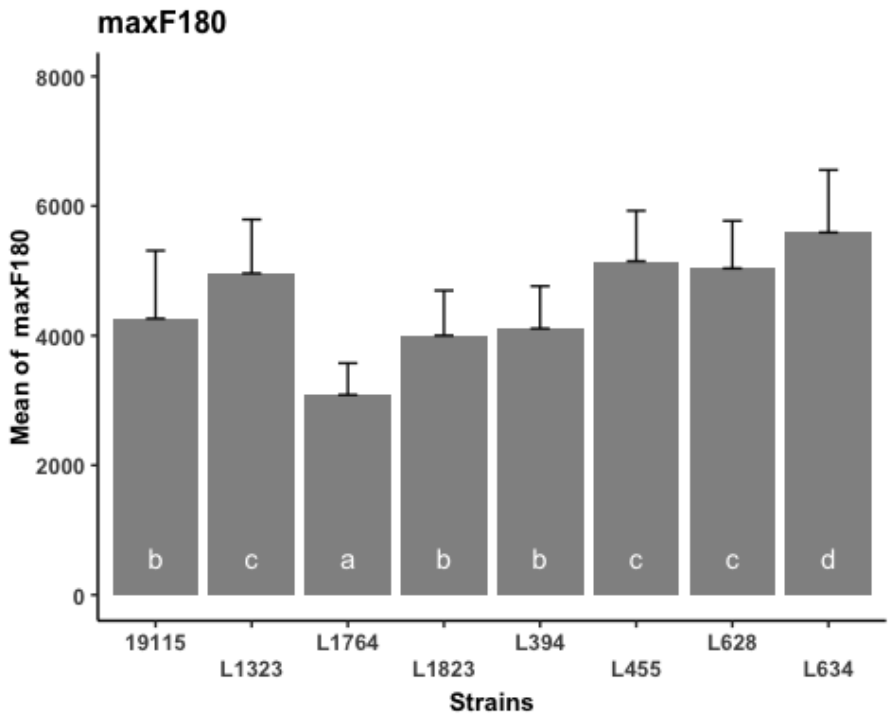

counts (normalized intensity, threshold < 0.23) (F182)

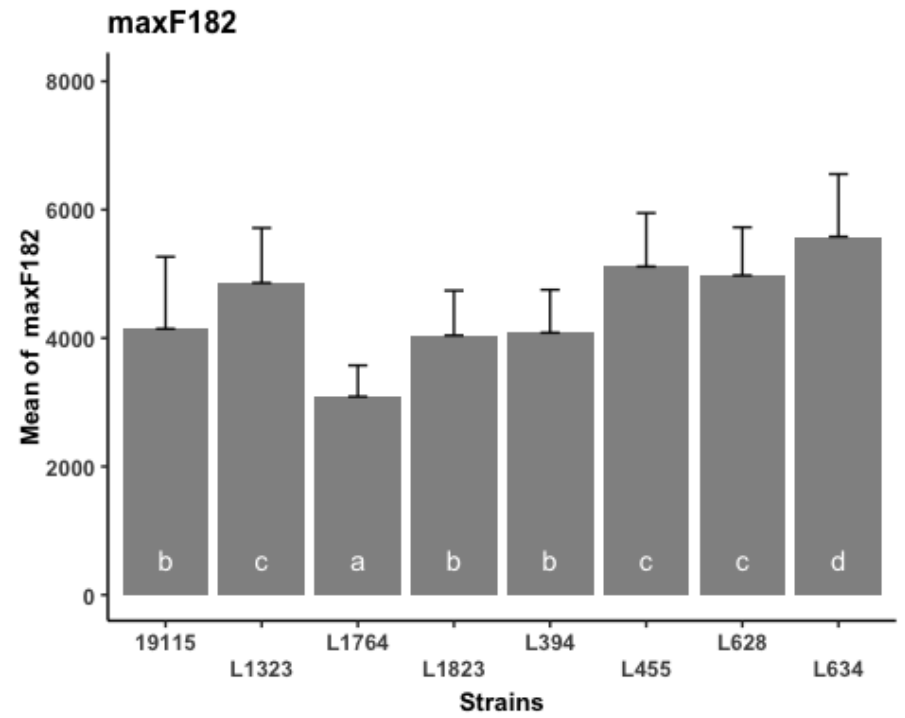

counts (normalized intensity, normalized per layer, threshold < 0.24) (F185)

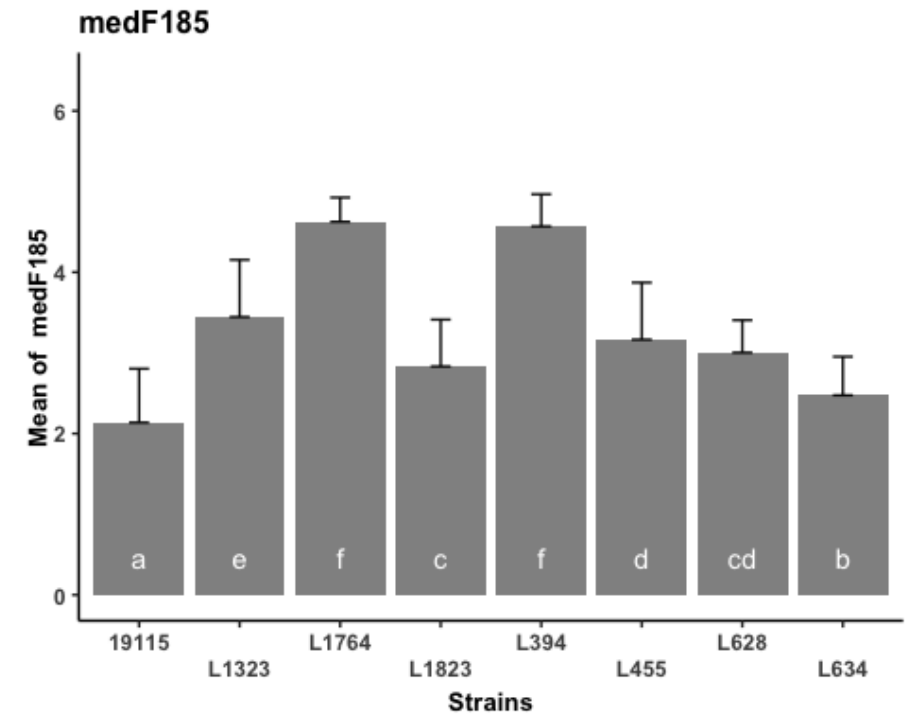

counts (normalized intensity, threshold < 0.26) (F188)

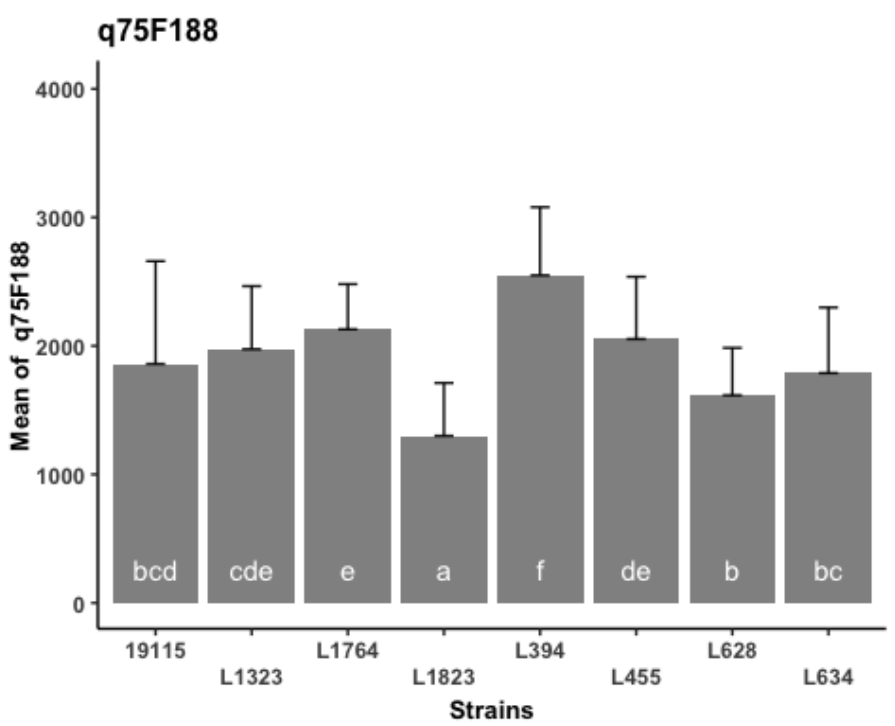

counts (normalized intensity, normalized per layer, threshold < 0.23) (F193)

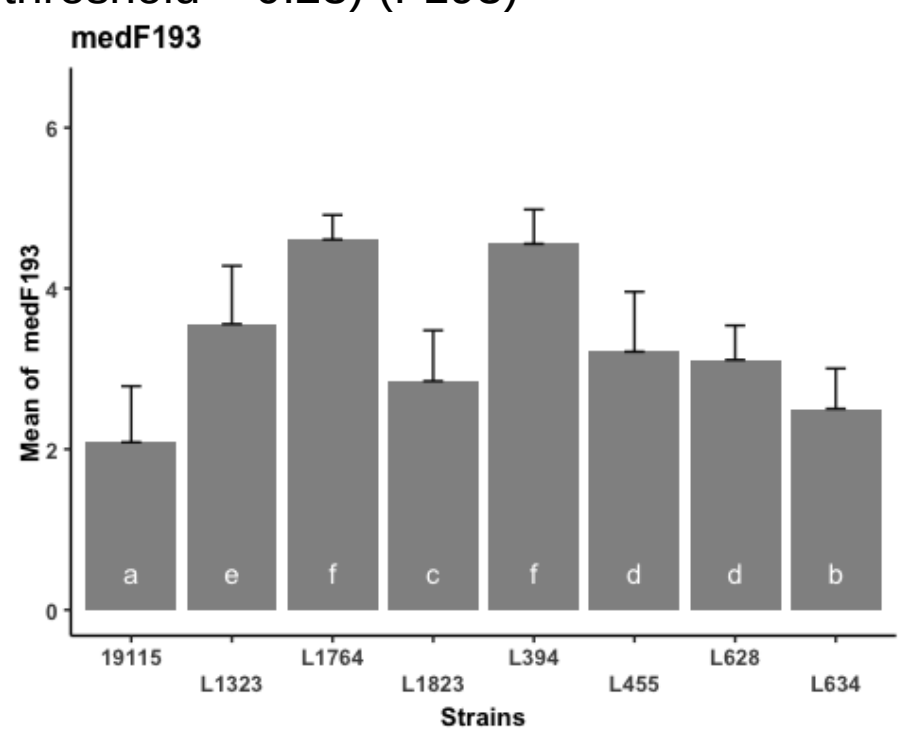

counts (normalized intensity, threshold < 0.29) (F194)

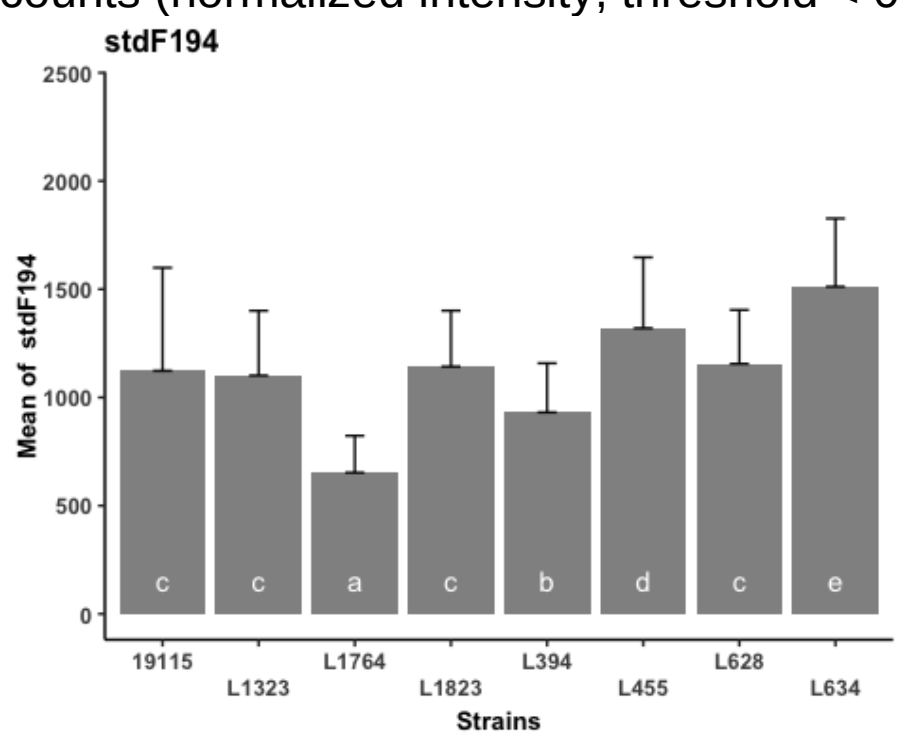

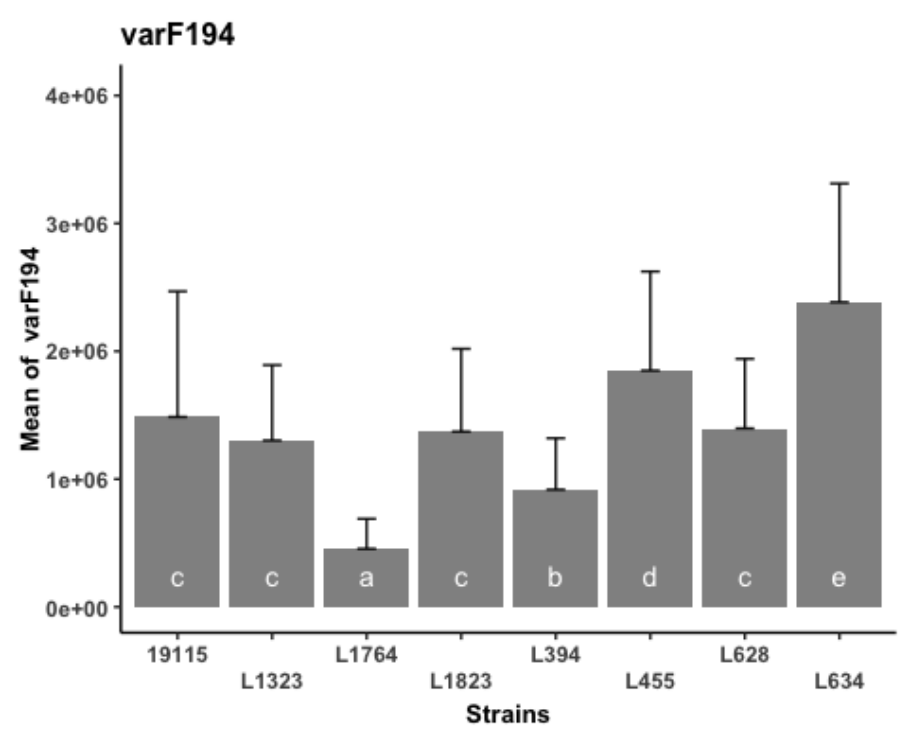

counts (normalized intensity, normalized per layer, threshold < 0.29) (F195)

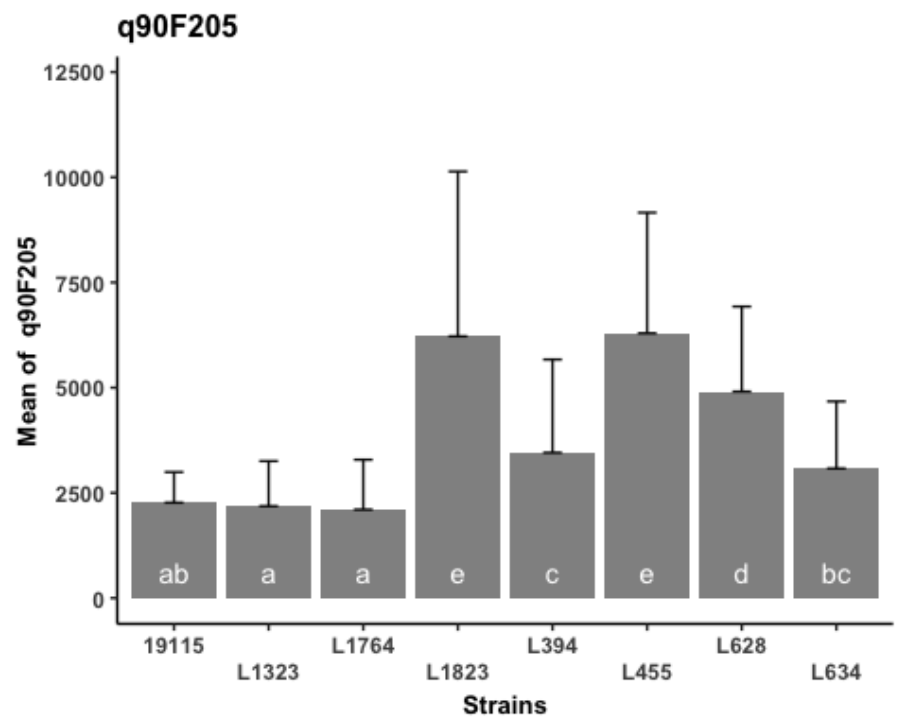

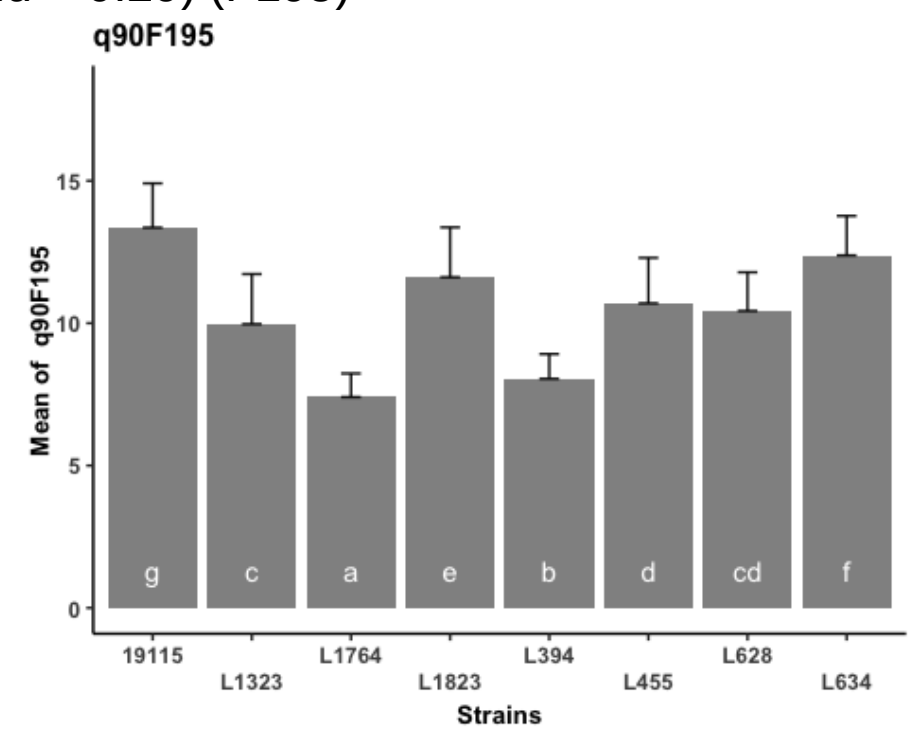

counts (threshold < 0.05) (F205)

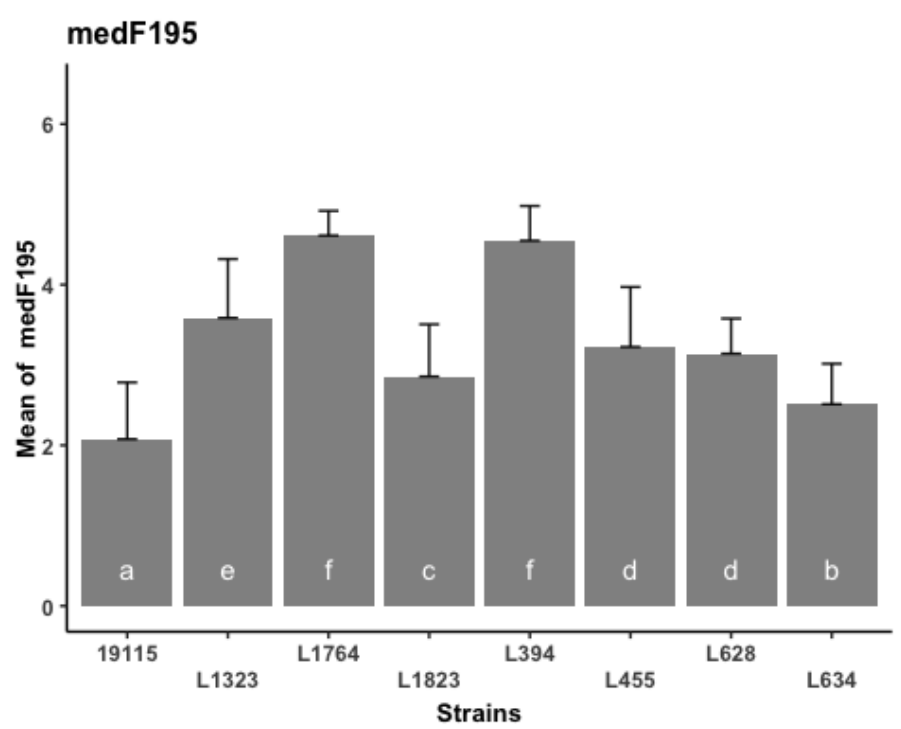

counts (threshold < 0.06) (F207)

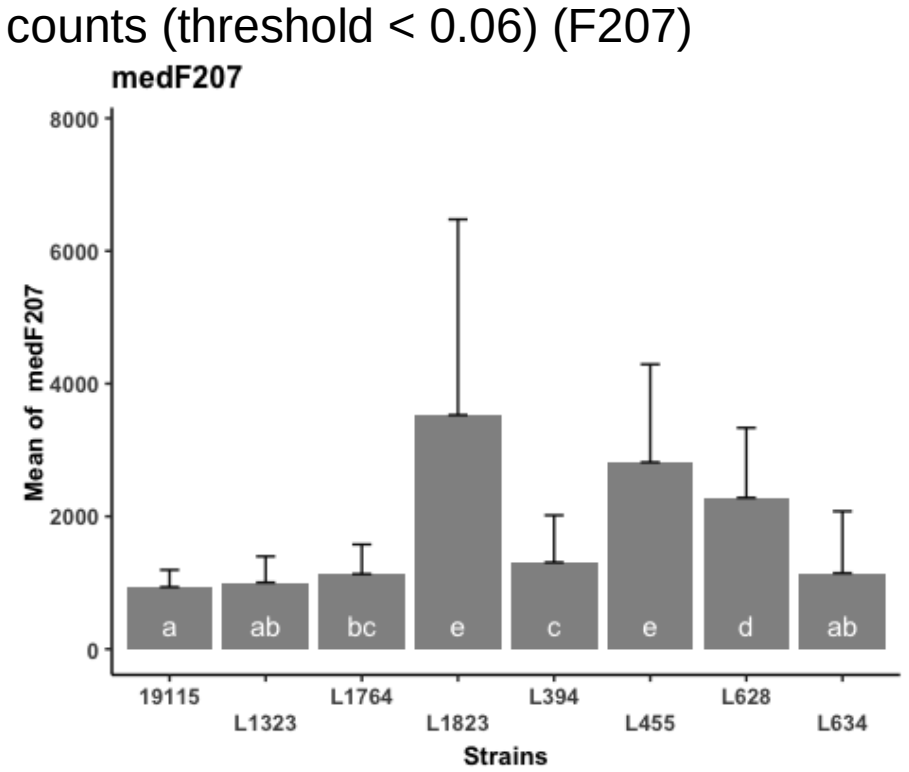

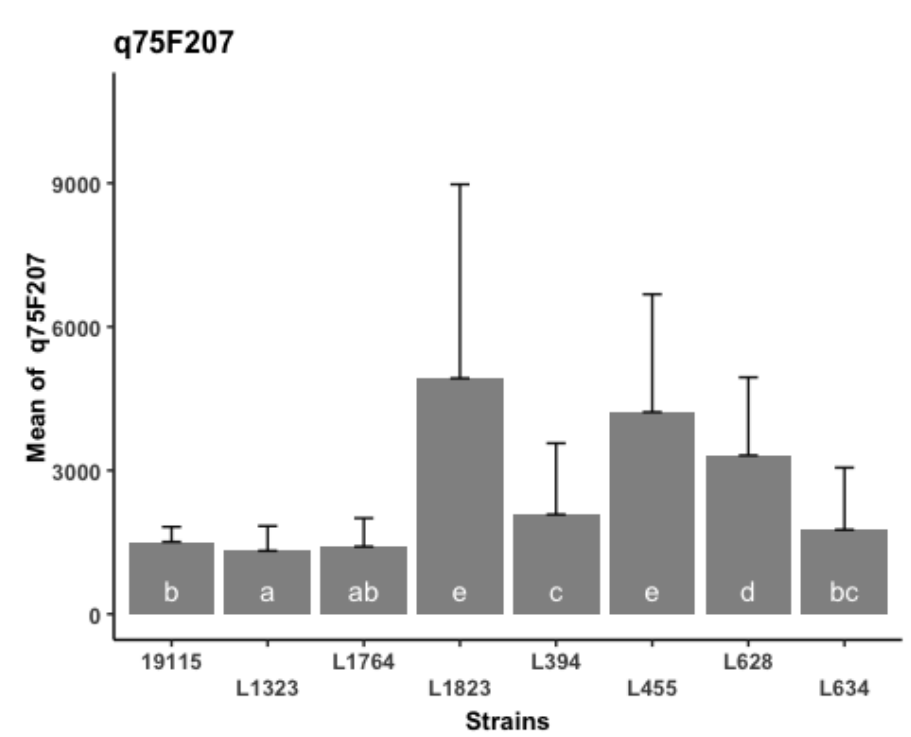

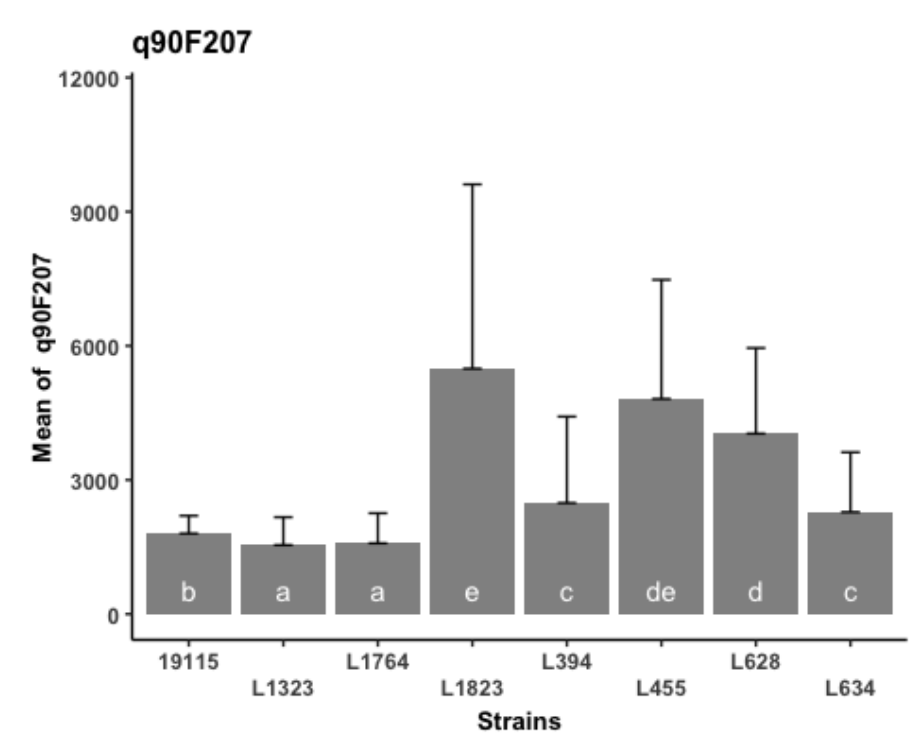

counts (threshold < 0.06) (F207)

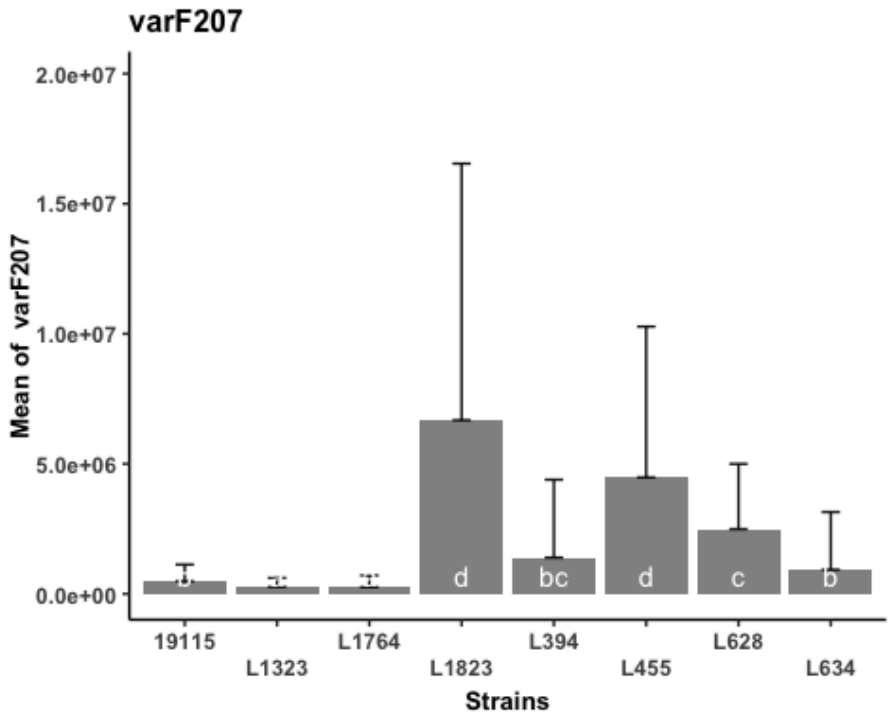

counts (normalized per layer, threshold < 0.06) (q90F208)

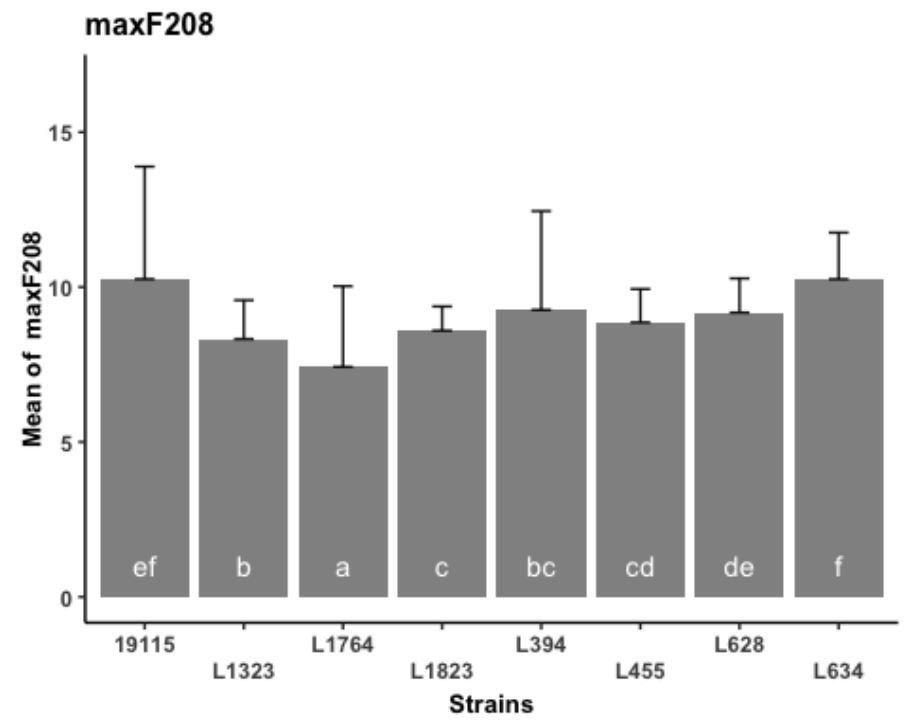

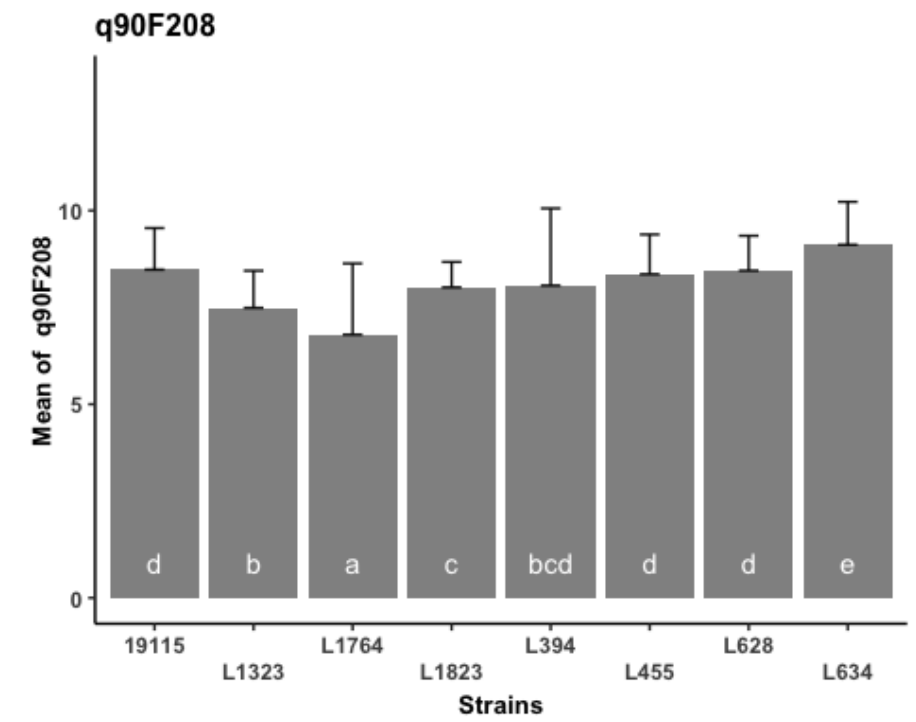

counts (threshold < 0.07) (F209)

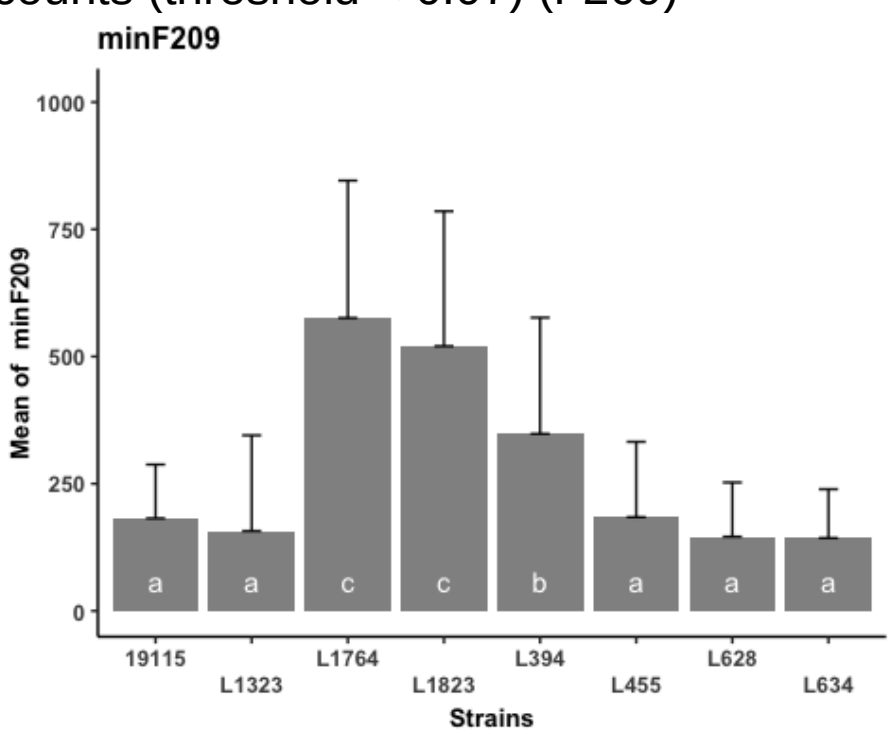

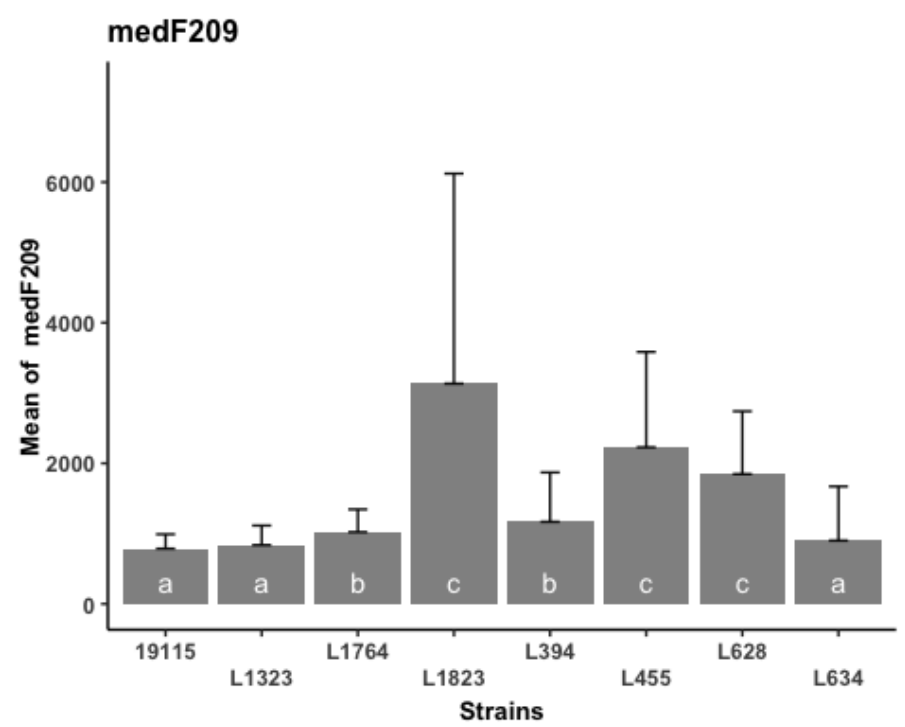

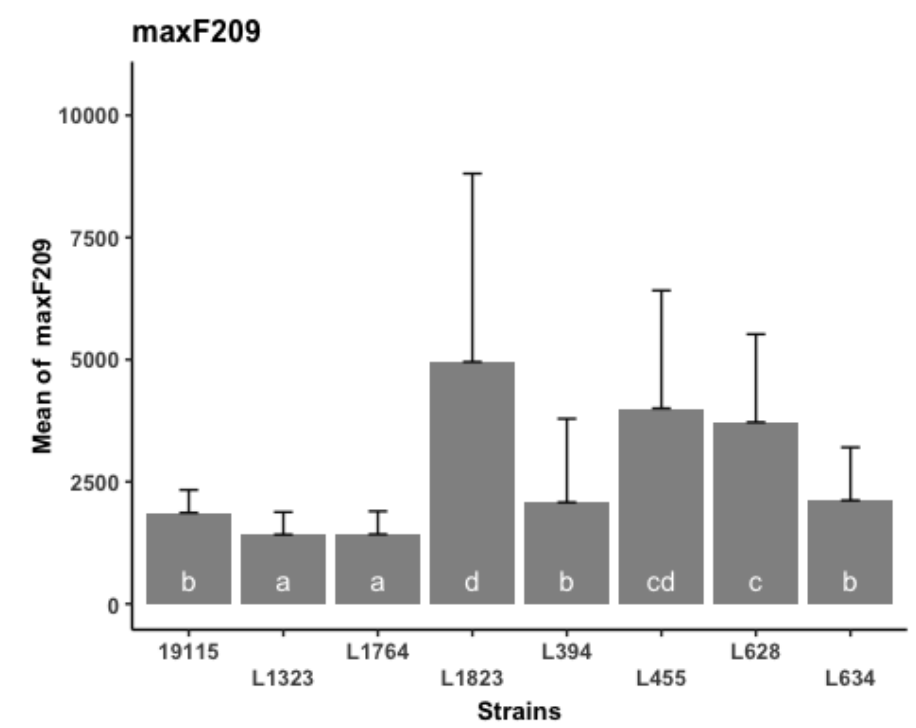

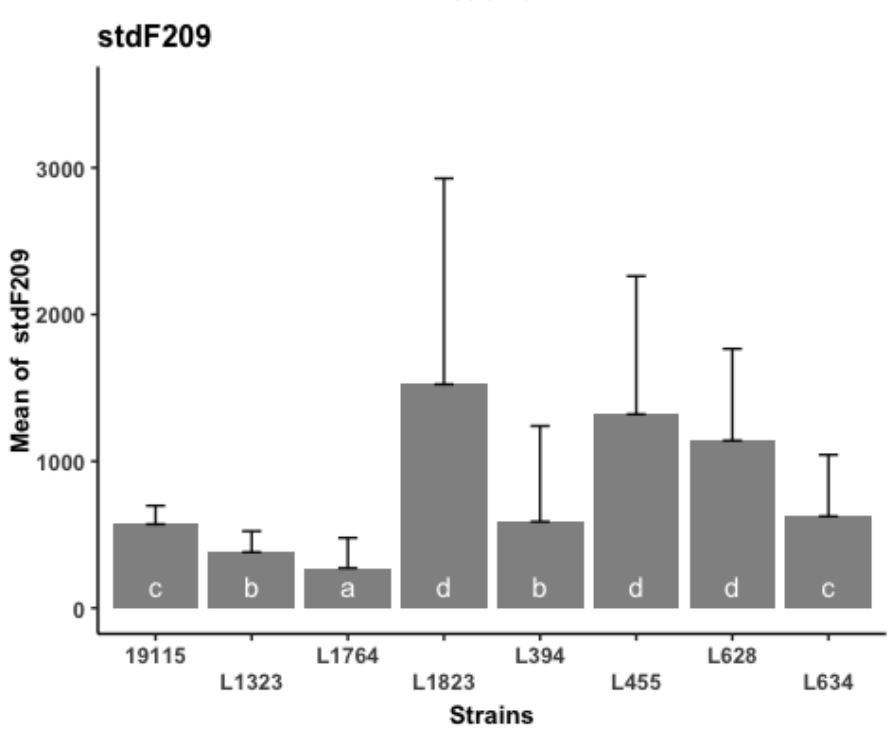

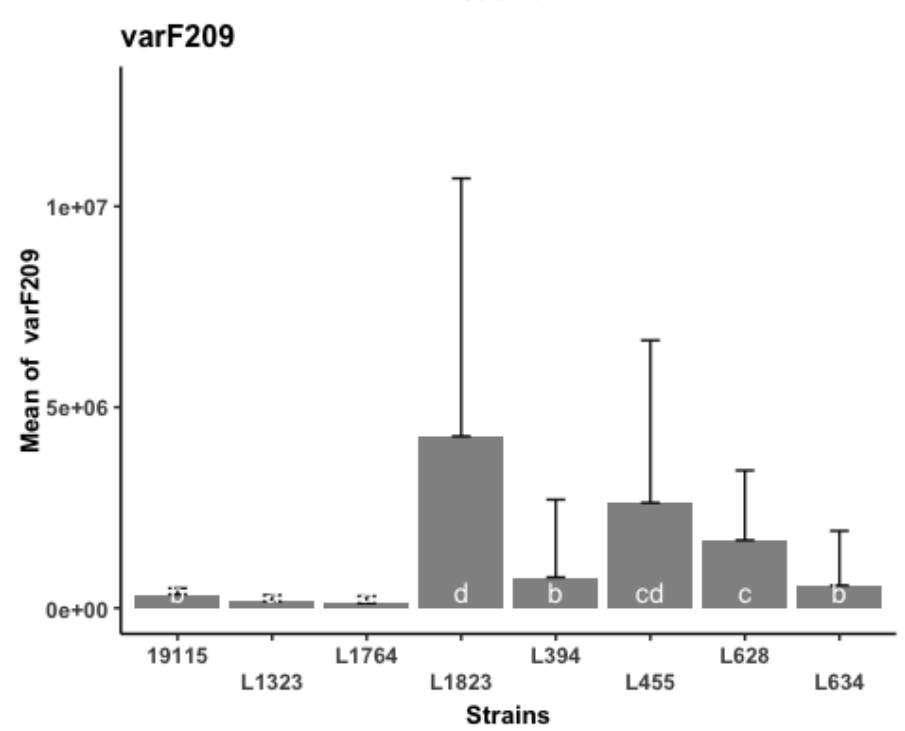

counts (threshold < 0.08) (F211)

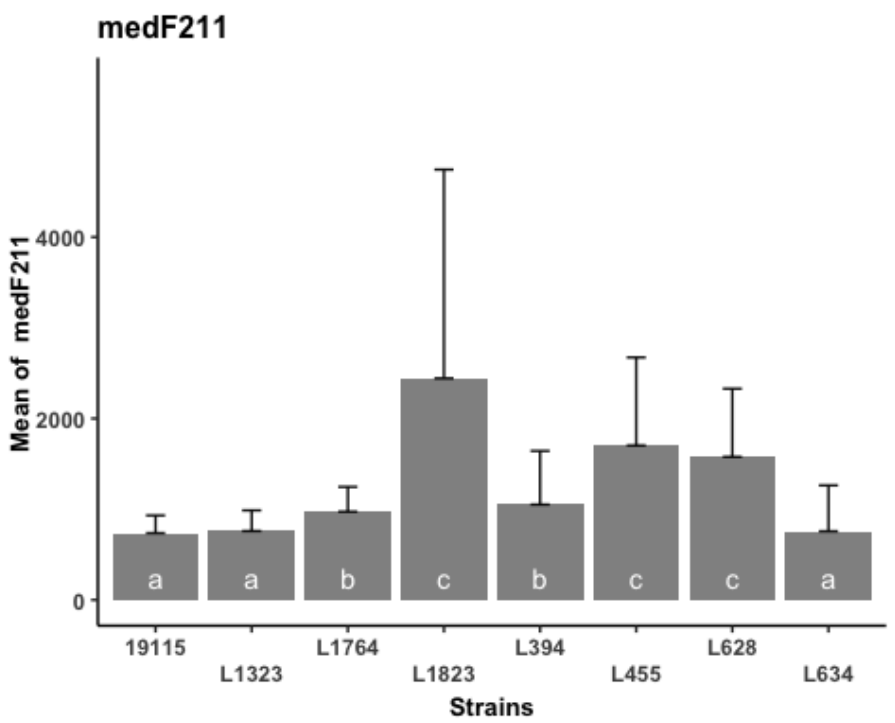

counts (threshold < 0.09) (F213)

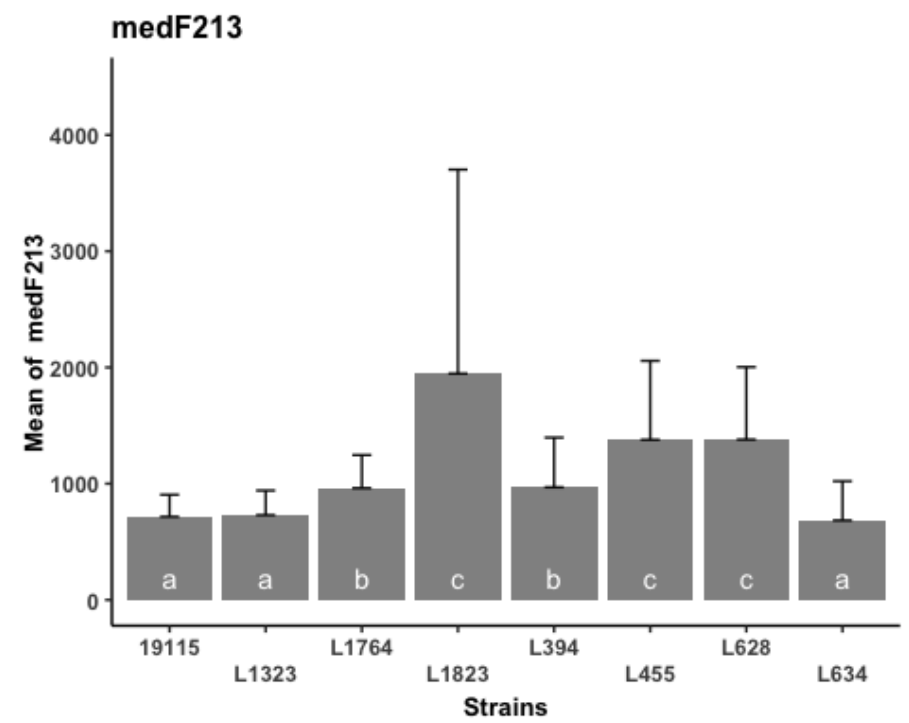

intensity differences (F255)

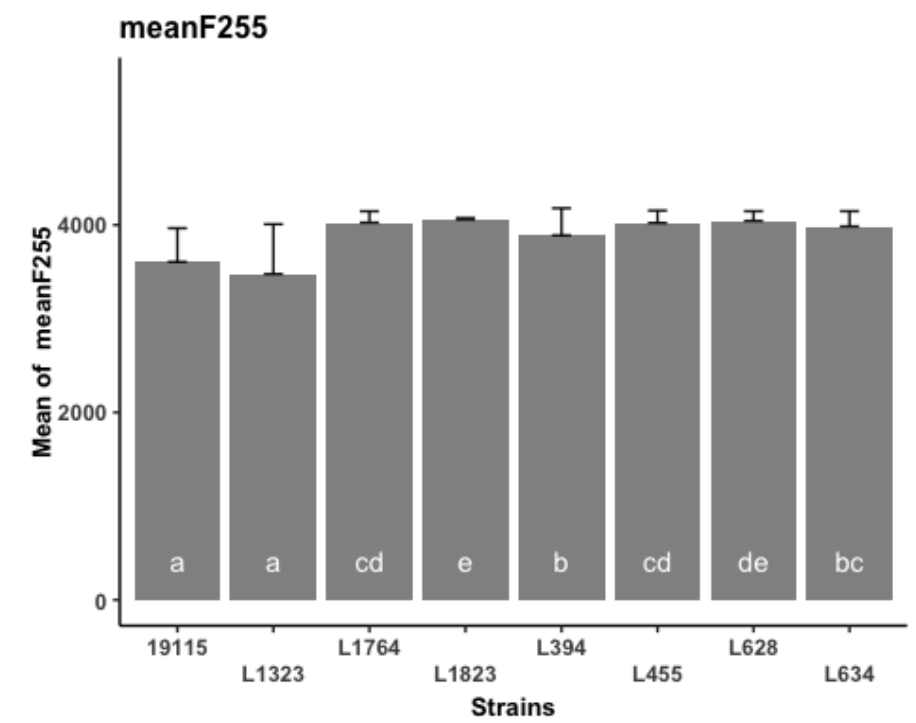

normalized median intensity (F266)

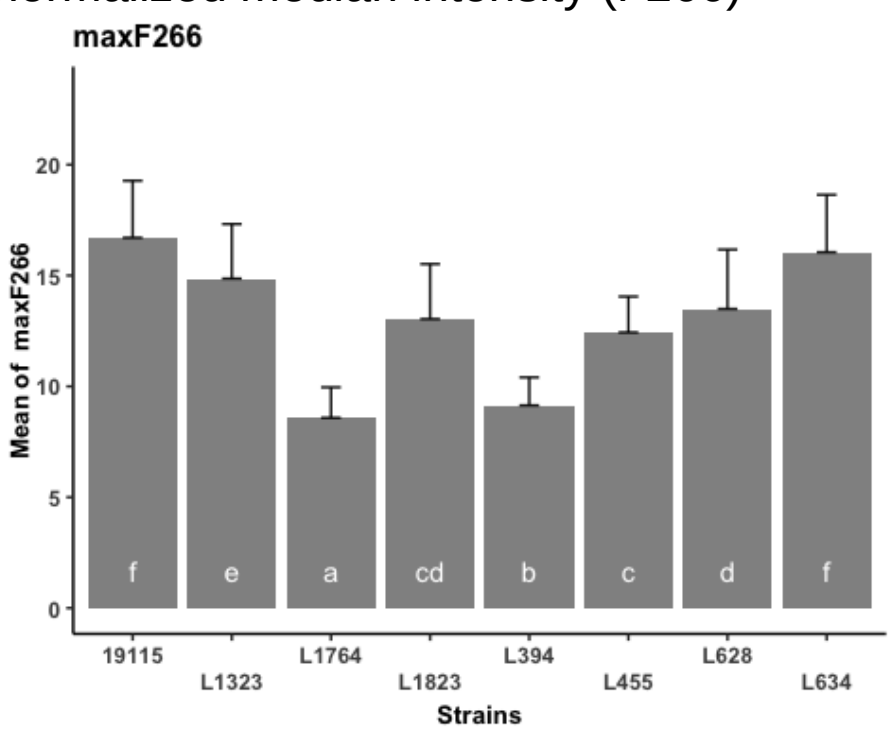

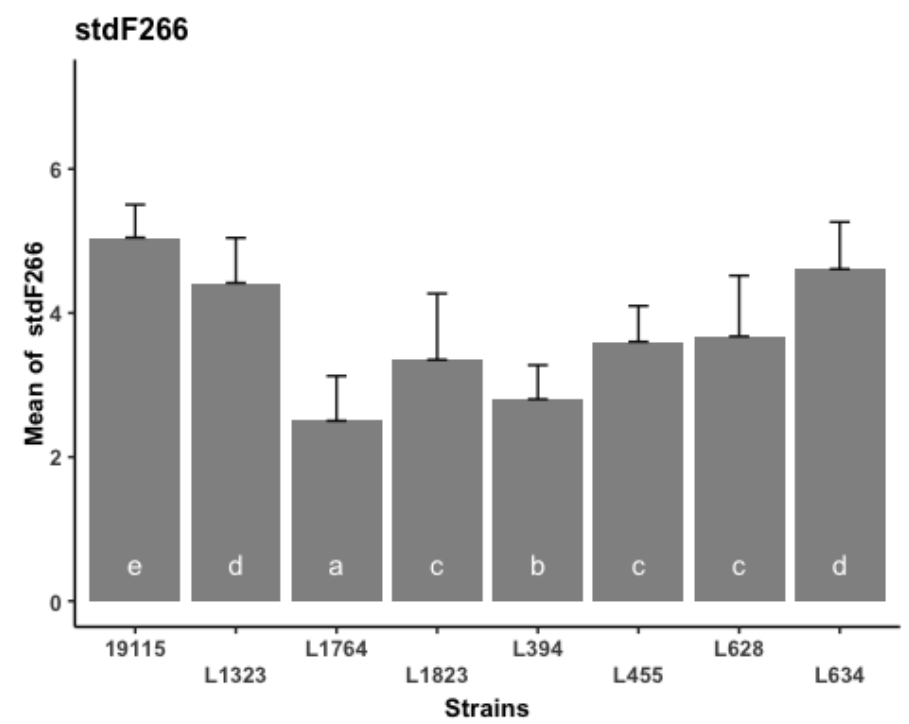

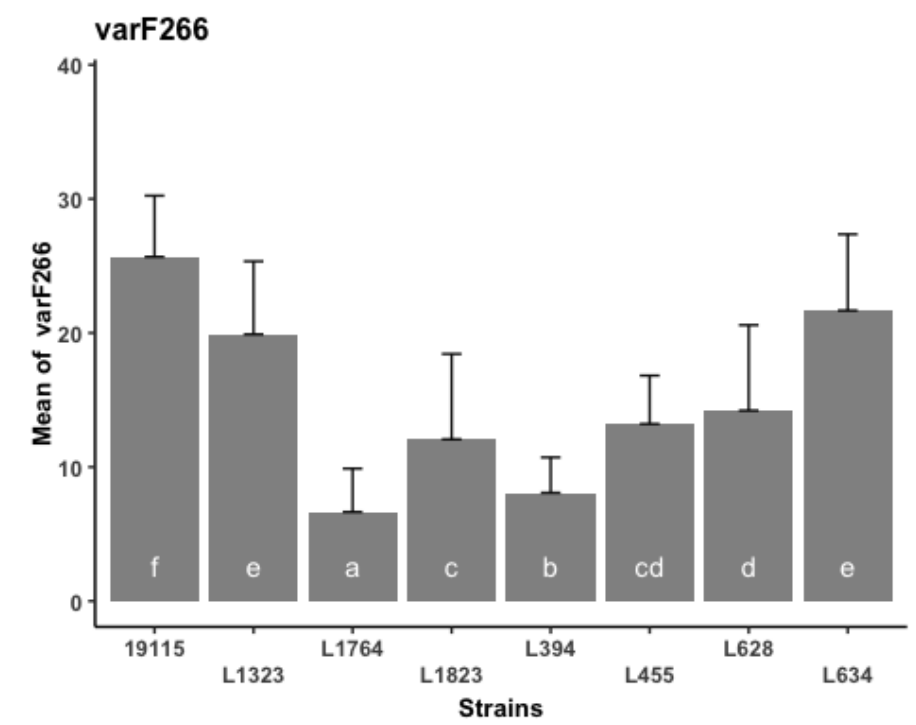

minimum intensity (F267)

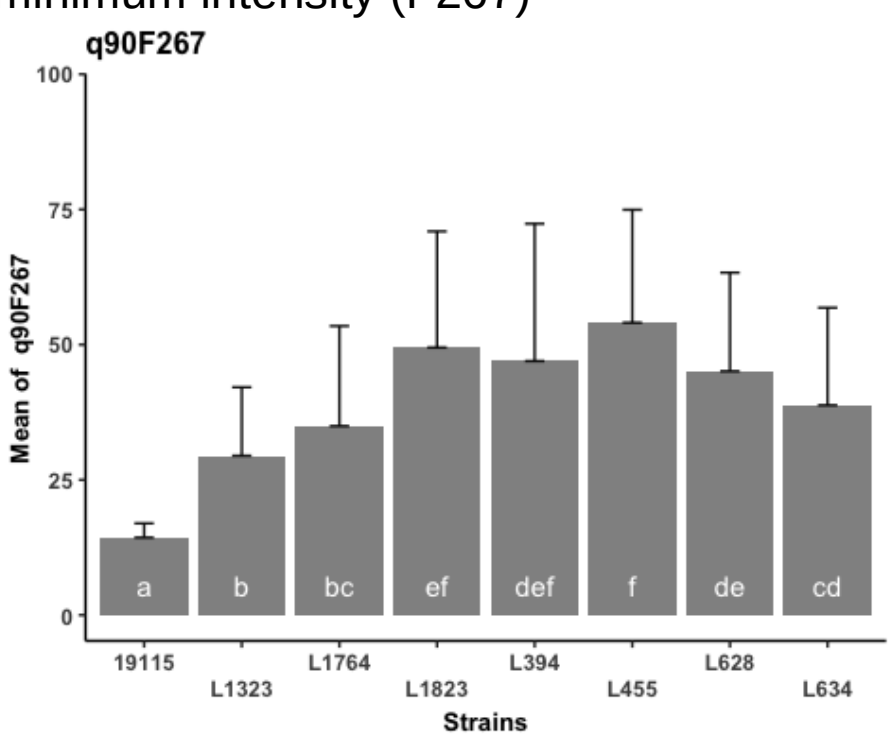

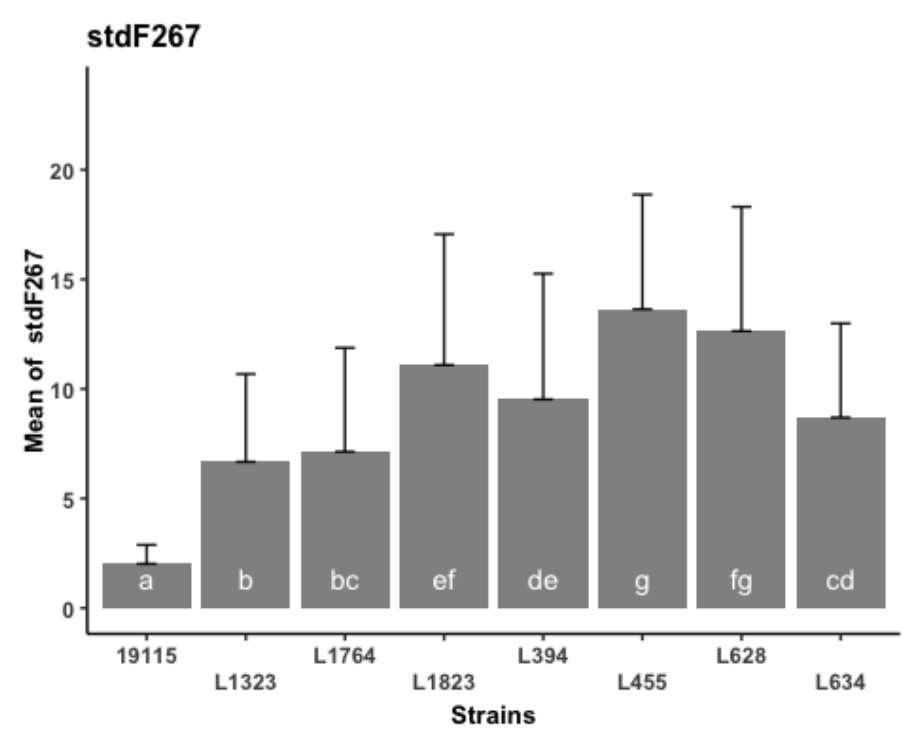

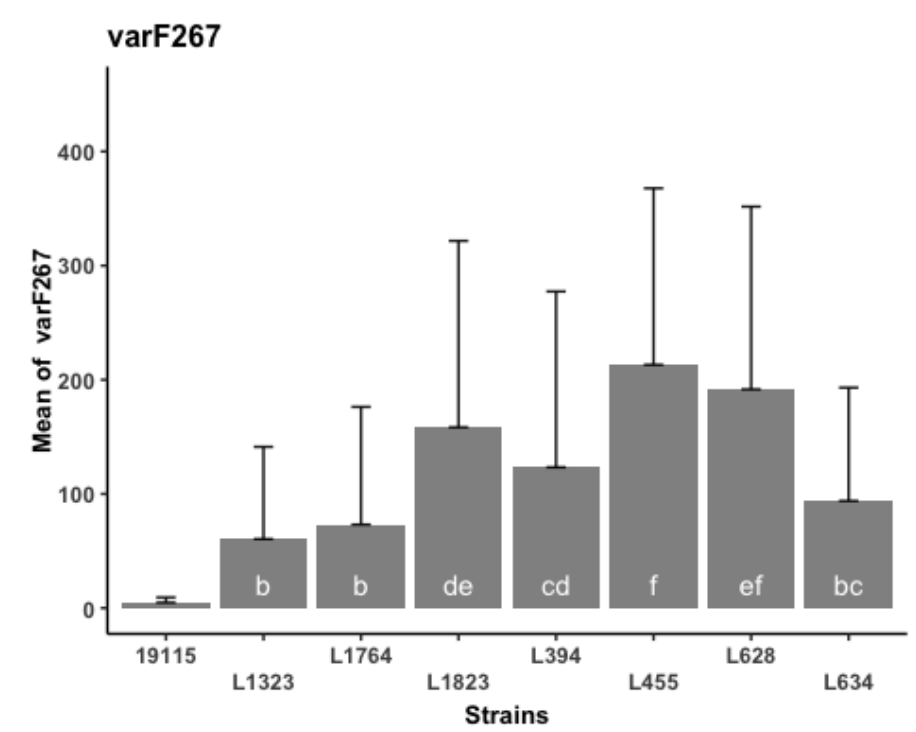

min prop (F269)

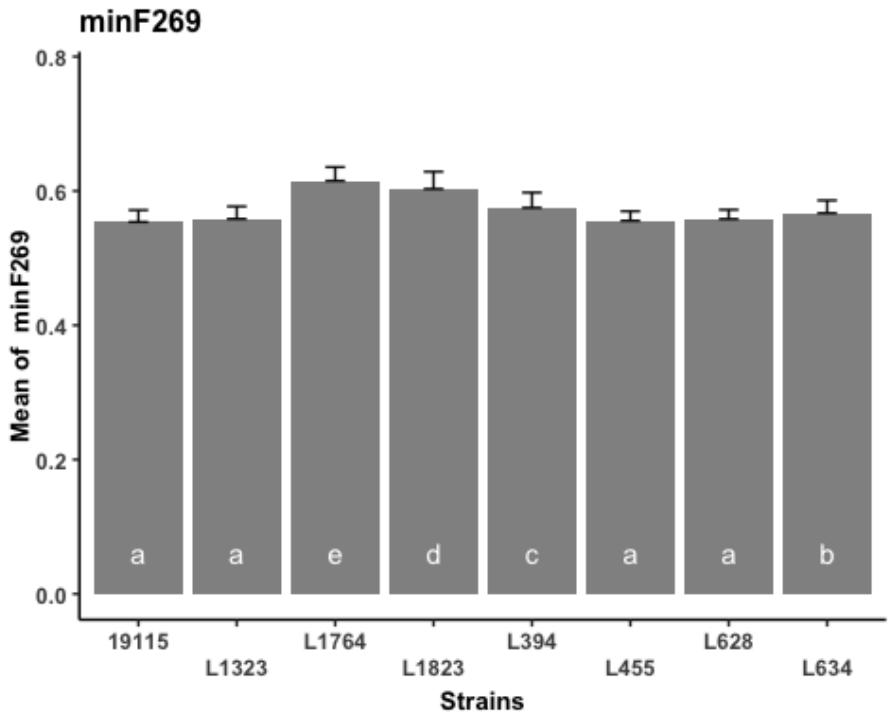

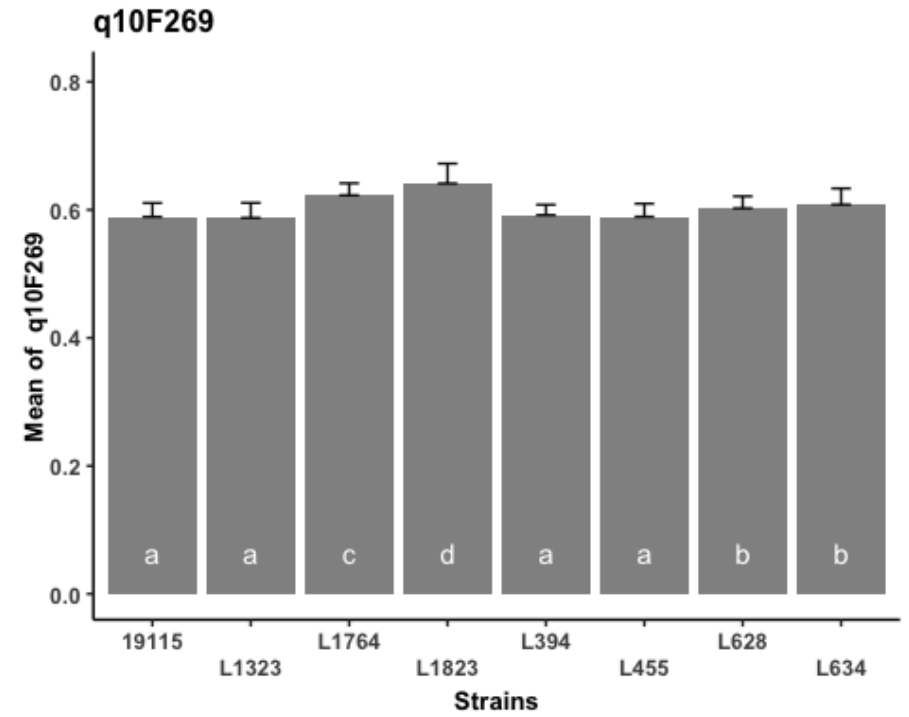

normalized min prop (F270)

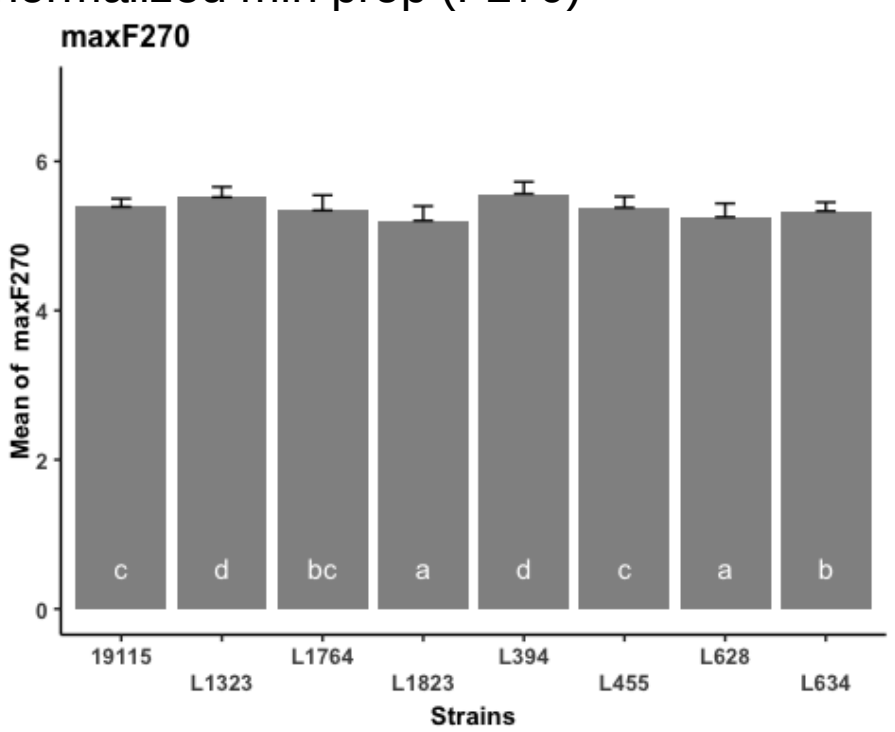

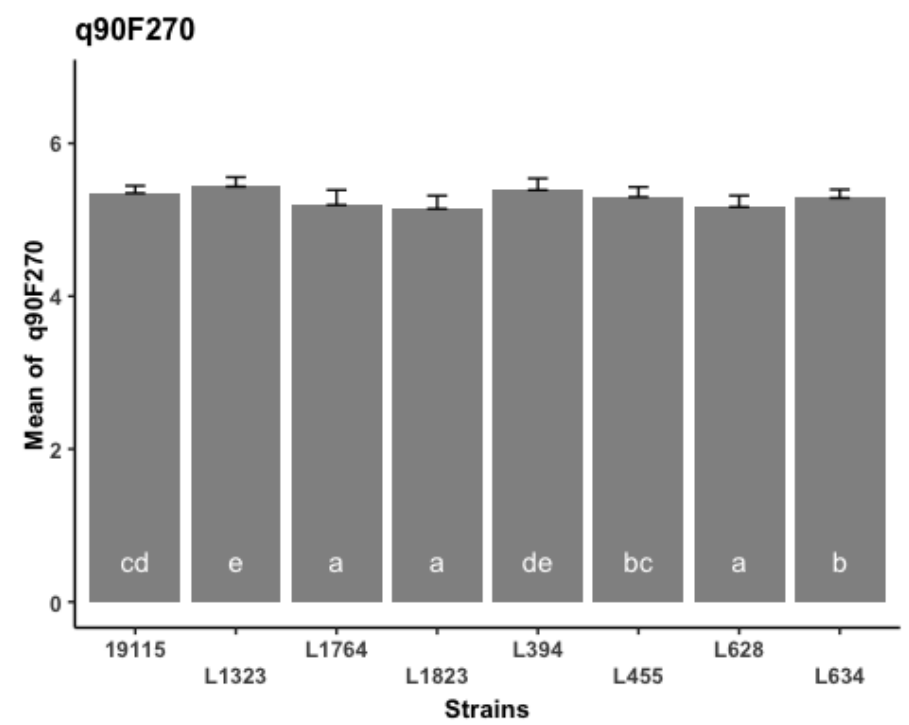

standard deviation of intensity (F272)

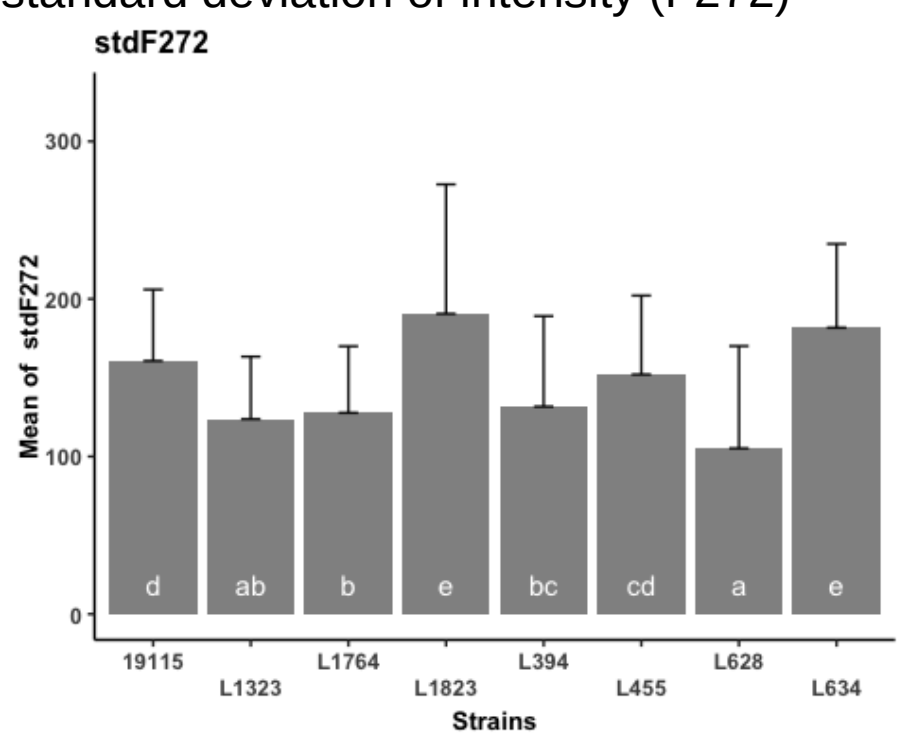

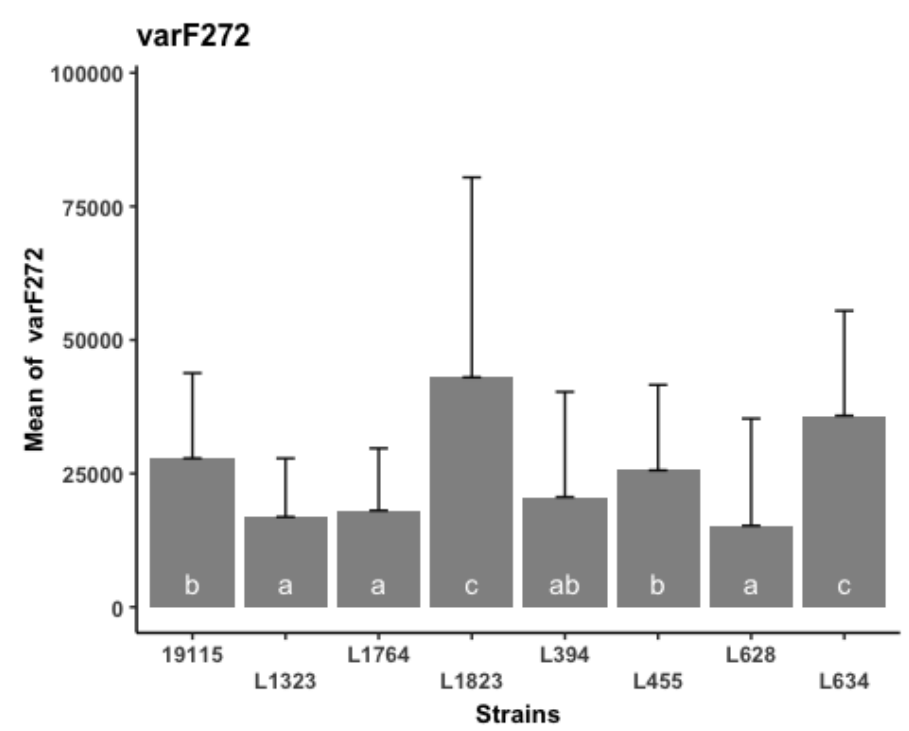

normalized standard deviation of intensity (F273)

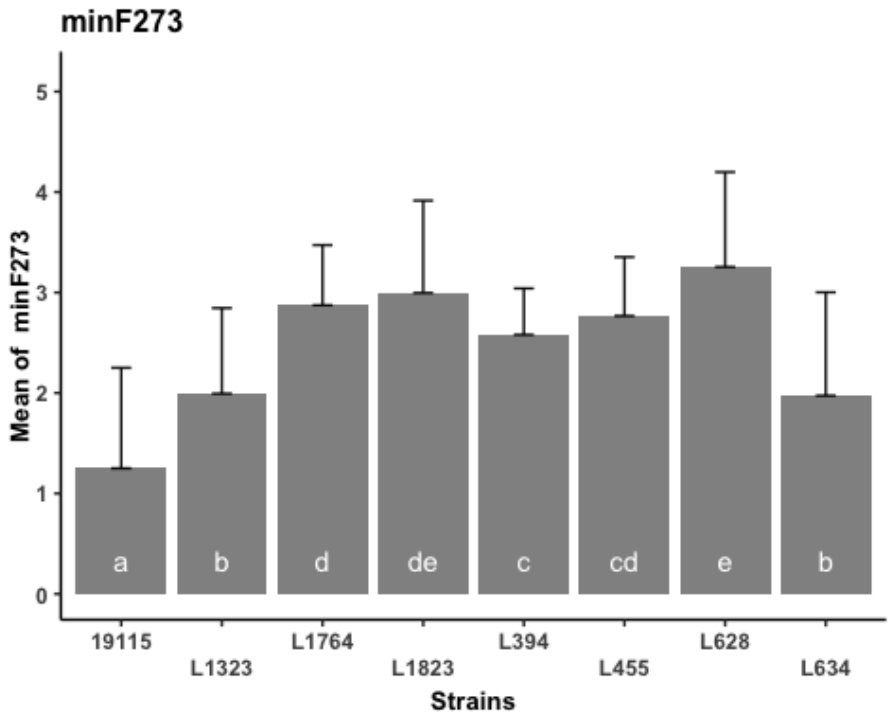

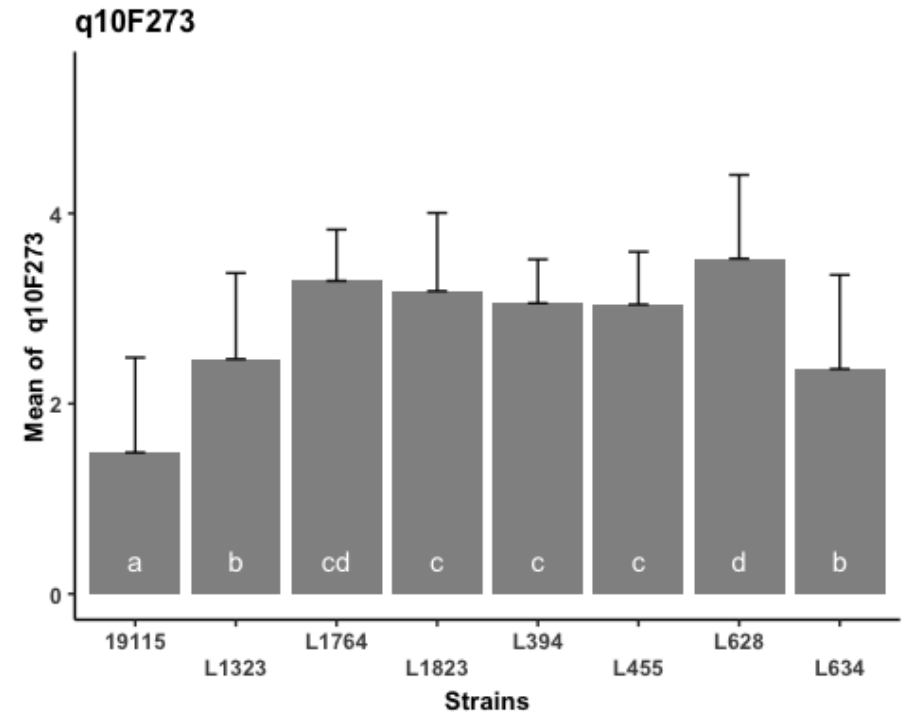

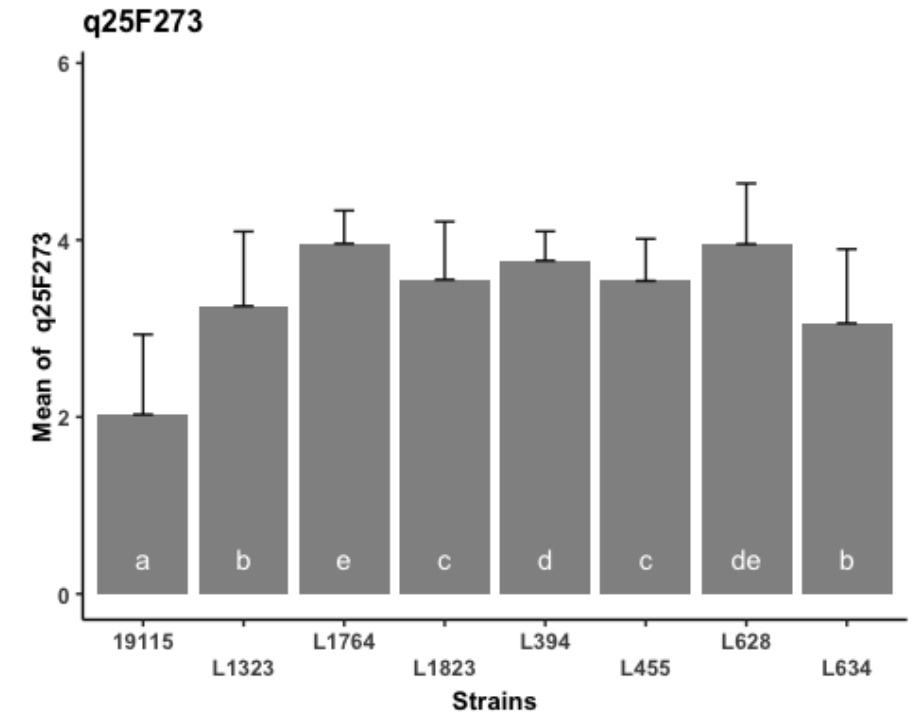

counts (threshold < 0.1) (F214)

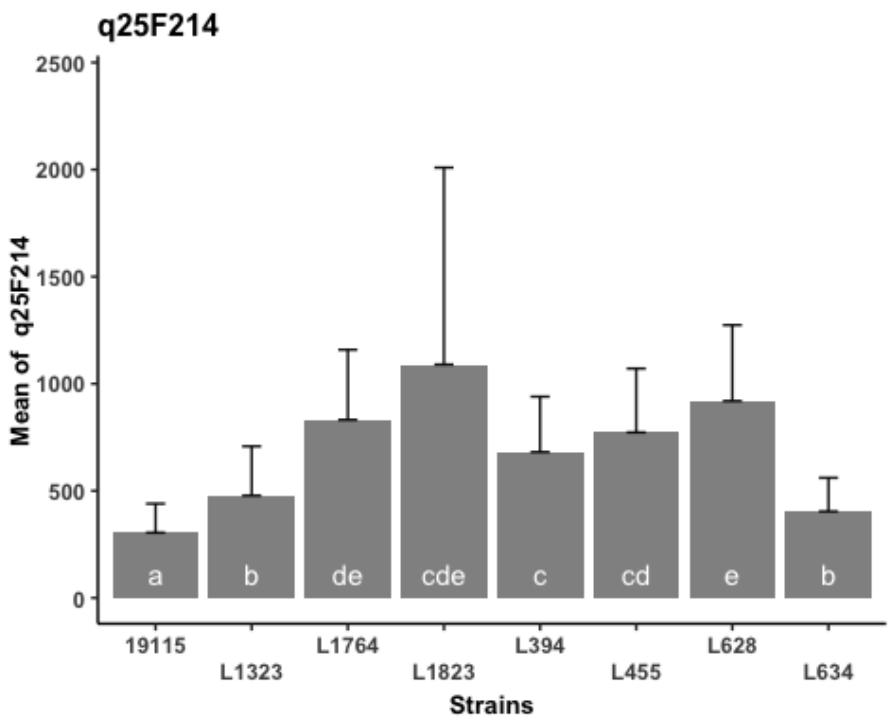

minimum intensity (F267)

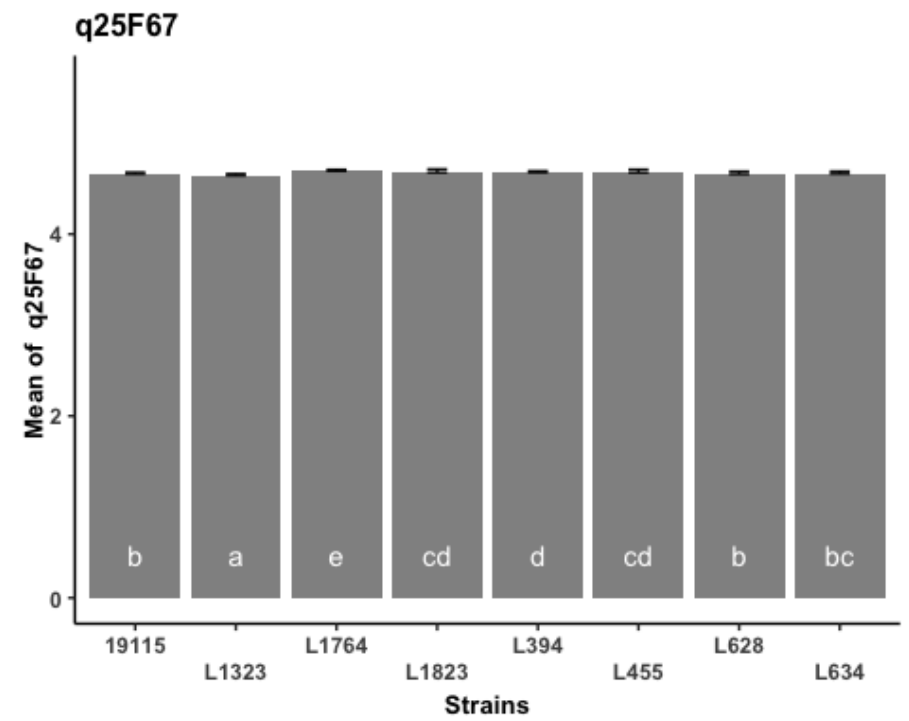

normalized minimum intensity (F268)

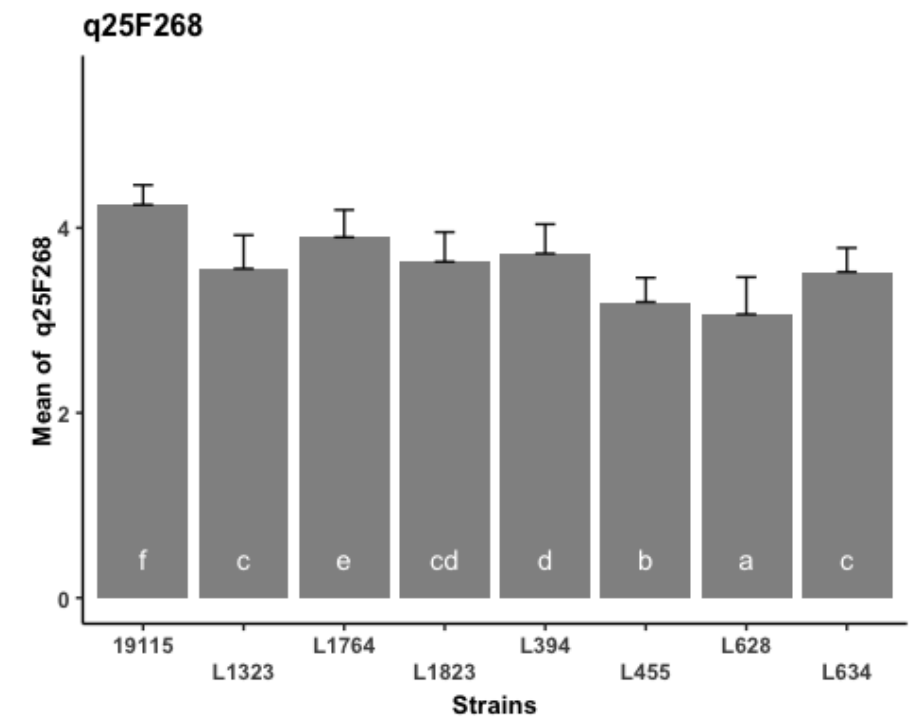

min prop (F269)

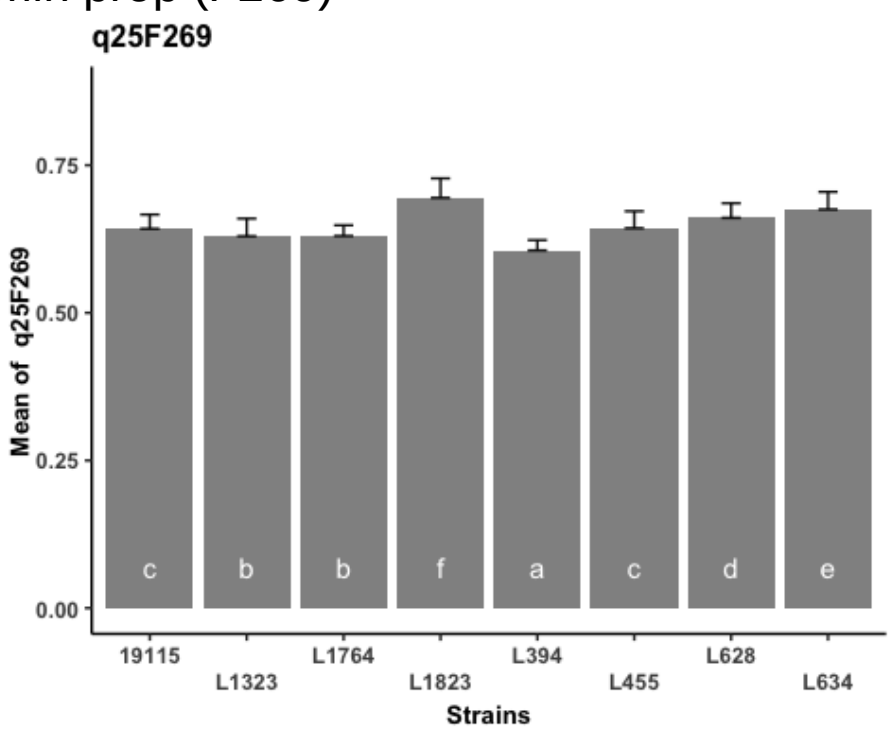

Figure S5. Correlations among the 20 least correlated key features distinguishing between the strains (task identity), represented in a heatmap.

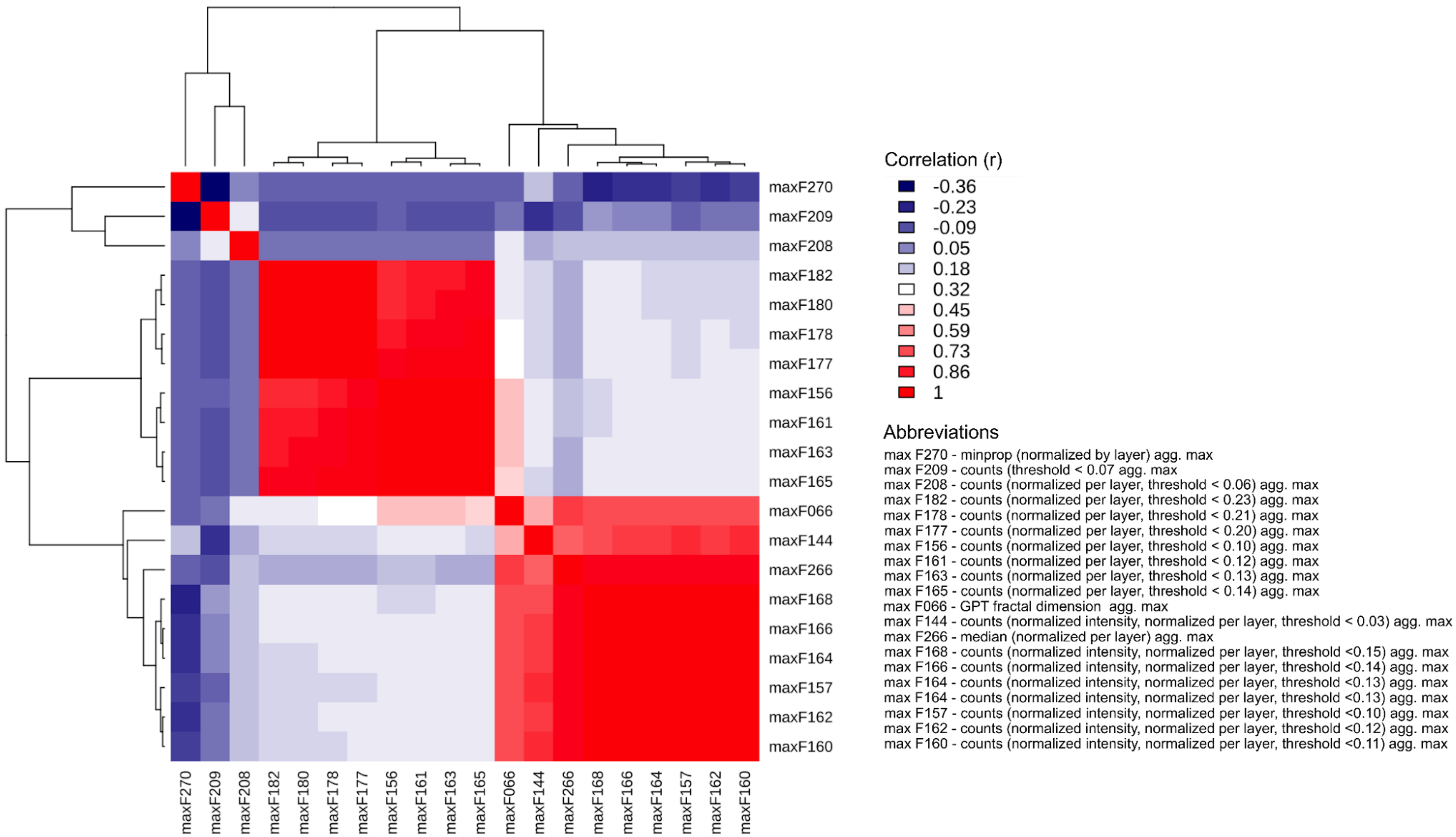

Figure S6. Correlations among the 20 most correlated key features distinguishing between the strains (task identity), represented in a heatmap.

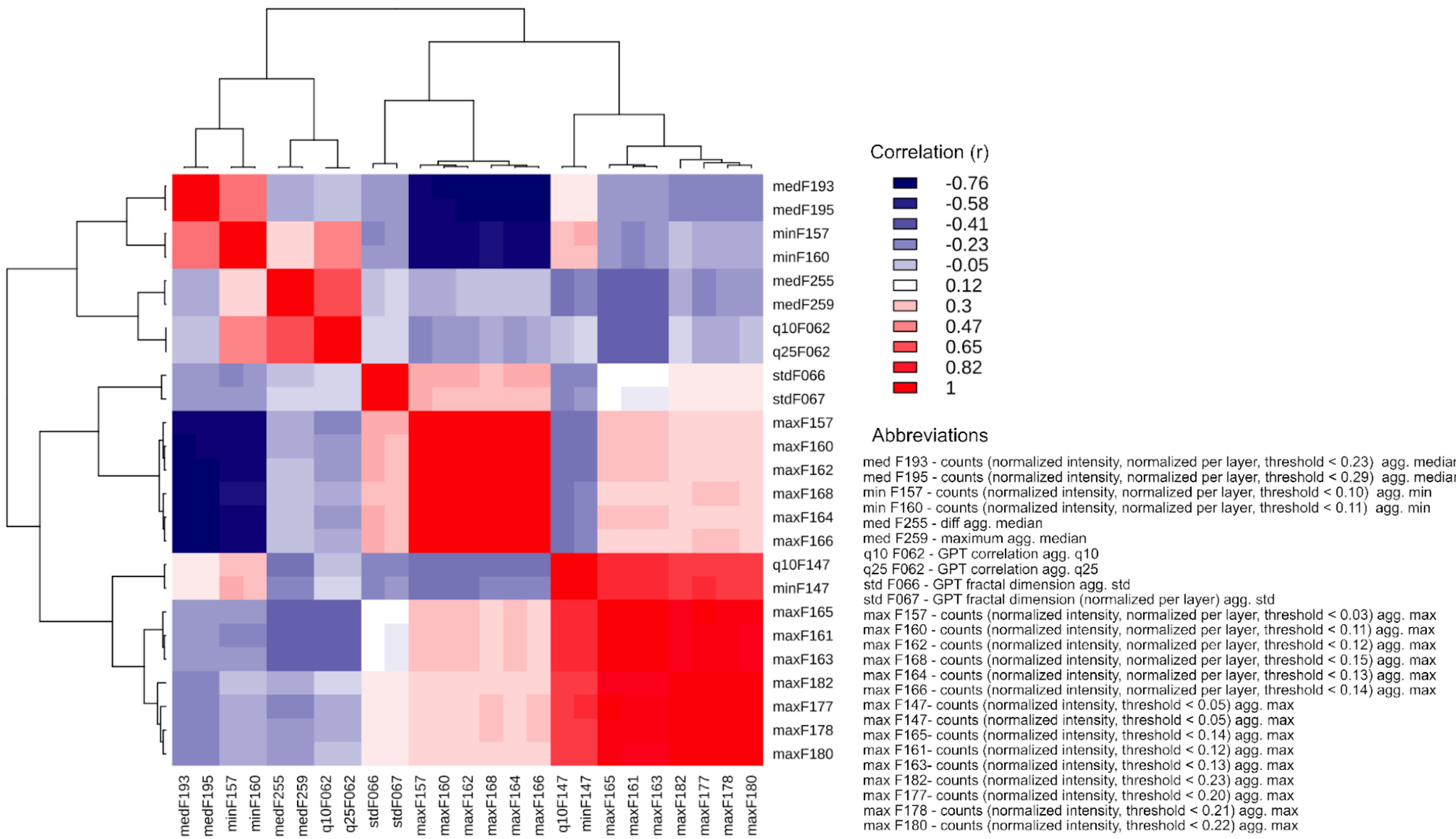

Figure S7. Classifier performance for *L. monocytogenes* strain identity prediction using the combined MicroICS and BiofilmQ feature set. (a) Classification accuracy across all computed features (full feature set: 3,040 dimensions; projected feature set: up to 512 dimensions), with and without the 'counts' feature, allowing assessment of its contribution to model performance. (b) Feature ablation analysis showing classification accuracy as a function of the number of top-ranked features included, identifying the minimal feature set required for optimal performance.

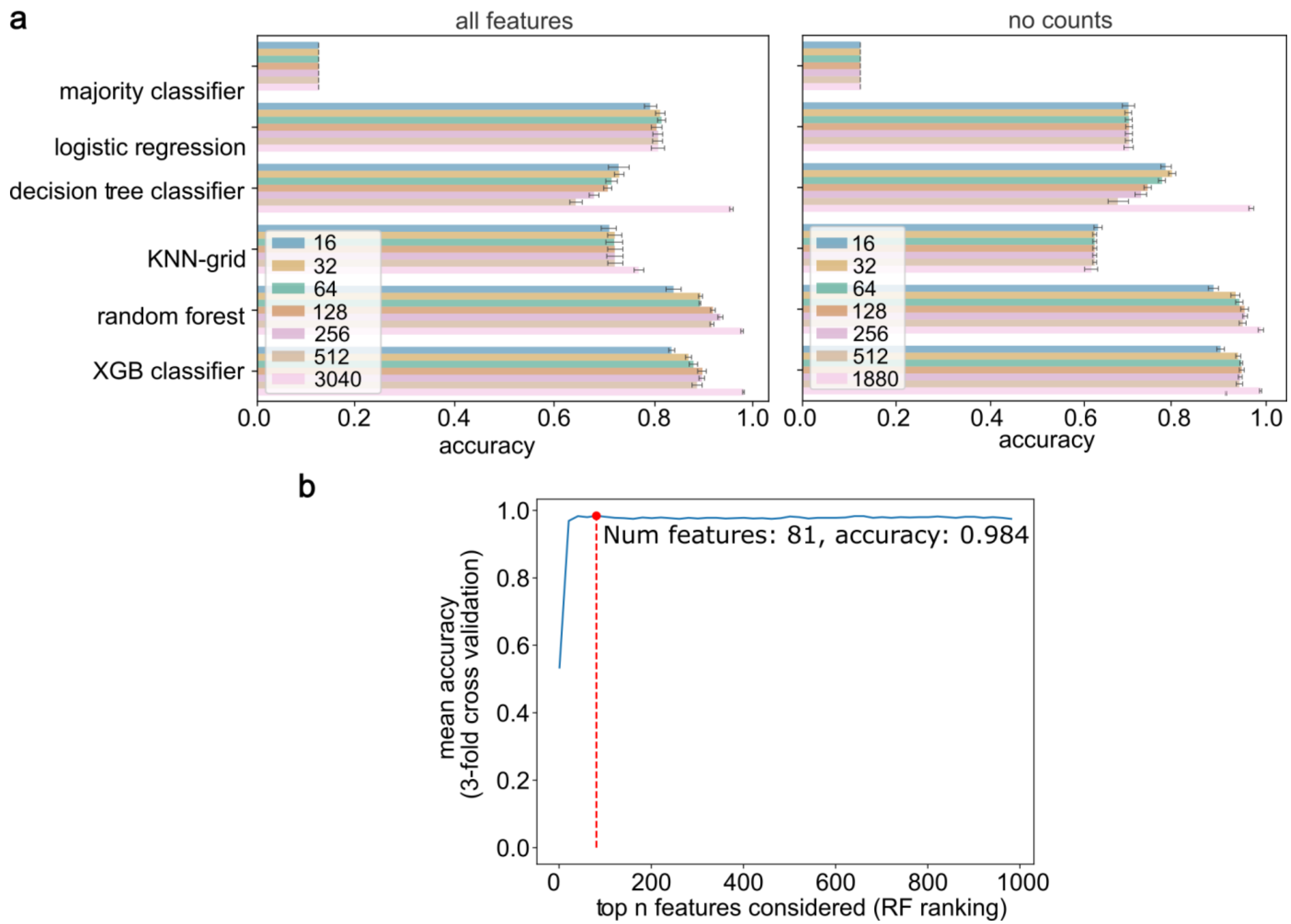

Table S5. The 81 most influential features for *L. monocytogenes* strain classification were selected using random forest-based importance ranking and ablation analysis, based on the combined feature sets calculated by MicroICS (this work) and BiofilmQ. BiofilmQ features were derived using a declumping value of 10 unless otherwise stated; where non-declumped segmentation was used, this is indicated in brackets (no declumping). Features that overlap with those predictive of experimental date (i.e. acquisition batch) may reflect temporal or procedural variation rather than intrinsic strain differences, and should therefore be interpreted with caution as potential confounders rather than reliable biological descriptors.

score	feature name	aggregation type	found also in ranking according to the date among 81 features	tool
0.097832	Cube_Surface	std_biovolume	yes	BiofilmQ
0.077868	Surface_LocalRoughness_range10	std	yes	BiofilmQ
0.077783	Cube_Surface	std	yes	BiofilmQ
0.052158	counts (normalized intensity, threshold < 0.05	min	no	MicroICS
0.049172	minimum	std	no	MicroICS
0.045386	minimum	var	no	MicroICS
0.045275	Shape_Volume	std	yes	BiofilmQ
0.040572	Shape_Volume	mean_biovolume	yes	BiofilmQ
0.039399	VolumeFraction	mean_biovolume	yes	BiofilmQ
0.034994	Cube_VolumeFraction	std	yes	BiofilmQ
0.025644	Cube_Surface	mean_biovolume	no	BiofilmQ
0.018952	normalized counts (normalized intensity, threshold < 0.19)	max	no	MicroICS
0.018818	Surface_LocalRoughness	mean_biovolume	no	BiofilmQ
0.014932	normalized counts (normalized intensity, threshold < 0.18)	max	no	BiofilmQ
0.014365	Biofilm_OuterSurfaceIgnoreSubstratePerVolume	/	yes	BiofilmQ

score	feature name	aggregation type	found also in ranking according to the date among 81 features	tool
0.01297	counts (normalized intensity, threshold < 0.13	max	no	MicroICS
0.012265	Biofilm_OuterSurfacePerVolume	/	no	BiofilmQ
0.011231	normalized minimum	q10	no	MicroICS
0.010009	Surface_LocalRoughness_range10	p25	no	BiofilmQ
0.009794	Surface_LocalRoughness_range10	std	yes	BiofilmQ
0.009676	normalized minimum	q25	no	MicroICS
0.008819	Shape_Volume (none declumping)	p75	no	BiofilmQ
0.008763	Shape_Volume (none declumping)	median	no	BiofilmQ
0.008491	Surface_LocalRoughness_range10	p75	no	BiofilmQ
0.007848	normalized counts (normalized intensity, threshold < 0.14)	max	no	MicroICS
0.007762	normalized counts (normalized intensity, threshold < 0.05)	q10	no	MicroICS
0.0077	Intensity_Mean_ch1_noBackground (no declumping)	min	no	BiofilmQ
0.007469	Texture_Haralick_MaxProbability_ch1_range5	mean_biovolume	no	BiofilmQ
0.006267	counts (normalized intensity, threshold < 0.06)	q10	no	MicroICS
0.006239	counts (normalized intensity, threshold < 0.07)	q10	no	MicroICS
0.005772	Surface_LocalRoughness_range10	mean	no	BiofilmQ
0.005514	Intensity_Mean_ch1_noBackground	min	no	BiofilmQ
0.005502	counts (normalized intensity, threshold < 0.15)	std	no	MicroICS

score	feature name	aggregation type	found also in ranking according to the date among 81 features	tool
0.005084	normalized counts (normalized intensity, threshold < 0.15)	max	no	MicroICS
0.004991	intensity differences	q75	no	MicroICS
0.004587	Biofilm_AutoCorrelation_CorrelationLength2D_ch1	/	no	BiofilmQ
0.004457	Ncells (none declumping)	/	yes	BiofilmQ
0.00435	normalized counts (normalized intensity, threshold < 0.12)	max	no	MicroICS
0.004085	Shape_Volume_max (no declumping)	max	no	BiofilmQ
0.004079	Intensity_Integrated_ch1_noBackground	min	yes	BiofilmQ
0.00392	counts (normalized intensity, threshold < 0.06)	min	yes	BiofilmQ
0.003706	counts (normalized intensity, threshold < 0.04)	min	no	MicroICS
0.003619	Intensity_Integrated_ch1 (no declumping)	p75	no	BiofilmQ
0.00356	normalized counts (normalized intensity, threshold < 0.2)	max	yes	MicroICS
0.003536	normalized counts (normalized intensity, threshold < 0.17)	max	no	MicroICS
0.003519	Intensity_Integrated_ch1 (no declumping)	p25	no	BiofilmQ
0.00346	Biofilm_VolumePerSubstrate (no declumping)	/	no	BiofilmQ
0.002939	Surface_PerSubstrateArea	p25	no	BiofilmQ
0.00291	normalized counts (normalized intensity, threshold < 0.15)	var	no	MicroICS
0.002761	normalized counts (normalized intensity, threshold < 0.21)	max	no	MicroICS
0.002761	Cube_Surface	p75	no	BiofilmQ

score	feature name	aggregation type	found also in ranking according to the date among 81 features	tool
0.00276	counts (normalized intensity, threshold < 0.15)	median	no	MicroICS
0.002741	counts (normalized intensity, threshold < 0.14)	median	no	MicroICS
0.002696	normalized counts (normalized intensity, threshold < 0.16)	max	no	MicroICS
0.002593	normalized minimum	min	no	MicroICS
0.002278	Architecture_LocalSubstrateArea (no declumping)	max	no	BiofilmQ
0.002261	Texture_Haralick_Energy_ch1_range5	mean_biovolume	no	BiofilmQ
0.002179	counts (normalized intensity, threshold < 0.07)	min	no	MicroICS
0.002119	normalized counts (normalized intensity, threshold < 0.23)	max	no	MicroICS
0.002045	normalized counts (normalized intensity, threshold < 0.16)	var	no	MicroICS
0.001866	minimum	q90	no	MicroICS
0.001692	counts (threshold < 0.08)	q25	no	MicroICS
0.001644	Cube_Surface	mean	no	BiofilmQ
0.001518	Shape_Volume (no declumping)	p25	no	BiofilmQ
0.001488	normalized counts (normalized intensity, threshold < 0.14)	var	no	MicroICS
0.001485	Surface_LocalRoughness_range10	median	no	BiofilmQ
0.001457	Architecture_LocalSubstrateArea (no declumping)	std	no	BiofilmQ
0.001456	normalized counts (normalized intensity, threshold < 0.11)	max	no	MicroICS
0.001421	Shape_Volume	mean	no	BiofilmQ

score	feature name	aggregation type	found also in ranking according to the date among 81 features	tool
0.001411	Shape_Volume (no declumping)	mean_biovolume	no	BiofilmQ
0.0014	Cube_Surface	median	no	BiofilmQ
0.001396	Surface_PerSubstrateArea	mean_biovolume	no	BiofilmQ
0.001393	Shape_Volume	p75	no	BiofilmQ
0.001393	normalized counts (normalized intensity, threshold < 0.12)	var	no	MicroICS
0.001372	counts (normalized intensity, threshold < 0.17)	median	no	MicroICS
0.00135	normalized counts (normalized intensity, threshold < 0.22)	max	no	MicroICS
0.001347	Texture_Haralick_Entropy_ch1_range5	mean_biovolume	no	BiofilmQ
0.001336	normalized counts (normalized intensity, threshold < 0.25)	max	no	MicroICS
0.001334	Intensity_Mean_ch1	min	no	BiofilmQ
0.001303	normalized counts (normalized intensity, threshold < 0.19)	max	no	MicroICS
0.00125	normalized counts (normalized intensity, threshold < 0.13)	var	no	MicroICS

Figure S8. Representative images illustrate key biofilm structural features across strains and food extract treatments used to test inference for task identity and clinical association. For the control groups of strains L1764 and L628, images show their typical biofilm structures. L628 forms microcolonies that cover the substrate and grow in height as aggregates, while L1764 exhibits a more dispersed, scattered pattern. Upon treatment with food extracts, distinct changes consistent with model classification shifts are observed: L628 biofilms treated with steak tartare and milk extracts display disrupted structures with scattered bacterial clusters similar to L1764, while melon extract causes individual cells to spread throughout the image volume, growing like a lawn at the substrate. In contrast, L1764 biofilms maintain a consistent dispersed pattern across treatments, indicating strain-dependent structural stability. The images shown are compressed 3D stacks created using Zen software (Zeiss), which compresses all layers into a 2D projection with shadow effects to enhance visualisation. This method reduces the distortion inherent in full 3D renderings, but some spatial details are inevitably lost. These images visually reinforce understanding of the model's sensitivity to biofilm phenotypic variations and provide biological context for the classification probabilities and accuracies presented in Table S6.

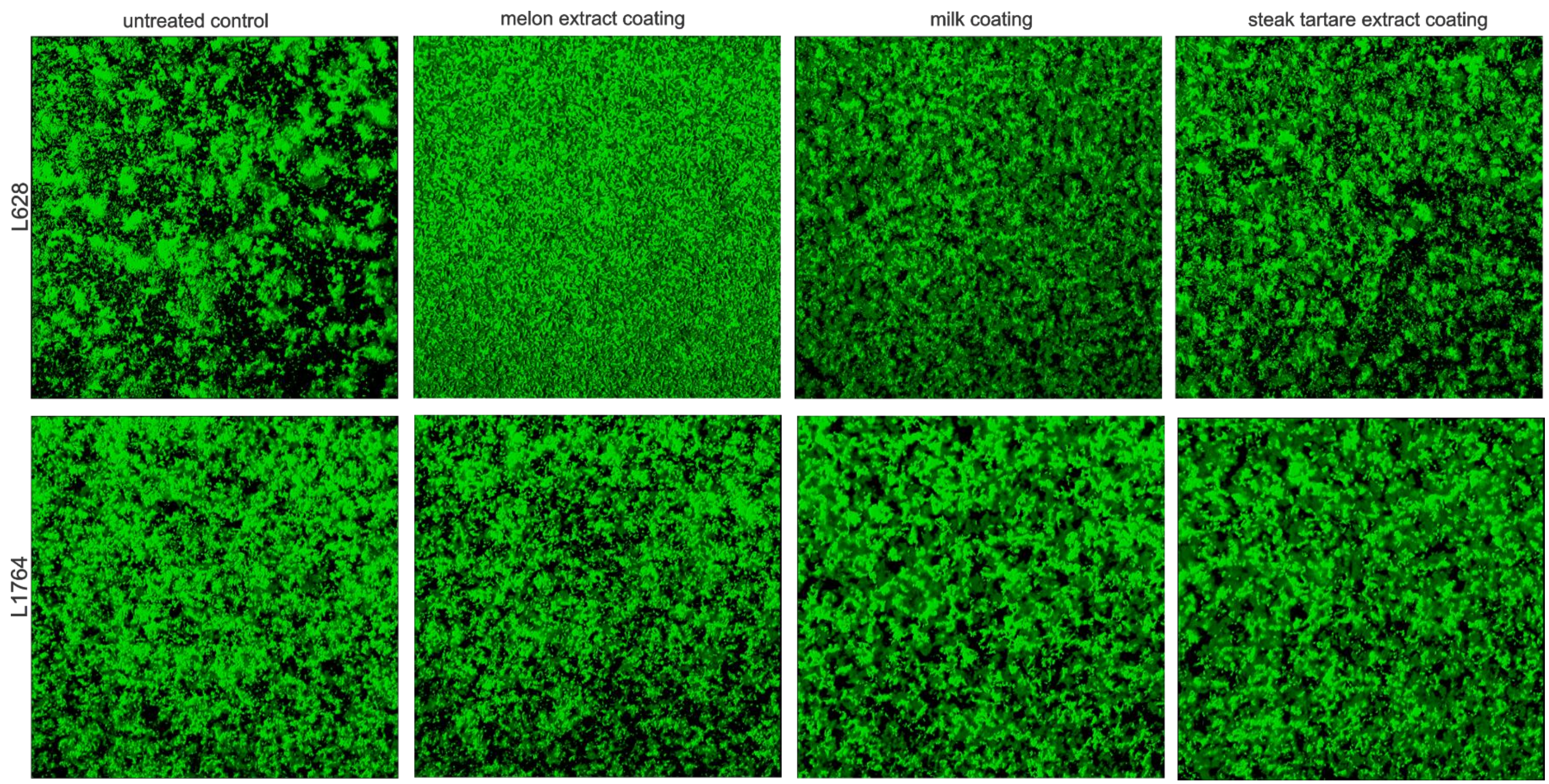

Table S6. Summary of inference results on biofilm images from two strains, L628 and L1764, whose 3D structures were modified using food extracts. Experiments were conducted on biofilms grown on melon, steak tartare extract, and milk, alongside untreated controls, over two independent days. Each day included 25 technical replicates per treatment, resulting in 50 images per condition. Inference was performed on each image, with results summarised by class below. The "strain" indicates the true class, particularly for controls, serving as an internal accuracy check. For treatments with food extracts, class switching was expected for L628 based on visual inspection – meaning the model predicted a class different from the true strain due to substantial 3D structural changes resembling another strain. The table shows the number of images assigned to each class from the training set; the class with the highest number of votes represents the majority vote and the final predicted class per treatment. The table also lists total correct and incorrect inferences, where “correctness” is defined by matching the true strain. Notably, L628 treated with food extracts showed a high number of “incorrect” classifications, strongly indicating class switching due to structural biofilm alterations.

		voted class								total per strain/treatment	
strain	treatment	**L1764**	**L455**	**L1323**	**L394**	**L628**	**ATCC 19115**	**L1823**	**L634**	**“correct”**	**“incorrect”**
L1764	control	**19**	2	1	3	0	0	0	0	19	6
L1764	melon extract coating	**46**	0	0	4	0	0	0	0	46	4
L1764	milk coating	**46**	0	0	2	0	0	2	0	46	4
L1764	steak tartare extract coating	**50**	0	0	0	0	0	0	0	50	0
L628	control	0	0	0	0	**24**	0	1	0	24	1
L628	melon extract coating	**21**	14	0	7	5	0	2	0	5	44
L628	milk coating	**42**	0	0	4	0	0	4	0	0	50
L628	steak tartare extract coating	**47**	0	0	1	0	0	2	0	0	50

Figure S9. Box and whisker plot analysis of L1764 probability distributions and complete class profiles across food extract treatments. Box plots show probability distributions for the L1764 strain and task identity in untreated and food extract-treated samples (melon, steak tartare, milk), along with individual measurements for all eight classes. Boxes represent the interquartile range (25th–75th percentiles) with thick black medians; thin black whiskers indicate the data range, and black dots mark outliers. Colored fills correspond to the class-specific color scheme (ATCC 19115, L628, L455, L634, L1323, L1823, L394, L1764).

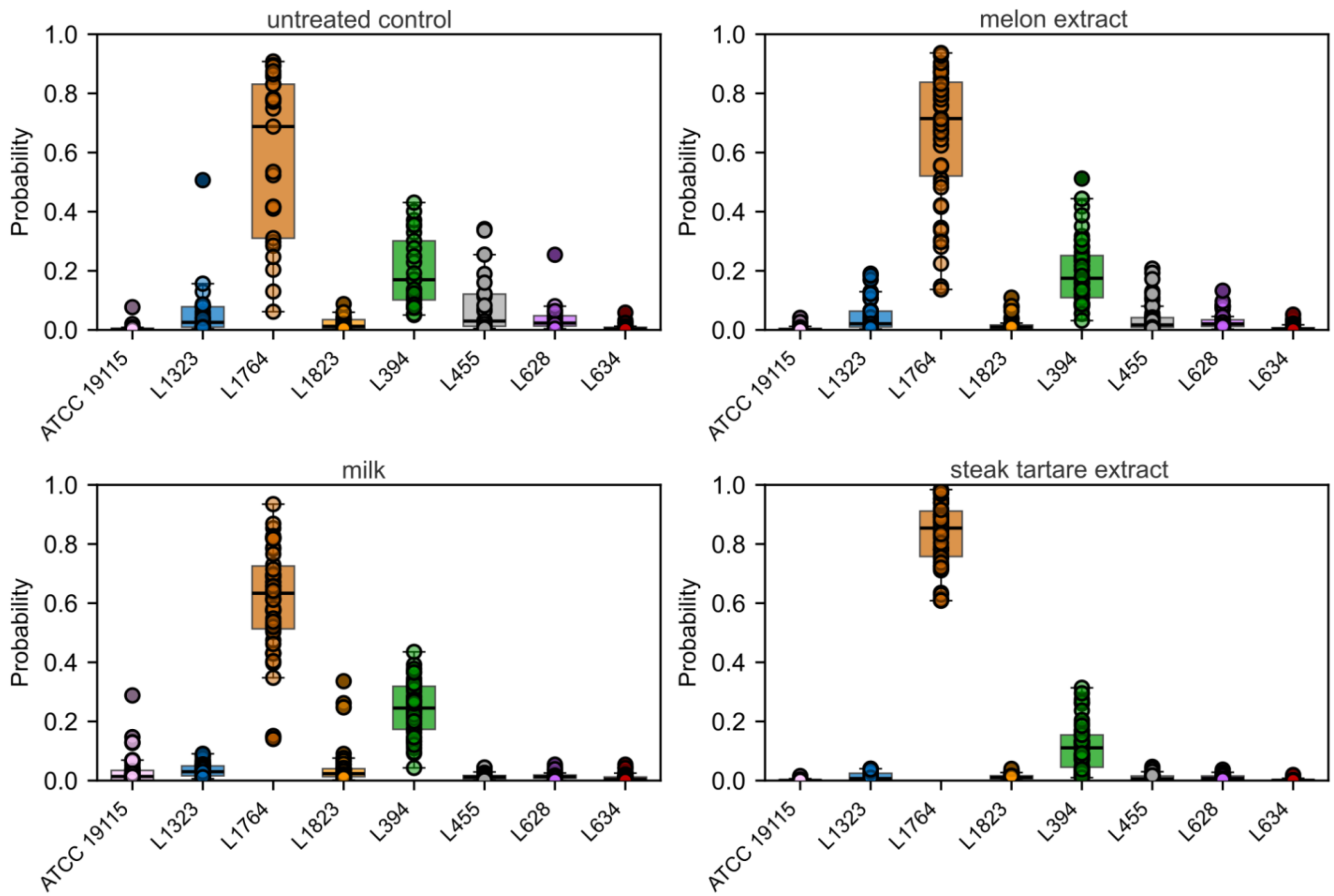

Figure S10. SHAP dependence plot for strain identity prediction on food-treated L1764 and L628 biofilms. The features shown have the strongest attribution and closely match those identified as most important during training (Table S5, feature ranking), demonstrating consistent reliance on textural features across experimental conditions despite treatment-induced structural shifts.

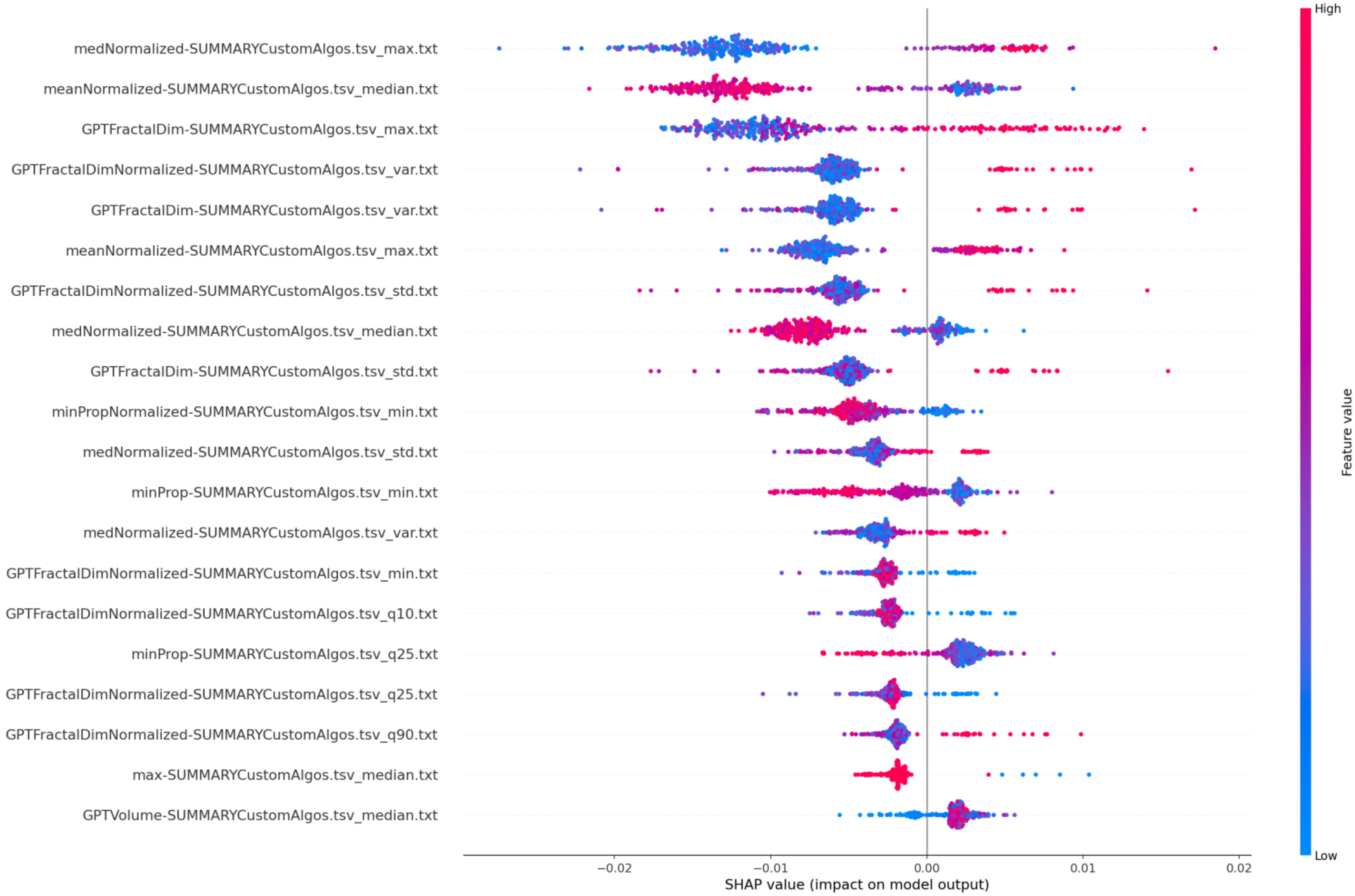

Figure S11. Classifier performance for clinical association prediction. Performance using all computed features as input (a; up to 512 dimensions for feature projections, 2700 dimensions for full feature set) and excluding the "count" feature with the same dimension projections for comparison (b).

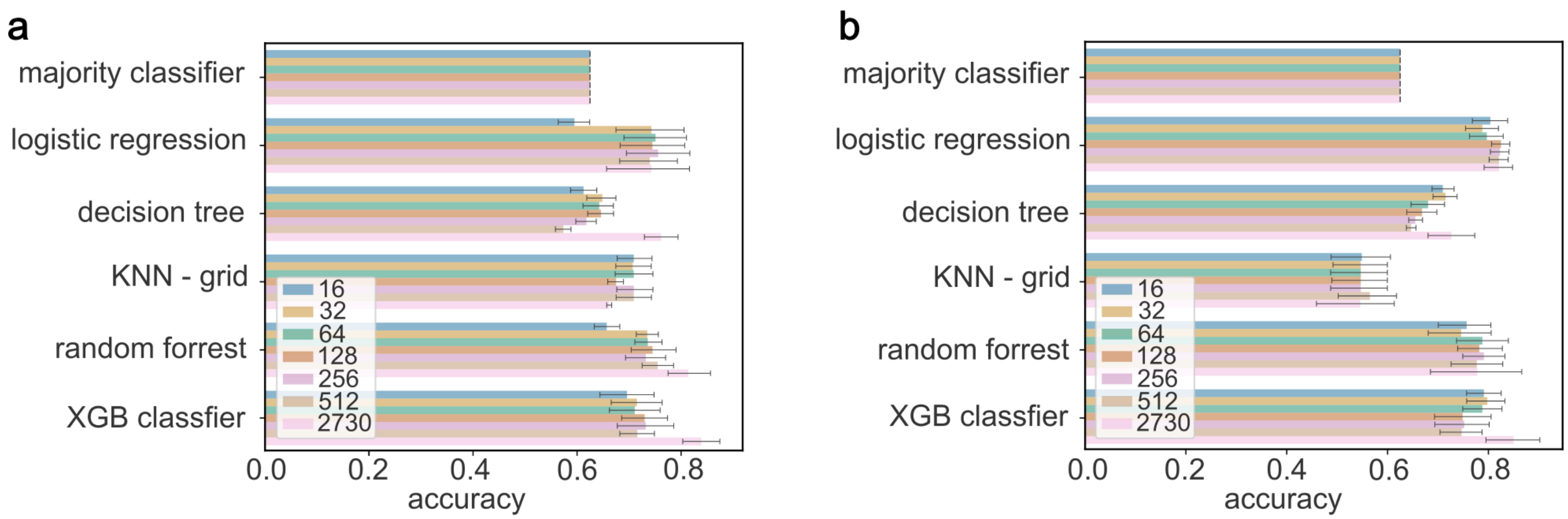

Table S7. The 61 most influential features for *L. monocytogenes* strain classification in the clinical association determination task were selected using random forest feature importance and ablation analysis. Features overlapping with those critical for predicting experimental date (batch effect) may indicate potential temporal or batch confounding. These should be interpreted with caution, as they may reflect methodological artefacts rather than true strain-specific biological differences.

score	feature name	aggregation type	found also in ranking according to the date among 81 features	calculated for layer or image
0.12869088	counts (normalized intensity, normalized per layer, threshold < 0.19)	max	no	yes
0.072151429	counts (normalized intensity, normalized per layer, threshold < 0.11)	median	no	yes
0.064598648	counts (normalized intensity, normalized per layer, threshold < 0.99)	median	no	yes
0.063460024	counts (threshold < 0.99)	q90	yes	yes
0.039658879	counts (normalized intensity, normalized per layer, threshold < 0.29)	max	no	yes
0.03096944	counts (threshold < 0.60)	std	no	yes
0.030553764	counts (normalized intensity, normalized per layer, threshold < 0.20)	max	no	yes
0.029908862	counts (normalized intensity, normalized per layer, threshold < 0.03)	max	no	yes
0.024690207	counts (threshold < 0.06)	var	no	yes
0.022889065	counts (normalized intensity, normalized per layer, threshold < 0.12)	median	no	yes
0.019007596	counts (normalized intensity, normalized per layer, threshold < 0.26)	median	no	yes
0.016875062	counts (normalized intensity, normalized per layer, threshold < 0.27)	max	no	yes
0.016713746	counts (normalized intensity, normalized per layer, threshold < 0.25)	max	no	yes
0.015614576	counts (normalized intensity, normalized per layer, threshold < 0.18)	max	no	yes
0.015069583	counts (normalized intensity, normalized per layer, threshold < 0.21)	max	no	yes

score	feature name	aggregation type	found also in ranking according to the date among 81 features	calculated for layer or image
0.014517251	counts (normalized intensity, normalized per layer, threshold < 0.15)	max	no	yes
0.012122997	counts (normalized intensity, normalized per layer, threshold < 0.17)	max	no	yes
0.011378805	counts (normalized intensity, normalized per layer, threshold < 0.25)	median	no	yes
0.01130189	counts (normalized intensity, normalized per layer, threshold < 0.22)	max	no	yes
0.011252359	counts (normalized intensity, normalized per layer, threshold < 0.16)	max	no	yes
0.009005039	counts (normalized intensity, normalized per layer, threshold < 0.14)	median	no	yes
0.008579546	counts (normalized intensity, normalized per layer, threshold < 0.13)	median	no	yes
0.006408293	counts (normalized intensity, normalized per layer, threshold < 0.13)	max	no	yes
0.006158488	counts (normalized intensity, normalized per layer, threshold < 0.28)	max	no	yes
0.00604514	counts (threshold < 0.07)	std	no	yes
0.006016844	counts (normalized intensity, normalized per layer, threshold < 0.14)	max	no	yes
0.005892313	normalized GTP volume	q90	yes	yes
0.005828085	counts (normalized per layer, threshold < 0.07)	q90	no	yes
0.005761363	counts (threshold < 0.06)	q90	yes	yes
0.005562963	counts (normalized intensity, normalized per layer, threshold < 0.17)	median	no	yes
0.005526274	counts (normalized per layer, threshold < 0.02)	q25	no	yes
0.005375397	counts (threshold < 0.07)	max	no	yes
0.005353913	counts (normalized intensity, threshold < 0.22)	median	no	yes

score	feature name	aggregation type	found also in ranking according to the date among 81 features	calculated for layer or image
0.004206484	standard deviation of intensity	std	no	yes
0.004057222	counts (threshold < 0.27)	median	no	yes
0.003477023	standard deviation of intensity	var	no	yes
0.003375756	counts (normalized per layer, threshold < 0.02)	min	no	yes
0.003316901	counts (normalized intensity, normalized per layer, threshold < 0.26)	max	no	yes
0.00324162	counts (normalized intensity, normalized per layer, threshold < 0.09)	median	no	yes
0.003027268	counts (normalized intensity, normalized per layer, threshold < 0.23)	max	no	yes
0.00300337	counts (normalized intensity, normalized per layer, threshold < 0.15)	median	no	yes
0.002964209	counts (normalized intensity, normalized per layer, threshold < 0.18)	median	no	yes
0.002940346	counts (normalized intensity, threshold < 0.21)	median	no	yes
0.002788009	normalized GTP fractal dimension	q10	yes	yes
0.002701132	counts (normalized intensity, threshold < 0.02)	q90	no	yes
0.002564346	counts (threshold < 0.01)	q75	no	yes
0.002554472	counts (threshold < 0.07)	var	no	yes
0.002522512	counts (threshold < 0.08)	max	yes	yes
0.002505103	counts (normalized intensity, threshold < 0.18)	median	no	yes
0.00217407	maximum intensity	q75	no	yes
0.002101751	counts (normalized intensity, threshold < 0.03)	q90	no	yes

score	feature name	aggregation type	found also in ranking according to the date among 81 features	calculated for layer or image
0.002098015	counts (normalized intensity, normalized per layer, threshold < 0.02)	max	no	yes
0.002079697	counts (threshold < 0.03)	q10	no	yes
0.002029671	counts (threshold < 0.26)	median	no	yes
0.001949286	counts (threshold < 0.24)	median	yes	yes
0.001881967	counts (threshold < 0.06)	min	no	yes
0.001860008	counts (normalized per layer, threshold < 0.02)	q10	no	yes
0.001859035	normalized GTP fractal dimension	min	yes	yes
0.001848041	counts (threshold < 0.17)	median	no	yes
0.001781677	counts (threshold < 0.04)	q10	no	yes
0.001675974	counts (threshold < 0.03)	std	no	yes

Figure S12. The SHAP dependence plot for clinical association prediction on food-treated L1764 and L628 biofilms shows a shift toward intensity-based features compared to the training feature rankings (Table S7). GPT fractal dimension and void fraction (min prop) have also strong attribution, while GPT volume remains important, reflecting partial feature continuity amid the observed shift from abundance to texture-based discrimination.

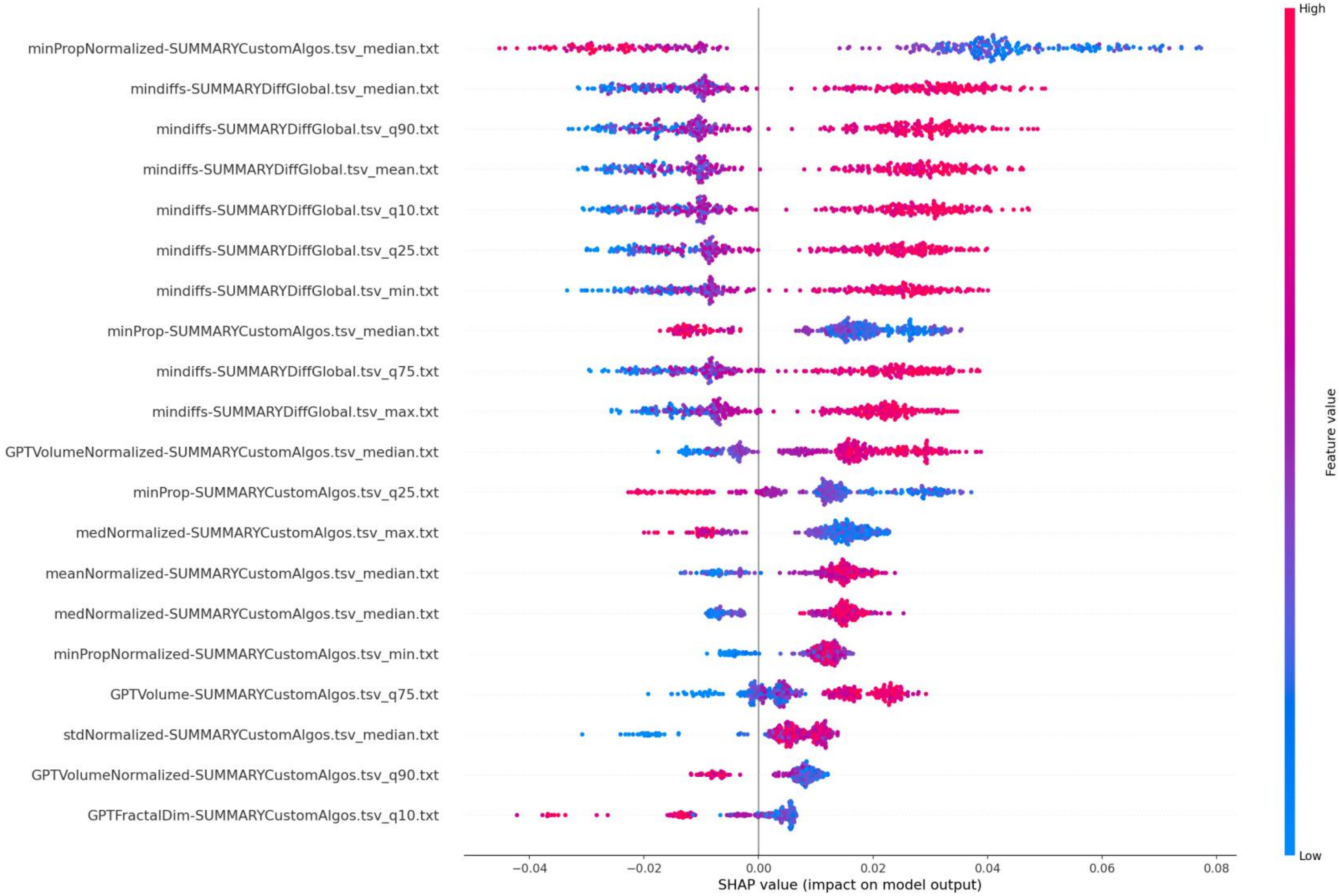

Supplement: Supplementary file 1 [file 41522_2026_1083_MOESM1_ESM.pdf]
